# Diagonalizing Bose Gases in the Gross–Pitaevskii Regime and Beyond

**DOI:** 10.1007/s00220-024-05199-w

**Published:** 2024-12-18

**Authors:** Morris Brooks

**Affiliations:** University of Zurich, Zurich, Switzerland

## Abstract

We present a novel approach to the Bogoliubov theory of dilute Bose gases, allowing for an elementary derivation of the celebrated Lee–Huang–Yang formula in the Gross–Pitaevskii regime. Furthermore, we identify the low lying excitation spectrum beyond the Gross–Pitaevskii scaling, extending a recent result (Brennecke et al. in Rev Math Phys 34, 2022) to significantly more singular scaling regimes. Finally, we provide an upper bound on the ground state energy in the Gross–Pitaevskii regime that captures the correct expected order of magnitude beyond the Lee–Huang–Yang formula.

## Introduction

Given a non-negative radial function with compact support $V\in L^1 \! \left( {\mathbb {R}}^3\right)$ let us introduce the rescaled two-particle interaction $V_L(x,y):=L^2 V\!\left( L(x-y)\right)$, where $L>0$. In the following we will study the many-particle Hamilton operator $H_{N,\kappa }$ acting on the *N*-particle Hilbert space $L^2_{\textrm{sym}} \! \left( \Lambda ^N\right)$, with $\Lambda :=\left[ -\frac{1}{2},\frac{1}{2}\right] ^3$ being the three-dimensional torus, given by

1 $$\begin{aligned} H_{N,\kappa }&:= \sum _{i=1}^N (-\Delta )_{x_i}+\sum _{1\le i<j\le N}V_{N^{1-\kappa }}(x_i,x_j)\nonumber \\&=\sum _{k\in 2\pi {\mathbb {Z}}^3} |k|^2 a_k^\dagger a_k \! + \! \frac{1}{2}\sum _{jk,mn\in 2\pi {\mathbb {Z}}^3}\left( V_{N^{1-\kappa }}\right) _{jk,mn}a_k ^\dagger a_j^\dagger a_m a_n, \end{aligned}$$

where $a_k$ and $a_k^\dagger$ are the standard annihilation and creation operators on the bosonic Fock space ${\mathcal {F}}:=\bigoplus _{n=0}^\infty L^2_{\textrm{sym}} \! \left( \Lambda ^n\right)$ corresponding to the modes $e^{ikx}$ for $k\in 2\pi {\mathbb {Z}}^3$, $x_i-x_j$ refers to the distance between two particles on the torus $\Lambda$ and $0\le \kappa < \frac{2}{3}$ is an additional scaling parameter. If not indicated otherwise, we will always assume that indices run in the set $2\pi {\mathbb {Z}}^3$, which we will usually neglect in our notation, and we write $k\ne 0$ in case the index runs in the set $2\pi {\mathbb {Z}}^3{\setminus } \{0\}$.

The study of the low energy properties, such as the ground state energy $E_{N,\kappa }$, of dilute Bose gases described by the Hamilton operator $H_{N,\kappa }$ has a long standing history in the mathematics, as well the physics, literature. A rigorous derivation of the leading order

2 $$\begin{aligned} E_{N,\kappa }=4\pi {\mathfrak {a}} N^{1+\kappa }+o_{N\rightarrow \infty } \! \left( N^{1+\kappa }\right) , \end{aligned}$$

has been achieved in [18] for the thermodynamic limit in the regime of small densities $\rho$, where $\kappa$ is determined by the density $\rho$ via $\rho =N^{3\kappa -2}$, and later for the Gross–Pitaevskii regime $\kappa :=0$ in [17], with the scattering length ${\mathfrak {a}}$ defined as $8\pi {\mathfrak {a}}:=\int _{{\mathbb {R}}^3} V(x)\varphi (x)\textrm{d}x$, where $\varphi$ is the radial solution of $(-2\Delta +V)\varphi =0$ subject to the boundary condition $\varphi \underset{|x|\rightarrow \infty }{\longrightarrow }\ 1$. More recently the subleading contribution to the asymptotics in Eq. (2), famously conjectured to be the Lee–Huang–Yang term emerging from the underlying Bogoliubov theory [3, 13], has been identified in the Gross–Pitaevskii regime in [1] and in the thermodynamic limit in [8, 9]. Following these landmark results, a lot of effort has been made to streamline and generalize the methods and results, see for example [2, 4, 7, 10, 12].

In this work we want to combine the approach in [7–9] with the one in [1, 12], with the goal of finding an algebraic method, such as the one in [7–9], with a clean representation of important cancellations comparable to the one in [12]. Furthermore, we will follow an alternative approach when it comes to the scattering length, which will be introduced via the Feshbach-Schur map discussed in Sect. 2. The following three hand-selected Theorem 1, Theorem 2 and Theorem 3 should be seen as an advertisement for the general method and its robustness, highlighting its various advantages. Starting with Theorem 1, we recover the, at this point, well known results in [1, 12], following, what we would call, a short and elementary proof. All the relevant cancellations within the proof are exclusively discussed in Sect. 2.

### Theorem 1

Let $E_N$ be the ground state energy of the operator $H_{N}:=H_{N,0}$. Then we have

3 $$\begin{aligned} E_N= 4\pi {\mathfrak {a}}_N (N-1)+\frac{1}{2}\sum _{k\ne 0}\left\{ \sqrt{|k|^4+16\pi {\mathfrak {a}} |k|^2}-|k|^2-8\pi {\mathfrak {a}}+\frac{(8\pi {\mathfrak {a}})^2}{2|k|^2}\right\} +o_{N\rightarrow \infty }(1), \end{aligned}$$

where ${\mathfrak {a}}_N$ is the box scattering length defined in Eq. (10).

While Eq. (3) claims an identity, our proof in Sect. 3.1 only concerns the lower bound, as the upper bound is discussed in detail in Theorem 3. Besides commenting on the length and complexity of the proof of Theorem 1, we also want to emphasise that our proof of Theorem 1 and Theorem 2, and especially Theorem 3, do not require any cut-off parameters. However, it can be useful to introduce such a parameter anyways, as it shortens the proof of Theorem 1 and allows us to slightly extend the range of possible values $\kappa$ in case of Theorem 2, to be precise we can verify Theorem 2 without any cut-off parameter for $0\le \kappa <\frac{1}{11}$. Furthermore, we want to point out that the error terms in the proof of the lower bound in Theorem 1 can be controlled by the particle number operator alone, and do not require the kinetic energy.

In the subsequent Theorem 2 we extend the results in [4] concerning the excitation spectrum of the operator $H_{N,\kappa }$ for $0\le \kappa <\kappa _0$, where $\kappa _0$ is of the order of magnitude $10^{-3}$, to the significantly large range $0\le \kappa <\frac{1}{8}$. We do however want to emphasise, that our proof of Theorem 2 relies on strong a priori estimates provided by recent results in [6], and later in a slightly different setting by [2, 10].

### Theorem 2

Let $E_{N,\kappa }$ be the ground state energy of $H_{N,\kappa }$, $0\le \kappa <\frac{1}{8}$ and assume that *V* is radially symmetric decreasing. With the definition $\tau :=\frac{5}{2}\! \left( \frac{1}{8}-\kappa \right) >0$, $E_{N,\kappa }$ is given by

4 $$\begin{aligned}&4\pi {\mathfrak {a}}_{N^{1-\kappa }}N^{\kappa } (N \! - \! 1) \! + \! \frac{1}{2} \! \sum _{k\ne 0} \! \left\{ \! \sqrt{|k|^4 \! + \! 16\pi {\mathfrak {a}} N^{\kappa }|k|^2} \! - \! |k|^2 \! - \! 8\pi {\mathfrak {a}} N^{\kappa } \! + \! \frac{(8\pi {\mathfrak {a}} N^{\kappa })^2}{2|k|^2} \! \right\} \! \nonumber \\  &\qquad + \! O_{N\rightarrow \infty }\! \left( N^{-\tau }\right) . \end{aligned}$$

Let furthermore $E_{N,\kappa }^{(d)}$ denote the *d*-th eigenvalue, in increasing order, and let us enumerate the set $\left\{ \sum _{k\ne 0}n_k \sqrt{|k|^4+16\pi {\mathfrak {a}} N^\kappa |k|^2}: n_k\in {\mathbb {N}}_0\right\}$ in increasing order by $\{\lambda _{N,\kappa }^{(1)}, \lambda _{N,\kappa }^{(2)},\dots \}$. Then

$$\begin{aligned} E^{(d)}_{N,\kappa } -E_{N,\kappa } = \lambda _{N,\kappa }^{(d)} + O_{N\rightarrow \infty }\! \left( N^{-\tau }\right) . \end{aligned}$$

Again, the proof of Theorem 2 focuses on the lower bound, as the upper bound is discussed in a more general setting in the subsequent Theorem 3. We want to point out here, that even though our algebraic manipulations do not make use of unitary operations, they are equally well suited to provide lower bounds and upper bounds on the ground state energy as well as the excitation spectrum. Furthermore, we want to note that our resolution of the spectrum up to the order $N^{-\tau }$ is sharp enough to see the non-linear contribution of $|k|^4$ in the excitations $\sqrt{|k|^4+16\pi {\mathfrak {a}} N^\kappa |k|^2}$ for the slightly smaller range $0\le \kappa <\frac{5}{48}$.

In our final Theorem 3, we provide sufficient upper bounds on the excitation energy $E_{N,\kappa }^{(d)}$ in order to conclude the proof of Theorem 1 and Theorem 2. Furthermore, we derive an improved, and novel, upper bound on the ground state energy $E_N$ in the Gross–Pitaevskii regime, which gives a correction of the order $\frac{\log N}{N}$. We want to emphasise that the scale $\frac{\log N}{N}$ is indeed the expected order of magnitude for the next term in the asymptotic expansion in Eq. (3), see [21], and we believe it is crucial at this point that our method does not rely on cut-off techniques in momentum space.

### Theorem 3

In the Gross–Pitaevskii regime $\kappa :=0$, we have the upper bound

5 $$\begin{aligned} E_{N} \le 4\pi {\mathfrak {a}}_N (N-1)+\frac{1}{2}\sum _{k\ne 0}\left\{ \sqrt{|k|^4+16\pi {\mathfrak {a}} |k|^2}-|k|^2-8\pi {\mathfrak {a}}+\frac{(8\pi {\mathfrak {a}})^2}{2|k|^2}\right\} +C \frac{\log N}{N} \end{aligned}$$

for a suitable $C>0$. Furthermore, for $\kappa <\frac{2}{13}$ and $d\in {\mathbb {N}}$, $E_{N,\kappa }^{(d)}$ is bounded from above by

6 $$\begin{aligned}&4\pi {\mathfrak {a}}_N N^\kappa (N \! - \! 1) \! + \! \frac{1}{2}\sum _{k\ne 0}\left\{ \sqrt{|k|^4 \! + \! 16\pi {\mathfrak {a}} N^\kappa |k|^2} \! - \! |k|^2 \! - \! 8\pi {\mathfrak {a}} N^\kappa \! + \! \frac{(8\pi {\mathfrak {a}} N^\kappa )^2}{2|k|^2}\right\} \! + \! \lambda _{N,\kappa }^{(d)} \! \nonumber \\  &\qquad + \! C N^{\frac{13\kappa }{4}-\frac{1}{2}}. \end{aligned}$$

## The Two-Body Problem

Before we come to the (approximate) diagonalization of the many-body operator $H_{N,\kappa }$, which will be the basis of verifying Theorem 1, Theorem 2 and Theorem 3, we will first investigate the corresponding problem for the two body Hamiltonian

$$\begin{aligned} H:=-\Delta _2+V_L(x,y) \end{aligned}$$

defined on $L^2\! \left( \Lambda ^2\right)$ with $\Delta _2:=\Delta _x+\Delta _y$. The goal of this Section is to find a transformation

$$\begin{aligned} S:L^2\! \left( \Lambda ^2\right) \longrightarrow L^2\! \left( \Lambda ^2\right) , \end{aligned}$$

which removes correlations of *H* between low energy momentum pairs $(k_1,k_2)\in {\mathcal {L}}\subseteq \left( 2\pi {\mathbb {Z}}\right) ^6$ and high energy momentum pairs ${\mathcal {H}}:=\left( 2\pi {\mathbb {Z}}\right) ^6{\setminus } {\mathcal {L}}$. We will specify the set ${\mathcal {L}}$ later this section, for now let $\pi _{{\mathcal {L}}}$ and $\pi _{{\mathcal {H}}}=1-\pi _{{\mathcal {L}}}$ denote the corresponding projections in $L^2\! \left( \Lambda ^2\right)$. With $\pi _{\mathcal {L}}$ and $\pi _{\mathcal {H}}$ at hand, *H* can be represented as a block matrix

$$\begin{aligned} H=\begin{bmatrix} \pi _{\mathcal {L}} (-\Delta _2+V_L)\pi _{\mathcal {L}} &  \quad \pi _{\mathcal {L}} V_L\pi _{\mathcal {H}} \\ \pi _{\mathcal {H}} V_L\pi _{\mathcal {L}} & \quad \pi _{\mathcal {H}} (-\Delta _2+V_L)\pi _{\mathcal {H}} \end{bmatrix}. \end{aligned}$$

It is now an elementary task in linear algebra to remove the correlation terms $\pi _{\mathcal {H}} V_L\pi _{\mathcal {L}}$ and $\pi _{\mathcal {L}} V_L\pi _{\mathcal {H}}$, and therefore bringing the operator *H* in a block diagonal form. For this purpose let us define the Feshbach-Schur map

$$\begin{aligned} S:=1-R V_L\pi _{\mathcal {L}}=\begin{bmatrix} 1 & \quad 0 \\ -R V_L\pi _{\mathcal {L}} &  \quad 1 \end{bmatrix}, \end{aligned}$$

with *R* being the pseudo-inverse of $\pi _{\mathcal {H}} (-\Delta _2+V_L)\pi _{\mathcal {H}}$, and compute

7 $$\begin{aligned} S^\dagger H S&=\begin{bmatrix} \pi _{\mathcal {L}} \left( -\Delta _2+V_L-V_L R V_L\right) \pi _{\mathcal {L}} &  \quad 0 \\ 0 & \quad \pi _{\mathcal {H}} (-\Delta _2+V_L)\pi _{\mathcal {H}} \end{bmatrix}, \end{aligned}$$

where we have used the cancellation between the terms

$$\begin{aligned} \pi _{\mathcal {H}}\left( S^\dagger HS\right) \pi _{\mathcal {L}}&=\pi _{\mathcal {H}} HS\pi _{\mathcal {L}}=\pi _{\mathcal {H}} H\pi _{\mathcal {L}}-\pi _{\mathcal {H}}H R V_L\pi _{\mathcal {L}}\\  &=\pi _{\mathcal {H}} V_L\pi _{\mathcal {L}}-\pi _{\mathcal {H}}H R V_L\pi _{\mathcal {L}}=0. \end{aligned}$$

Consequently, we have the identity

8 $$\begin{aligned} H=T^{\dagger }\left( -\Delta _2\right) T+T^\dagger {\widetilde{V}}_LT, \end{aligned}$$

where $$T:=1+R V_L\pi _{\mathcal {L}}=\begin{bmatrix} 1 & \quad 0 \\ R V_L\pi _{\mathcal {L}} & \quad 1 \end{bmatrix}$$ is the inverse of the Feshbach-Schur map *S* and the renormalized potential is defined as

9 $$\begin{aligned} {\widetilde{V}}_L:=\begin{bmatrix} \pi _{\mathcal {L}} \left( V_L-V_L R V_L\right) \pi _{\mathcal {L}} &  \quad 0 \\ 0 &  \quad \pi _{\mathcal {H}} V_L \pi _{\mathcal {H}} \end{bmatrix}. \end{aligned}$$

In the following we will always use the concrete choice ${\mathcal {L}}:=\bigcup _{|k|<K}\{( k,0),(0, k)\}$, for a given $0< K\le \infty$. At this point we want to emphasise that in the case $K=\infty$, the definition of *T* and ${\widetilde{V}}_L$ do not include any cut-off parameter in momentum space.

We can now relate the renormalized potential $V_{L}-V_{L} R V_{L}$ with the scattering properties of the potential *V*(*x*). Following [12], let us first define the box scattering length

10 $$\begin{aligned} {\mathfrak {a}}_L:=\frac{L}{8\pi }\Big (V_{L}-V_{L} R V_{L}\Big )_{0 0 ,00}=\frac{1}{8\pi }\left( \int V(x)\textrm{d}x-L{\langle {V_L,R V_L}\rangle }\right) , \end{aligned}$$

which is independent of the choice of *K*. It has been shown in [11] that the box scattering length satisfies $|{\mathfrak {a}}_L-{\mathfrak {a}}|\lesssim L^{-1}$, where ${\mathfrak {a}}$ is the scattering length of *V*(*x*) introduced in Sect. 1. Furthermore, also the other matrix entries of the renormalized potential $V_{L}-V_{L} R V_{L}$ appearing in Eq. (9) can be related to the scattering length as we demonstrate in Lemma 1.

### Lemma 1

Let $V\in L^1 \! \left( {\mathbb {R}}^3\right)$. Then there exists a constant $D_V$ such that for all $k_i\in 2\pi {\mathbb {Z}}^3$ satisfying momentum conservation $k_1+k_2=k_3+k_4$

11 $$\begin{aligned} \left| L\Big (V_{L}-V_{L} R V_{L}\Big )_{k_1 k_2, k_3 k_4}-8\pi {\mathfrak {a}} \right| \le D_V L^{-1} \! \left( \! 1+\sum _{i=1}^4 |k_i| \! \right) , \end{aligned}$$

and $\left| L\Big (V_{L}-V_{L} R V_{L}\Big )_{k_1 k_2, k_3 k_4}\right| \le D_V$ uniformly in $k_i$. All estimates are uniform in the parameter *K* introduced below Eq. (9), and for any $v\ge 0$ there exists a $D>0$ such that $D_V\le D$ for all *V* with $\Vert V\Vert _{L^1 \! \left( {\mathbb {R}}^3\right) }\le v$.

In order to keep the proof of Lemma 1 in the Appendix A self-contained, we will provide a proof of the fact that $|{\mathfrak {a}}_L-{\mathfrak {a}}|\lesssim L^{-1}$ as well in Lemma 12. We want to emphasize at this point that with the exception of Sect. 5 and Appendix C, all the bounds in this manuscript depend only on the constant $D_V$ in Lemma 1, in the sense that for all $D>0$, the estimates are uniform for potentials $V\in L^1 \! \left( {\mathbb {R}}^3\right)$ satisfying

12 $$\begin{aligned} D_V\le D. \end{aligned}$$

A similar observation has been used in [9] in order to analyze hard core interactions. While it seems reasonable to expect that $D_V$ is bounded even for hard core interactions, we restrict our attention to $V\in L^1 \! \left( {\mathbb {R}}^3\right)$.

## The Many-Body Diagonalization

It is the goal of this Section to lift the diagonalization procedure for the two-particle Hamiltonian derived in Sect. 2 to a (block) diagonalization of the many-body Hamiltonian

13 $$\begin{aligned} H_{N,\kappa } =\sum _{k\in 2\pi {\mathbb {Z}}^3} |k|^2 a_k^\dagger a_k \! + \! \frac{1}{2}\sum _{jk,mn\in 2\pi {\mathbb {Z}}^3}\left( V_{N^{1-\kappa }}\right) _{jk,mn}a_k ^\dagger a_j^\dagger a_m a_n, \end{aligned}$$

allowing us to absorb correlations between low-momentum pairs $(jk)\in {\mathcal {L}}$ and high-momentum pairs $(mn)\in {\mathcal {H}}$ by completing suitable squares involving high-momentum pairs. On a technical level, this will be done by the introduction of a many-body counterpart to the inverse Feshbach-Schur map $T:L^2\! \left( \Lambda ^2\right) \longrightarrow L^2\! \left( \Lambda ^2\right)$ defined below Eq. (7), which is realized by the new sets of operators $c_k$ and $\psi _{j k}$ defined on the Fock space ${\mathcal {F}}$ as

14 $$\begin{aligned} c_k&:= a_k+\sum _{j , mn}\left( T-1\right) _{jk, mn} a_j^\dagger a_m a_n, \end{aligned}$$

15 $$\begin{aligned} \psi _{j k}&:= a_j a_k+\sum _{mn}\left( T-1\right) _{j k, m n} a_m a_n. \end{aligned}$$

The following Lemma 2 is the many-particle counterpart to Eq. (8), in the sense that it provides an (approximate) block-diagonal representation of the operator $H_{N,\kappa }$ in terms of the new variables $c_k$ and $\psi _{j k}$.

### Lemma 2

Let ${\widetilde{V}}_{L}$ be the renormalized potential defined in Eq. (9). Then

16 $$\begin{aligned} H_{N,\kappa }=\sum _k |k|^2 c_k^\dagger c_k \! + \! \frac{1}{2}\sum _{jk,mn}\left( {\widetilde{V}}_{N^{1-\kappa }}\right) _{jk,mn}\psi _{j k}^\dagger \psi _{m n}-{\mathcal {R}}, \end{aligned}$$

where the residual operator ${\mathcal {R}}$ is defined as

$$\begin{aligned} {\mathcal {R}}:=\sum _{jk,mn;j',m'n'}|k|^2 \overline{(T-1)_{j' k,m'n'}}(T-1)_{j k,m n} a_{n'}^\dagger a_{m'}^\dagger a_j^\dagger a_{j'}a_m a_n. \end{aligned}$$

### Proof

Using the canonical commutation relations (CCR) of the operators $a_{j'}$ and $a_j^\dagger$

$$\begin{aligned} \left[ a_j',a_j^\dagger \right] := a_{j'}a_j^\dagger -a_j^\dagger a_{j'} =\delta _{j',j}, \end{aligned}$$

and the permutation symmetry of the coefficients $T_{jk,mn}=T_{kj,nm}$, we compute

17 $$\begin{aligned} \sum _k \! |k|^2 (c_k \! - \! a_k)^\dagger (c_k \! - \! a_k)&=\sum _{jk,mn;j',m'n'}|k|^2 \overline{(T-1)_{j' k,m'n'}}(T-1)_{j k,m n} a_{n'}^\dagger a_{m'}^\dagger a_{j'} a_j^\dagger a_m a_n\nonumber \\&= \sum _{jk,mn;m'n'}|k|^2 \overline{(T-1)_{j k,m'n'}}(T-1)_{j k,m n} a_{n'}^\dagger a_{m'}^\dagger a_m a_n + {\mathcal {R}}\nonumber \\&= \! \frac{1}{2} \! \sum _{mn;m'n'} \! \! \sum _{jk} \! \big (|k|^2 \! + \! |j|^2\big ) \overline{(T \! - \! 1)_{j k,m'n'}}(T \! - \! 1)_{j k,m n}\, a_{n'}^\dagger a_{m'}^\dagger a_m a_n \! + \! {\mathcal {R}}\nonumber \\&= \frac{1}{2}\sum _{mn;m'n'}\left( (T-1)^\dagger (-\Delta _2)(T-1)\right) _{mn,m' n'} a_{n'}^\dagger a_{m'}^\dagger a_m a_n + {\mathcal {R}}. \end{aligned}$$

In a similar fashion, we compute the identities

18 $$\begin{aligned} \sum _k |k|^2 a_k^\dagger (c_k-a_k)+\mathrm {H.c.}&=\frac{1}{2}\sum _{mn;m'n'}\left( (-\Delta _2)(T-1)+\mathrm {H.c.}\right) _{mn,m' n'} a_{n'}^\dagger a_{m'}^\dagger a_m a_n, \end{aligned}$$

19 $$\begin{aligned} \sum _{jk,mn}\left( {\widetilde{V}}_{N^{1-\kappa }}\right) _{jk,mn}\psi _{j k}^\dagger \psi _{m n}&= \sum _{jk,mn}\left( T^\dagger {\widetilde{V}}_{N^{1-\kappa }}T\right) _{jk,mn} a_k^\dagger a_{j }^\dagger a_m a_n, \end{aligned}$$

where $\mathrm {H.c.}$ refers to the adjoint operator. Making use of

$$\begin{aligned} (T-1)^\dagger (-\Delta _2)(T-1)+\left\{ (-\Delta _2)(T-1)+\mathrm {H.c.}\right\} =T^\dagger (-\Delta _2)T+\Delta _2, \end{aligned}$$

we obtain by Eq. (17), Eq. (18) and Eq. (19)

$$\begin{aligned}&\sum _k |k|^2 c_k^\dagger c_k \! + \! \frac{1}{2}\sum _{jk,mn}\left( {\widetilde{V}}_{N^{1-\kappa }}\right) _{jk,mn}\psi _{j k}^\dagger \psi _{m n}\\&\quad = \sum _k |k|^2 a_k^\dagger a_k + \frac{1}{2}\sum _{jk,mn}\left\{ T^\dagger \left( -\Delta _2 + {\widetilde{V}}_{N^{1-\kappa }}\right) T+\Delta _2\right\} _{jk,mn} a_k^\dagger a_{j }^\dagger a_m a_n+{\mathcal {R}}. \end{aligned}$$

This concludes the proof by Eq. (8) derived in Sect. 2 and the representation of the operator $H_{N,\kappa }$ in Eq. (1). $\square$

The advantage of the representation in Eq. (16) compared to Eq. (13) is that the renormalized potential ${\widetilde{V}}_{N^{1-\kappa }}$ is in the block diagonal structure

$$\begin{aligned} {\widetilde{V}}_{N^{1-\kappa }}=\pi _{{\mathcal {L}}} (V_{N^{1-\kappa }}-V_{N^{1-\kappa }} R V_{N^{1-\kappa }})\pi _{{\mathcal {L}}}+\pi _{{\mathcal {H}}}V_{N^{1-\kappa }} \pi _{{\mathcal {H}}} , \end{aligned}$$

see the definition of ${\widetilde{V}}_{N^{1-\kappa }}$ in Eq. (9), and therefore we have removed correlations between low-momentum pairs $(jk)\in {\mathcal {L}}$ and high-momentum pairs $(mn)\in {\mathcal {H}}$. Note at this point that due to the sign of $V_{N^{1-\kappa }}\ge 0$, we can write

$$\begin{aligned} \pi _{{\mathcal {H}}}V_{N^{1-\kappa }} \pi _{{\mathcal {H}}}=\pi _{{\mathcal {H}}}\sqrt{V_{N^{1-\kappa }}}\sqrt{V_{N^{1-\kappa }}} \pi _{{\mathcal {H}}}=\left( \sqrt{V_{N^{1-\kappa }}} \pi _{{\mathcal {H}}}\right) ^\dagger \sqrt{V_{N^{1-\kappa }}} \pi _{{\mathcal {H}}}, \end{aligned}$$

or equivalently in coordinates

$$\begin{aligned} \left( \pi _{{\mathcal {H}}}V_{N^{1-\kappa }} \pi _{{\mathcal {H}}}\right) _{jk,mn}=\sum _{st}\overline{\left( \sqrt{V_{N^{1-\kappa }}} \pi _{{\mathcal {H}}}\right) _{st,jk}}\left( \sqrt{V_{N^{1-\kappa }}} \pi _{{\mathcal {H}}}\right) _{st,mn}. \end{aligned}$$

Therefore, the block diagonal structure of ${\widetilde{V}}_{N^{1-\kappa }}$ allows by Eq. (16) for the following representation of the operator $H_{N,\kappa }$ in terms of complete squares

$$\begin{aligned} H_{N,\kappa }&=\sum _k |k|^2 c_k^\dagger c_k+\sum _{jk,mn}\Big (\pi _{\mathcal {L}}(V_{N^{1-\kappa }}-V_{N^{1-\kappa }} R V_{N^{1-\kappa }})\pi _{\mathcal {L}}\Big )_{jk,mn}\psi _{j k}^\dagger \psi _{m n}-{\mathcal {R}}\\&\quad +\sum _{st}\left( \sum _{jk}\left( \sqrt{V_{N^{1-\kappa }}} \pi _{{\mathcal {H}}}\right) _{st,jk}\psi _{jk}\right) ^\dagger \left( \sum _{mn}\left( \sqrt{V_{N^{1-\kappa }}} \pi _{{\mathcal {H}}}\right) _{st,mn}\psi _{mn}\right) . \end{aligned}$$

Using the fact that a complete square $A^\dagger A\ge 0$ is always non-negative yields

20 $$\begin{aligned} H_{N,\kappa } \ge \sum _k |k|^2 c_k^\dagger c_k \! + \! \frac{1}{2}\sum _{jk,mn}\Big (\pi _{{\mathcal {L}}} (V_{N^{1-\kappa }}-V_{N^{1-\kappa }} R V_{N^{1-\kappa }})\pi _{{\mathcal {L}}}\Big )_{jk,mn}\psi _{j k}^\dagger \psi _{m n}-{\mathcal {R}}. \end{aligned}$$

Notably, the interaction term on the right hand side of Eq. (20) only involves index pairs (*jk*) and (*mn*) in the set of low momenta ${\mathcal {L}}$. In the following Lemma 3 we are going to separate the essential contribution in the expression on the right hand side of Eq. (20) from error terms ${\mathcal {E}}_1$ and ${\mathcal {E}}_2$. The error terms ${\mathcal {E}}_1$ and ${\mathcal {E}}_2$ are then shown to be small in Sect. 3.1, given the assumption that the number of excited particles

21 $$\begin{aligned} {\mathcal {N}}:=\sum _{k\ne 0}a_k^\dagger a_k \end{aligned}$$

is small compared to the total number of particles *N*. In preparation for Lemma 3, let us define the coefficients $w_k$, $\sigma _k$ and $\mu _k$ as

22 $$\begin{aligned} w_k&:= N(T \! - \! 1)_{(-k)k,0 0}=\frac{N}{2|k|^2}\Big (V_{N^{1-\kappa }} \! - \! V_{N^{1-\kappa }} R V_{N^{1-\kappa }}\Big )_{(-k)k,0 0} \underset{N\rightarrow \infty }{\simeq }\ \frac{4\pi {\mathfrak {a}}N^\kappa }{|k|^2}, \end{aligned}$$

23 $$\begin{aligned} \sigma _k&:= \frac{N}{4}\Big (V_{N^{1-\kappa }} \! - \! V_{N^{1-\kappa }} R V_{N^{1-\kappa }}\Big )_{k 0 ,k0} \! \! + \! \frac{N}{4}\Big (V_{N^{1-\kappa }} \! - \! V_{N^{1-\kappa }} R V_{N^{1-\kappa }}\Big )_{0 k ,k0} \underset{N\rightarrow \infty }{\simeq }\ 4\pi {\mathfrak {a}}N^\kappa , \end{aligned}$$

24 $$\begin{aligned} \mu _k&:= \mathbbm {1}(|k|<K)4\sigma _k-2\sigma _0 \! - \! |k|^2 |w_k|^2 \underset{N, K\rightarrow \infty }{\simeq }8\pi {\mathfrak {a}}N^{\kappa } \! - \! \frac{\left( 4\pi {\mathfrak {a}}N^\kappa \right) ^2}{|k|^2}, \end{aligned}$$

where the asymptotic behavior stated in Eq. (22), Eq. (23) and Eq. (24) is an immediate consequence of Lemma 1.

### Lemma 3

Let $\mu _k$ be the coefficients defined in Eq. (24). Then

25 $$\begin{aligned} H_{N,\kappa }-4\pi {\mathfrak {a}}_{N^{1-\kappa }}N^\kappa (N-1)\ge \sum _k |k|^2 c_k^\dagger c_k + \sum _{k\ne 0} \mu _k a_k^\dagger a_k-{\mathcal {E}}_1-{\mathcal {E}}_2, \end{aligned}$$

where the error terms ${\mathcal {E}}_1$ and ${\mathcal {E}}_2$ are defined as

26 $$\begin{aligned} {\mathcal {E}}_1&:= {\mathcal {R}}-\sum _{k\ne 0}|k|^2 |w_k|^2 a_k^\dagger a_k, \end{aligned}$$

27 $$\begin{aligned} {\mathcal {E}}_2&:= \sum _{0<|k|< K}\frac{4\sigma _k}{N} {\mathcal {N}}a_k^\dagger a_k-\frac{\sigma _0}{N} \left( {\mathcal {N}}^2+{\mathcal {N}}\right) . \end{aligned}$$

### Proof

Using the fact that $\psi _{jk}=a_ja_k$ for pairs $(jk)\in {\mathcal {L}}$ and the definition of $\sigma _k$ in Eq. (23), we can write

$$\begin{aligned}&\frac{1}{2}\sum _{jk,mn}\Big (\pi _{{\mathcal {L}}} (V_{N^{1-\kappa }}-V_{N^{1-\kappa }} R V_{N^{1-\kappa }})\pi _{{\mathcal {L}}}\Big )_{jk,mn}\psi _{j k}^\dagger \psi _{m n}\\&\quad =\frac{1}{2}\sum _{jk,mn}\Big (\pi _{{\mathcal {L}}} (V_{N^{1-\kappa }}-V_{N^{1-\kappa }} R V_{N^{1-\kappa }})\pi _{{\mathcal {L}}}\Big )_{jk,mn}a_k^\dagger a_j^\dagger a_m a_n\\&\quad =\frac{\sigma _0}{N}\, a_0^{2\dagger }a_0^2+ \! \! \sum _{0<|k|< K} \! \!\frac{4\sigma _k}{N}\, a_0^\dagger a_0\, a_k^\dagger a_k. \end{aligned}$$

Utilizing the operator ${\mathcal {N}}$ defined in Eq. (21), which counts the number of excited particles, we further observe that the operator identities

28 $$\begin{aligned} a_0^\dagger a_0&=N-{\mathcal {N}},\nonumber \\ a_0^{2\dagger }a_0^2&=N(N-1)-(2N-1){\mathcal {N}}+{\mathcal {N}}^2 \end{aligned}$$

hold on the *N*-particle Hilbert space $L^2_\textrm{sym} \! \left( \Lambda ^N\right)$. Hence, we can write

$$\begin{aligned} \frac{\sigma _0}{N} a_0^{2\dagger }a_0^2+ \! \! \! \sum _{0<|k|< K} \frac{4\sigma _k}{N} a_0^\dagger a_0\, a_k^\dagger a_k=4\pi {\mathfrak {a}}_{N^{1-\kappa }}N^\kappa (N-1)+ \! \! \! \sum _{0<|k|< K} 4\sigma _k \, a_k^\dagger a_k-2\sigma _0{\mathcal {N}}-{\mathcal {E}}_2, \end{aligned}$$

where we have used $\sigma _0=4\pi {\mathfrak {a}}_{N^{1-\kappa }}N^\kappa$ by the definition of ${\mathfrak {a}}_{N^{1-\kappa }}$ in Eq. (10). In combination with the lower bound in Eq. (20), we therefore obtain

$$\begin{aligned} H_{N,\kappa }-4\pi {\mathfrak {a}}_{N^{1-\kappa }}N^\kappa (N-1)&\ge \sum _k |k|^2 c_k^\dagger c_k + \sum _{0<|k|< K} 4\sigma _k \, a_k^\dagger a_k-2\sigma _0{\mathcal {N}}-{\mathcal {E}}_2-{\mathcal {R}}\\&= \sum _k |k|^2 c_k^\dagger c_k +\sum _{k\ne 0} \mu _k a_k^\dagger a_k-{\mathcal {E}}_1-{\mathcal {E}}_2 . \end{aligned}$$

$$\square $$

In the following we want to apply the theory of Bogoliubov operators in order to analyze the operator

29 $$\begin{aligned} {\mathbb {X}}:=\sum _k |k|^2 c_k^\dagger c_k + \sum _{k\ne 0} \mu _k a_k^\dagger a_k \end{aligned}$$

appearing on the right hand side of Eq. (25). Note however that the expression in Eq. (29) is not written in the form of a Bogoliubov operator, as it involves two families of operators $a_k$ and $c_k$, where the latter does not satisfy the CCR, not even in an approximate sense. Furthermore, both the operators $a_k$ and $c_k$ do not leave $L^2_\textrm{sym} \! \left( \Lambda ^N\right)$ invariant. It is well understood at this point, that the second issue can be avoided by including an appropriate phase factor $a_0^\dagger \frac{1}{\sqrt{a_0 a_0^\dagger }}$ such that $L^2_\textrm{sym} \! \left( \Lambda ^N\right)$ stays invariant, as has been done in [5, 14, 20]. Regarding the violation of the CCR, we define a new family of operators

30 $$\begin{aligned} d_k:=a_0^\dagger \frac{1}{\sqrt{a_0 a_0^\dagger }}c_k-w_k a_{-k}^\dagger \frac{1}{\sqrt{a_0 a_0^\dagger }} a_0 , \end{aligned}$$

as well as the increment

31 $$\begin{aligned} \delta _k:=a_0^\dagger \frac{1}{\sqrt{a_0a_0^\dagger }}a_k-d_k. \end{aligned}$$

As we will see in Lemma 4, the increments $\delta _k$ can be regarded as being small and therefore the operators $d_k$ do satisfy approximate CCR. In terms of $d_k$ and $\delta _k$ we can express

32 $$\begin{aligned} a_k^\dagger a_k&= a_k^\dagger \frac{1}{\sqrt{a_0a_0^\dagger }}a_0 a_0^\dagger \frac{1}{\sqrt{a_0a_0^\dagger }}a_k=\left( d_k+\delta _k\right) ^\dagger \left( d_k+\delta _k\right) , \end{aligned}$$

33 $$\begin{aligned} c_k^\dagger c_k&= \! c_k^\dagger \frac{1}{\sqrt{a_0a_0^\dagger }}a_0 a_0^\dagger \frac{1}{\sqrt{a_0 a_0^\dagger }}c_k \! = \! \left( d_k \! + \! w_k d_{-k}^\dagger \! + \! w_k\delta _{-k}^\dagger \right) ^\dagger \! \left( d_k \! + \! w_k d_{-k}^\dagger \! + \! w_k\delta _{-k}^\dagger \right) . \end{aligned}$$

Utilizing Eq. (32) and Eq. (33) and defining the coefficients

$$\begin{aligned} A_k&:= |k|^2+|k|^2 |w_k|^2+\mu _k-\epsilon \underset{N,K\rightarrow \infty }{\simeq }\ |k|^2+8\pi {\mathfrak {a}}N^\kappa -\epsilon ,\\ B_k&:= 2|k|^2 w_k\underset{N\rightarrow \infty }{\simeq }\ 8\pi {\mathfrak {a}}N^\kappa ,\\ C_k&:= 2|k|^2 |w_k|^2 \underset{N \rightarrow \infty }{\simeq } \frac{\left( 8\pi {\mathfrak {a}}N^\kappa \right) ^2}{2|k|^2}, \end{aligned}$$

we can write the term in Eq. (29) for $\epsilon \ge 0$ as an (approximate) Bogoliubov operator

34 $$\begin{aligned} {\mathbb {X}} \! - \! \epsilon {\mathcal {N}}&= \sum _k |k|^2 \left( d_k \! + \! w_k d_{-k}^\dagger \right) ^\dagger \! \! \left( d_k \! + \! w_k d_{-k}^\dagger \right) \! + \! \sum _{k\ne 0}(\mu _k \! - \! \epsilon )d_k^\dagger d_k \! - \! {\mathcal {E}}_3\nonumber \\  &=\frac{1}{2} \sum _{k\ne 0} \! \left\{ A_k\left( d_k^\dagger d_k \! + \! d_{-k}^\dagger d_{-k}\right) \! + \! B_k\left( d_k d_{-k} \! + \! d_{-k}^\dagger d_k^\dagger \right) \! \right. \nonumber \\  &\quad \left. + C_k \frac{[d_k,d_k^\dagger ] \! + \! [d_{-k},d_{-k}^\dagger ]}{2}\right\} \! - \! {\mathcal {E}}_3, \end{aligned}$$

where we collect all terms involving an increment $\delta _k$ in the error term ${\mathcal {E}}_3$ defined as

35 $$\begin{aligned} {\mathcal {E}}_3:=\sum _{k\ne 0}(\epsilon \! - \! \mu _k) \! \! \left( \delta _k^\dagger \delta _k \! + \! d_k^\dagger \delta _k \! + \! \mathrm {H.c.} \! \right) \! - \! \sum _k |k|^2 \! \left( |w_k|^2 \delta _k \delta _k^\dagger \! + \! w_k \left( d_k \! + \! w_k d_{-k}^\dagger \right) ^\dagger \delta _{-k}^\dagger \! + \! \mathrm {H.c.} \! \right) . \end{aligned}$$

Note that the asymptotics stated in the definition of $A_k, B_k$ and $C_k$ above Eq. (34) is a direct consequence of Lemma 1. Combining Eq. (34) with the lower bound in Lemma 3 we obtain the following Corollary 1.

### Corollary 1

There exist $N_0,\epsilon _0$ and $K_0$, such that for $N\ge N_0$, $K\ge N^{\frac{\kappa }{2}}$ and $\epsilon < \epsilon _0$

36 $$\begin{aligned} H_{N,\kappa } - 4\pi {\mathfrak {a}}_{N^{1-\kappa }}N^\kappa (N-1)- \frac{1}{2}\sum _{k\ne 0}\left\{ \sqrt{A_k^2-B_k^2}-A_k+C_k\right\} \ge \epsilon {\mathcal {N}}-\sum _{i=1}^4 {\mathcal {E}}_i, \end{aligned}$$

where ${\mathcal {E}}_1$ and ${\mathcal {E}}_2$ are defined in Eq. (26) and Eq. (27), ${\mathcal {E}}_3$ is defined in Eq. (35) and

$$\begin{aligned} {\mathcal {E}}_4:=\frac{1}{2}\sum _{k\ne 0}\left\{ \sqrt{A_k^2-B_k^2}-A_k+C_k\right\} \left( 1-\frac{[d_k,d_k^\dagger ]+[d_{-k},d_{-k}^\dagger ]}{2}\right) . \end{aligned}$$

Before we come to the proof of Corollary 1, we want to emphasize that the constant on the left hand side of Eq. (36) contains the correct leading order energy

$$\begin{aligned} 4\pi {\mathfrak {a}}_{N^{1-\kappa }}N^\kappa (N-1), \end{aligned}$$

and as $N,K\rightarrow \infty$ and $\epsilon \rightarrow 0$, it contains the sub-leading Lee–Huang–Yang correction

$$\begin{aligned} \frac{1}{2} \! \sum _{k\ne 0} \! \left\{ \! \sqrt{|k|^4 \! + \! 16\pi {\mathfrak {a}} N^{\kappa }|k|^2} \! - \! |k|^2 \! - \! 8\pi {\mathfrak {a}} N^{\kappa } \! + \! \frac{(8\pi {\mathfrak {a}} N^{\kappa })^2}{2|k|^2} \! \right\} \end{aligned}$$

as well, which follows from the asymptotic behavior of the coefficients $A_k$, $B_k$ and $C_k$ stated in the definition above Eq. (34). It will be the content of the following Sect. 3.1 to derive sufficient bounds on the error terms ${\mathcal {E}}_i$ appearing on the right side of Eq. (36).

### Proof of Corollary 1

Making use of Lemma 3 and Eq. (34) we obtain the lower bound

$$\begin{aligned}&H_{N,\kappa }-4\pi {\mathfrak {a}}_{N^{1-\kappa }}N^\kappa (N-1)\\&\quad \! \ge \! \epsilon {\mathcal {N}} \! + \! \frac{1}{2} \sum _{k\ne 0} \! \! \left\{ A_k\left( d_k^\dagger d_k \! + \! d_{-k}^\dagger d_{-k}\right) \! + \! B_k\left( d_k d_{-k} \! + \! d_{-k}^\dagger d_k^\dagger \right) \! + \! C_k \frac{[d_k,d_k^\dagger ] \! + \! [d_{-k},d_{-k}^\dagger ]}{2}\right\} \!\\  &\quad - \! \sum _{j=1}^3 {\mathcal {E}}_i. \end{aligned}$$

By Lemma 1 we have $A_k\ge B_k$ for $N\ge N_0$, $K\ge N^{\frac{\kappa }{2}}K_0$ and $\epsilon <\epsilon _0$, and suitable constants $N_0,K_0$ and $\epsilon _0$. Consequently, we can apply Bogoliubov’s Lemma, see [16, 19], which yields

$$\begin{aligned}&\frac{1}{2}\sum _{k\ne 0} \left\{ A_k\left( d_k^\dagger d_k+d_{-k}^\dagger d_{-k}\right) +B_k\left( d_k d_{-k}+d_{-k}^\dagger d_k^\dagger \right) +C_k \frac{[d_k,d_k^\dagger ]+[d_{-k},d_{-k}^\dagger ]}{2}\right\} \\&\quad \ge \frac{1}{2}\sum _{k\ne 0}\left\{ \sqrt{A_k^2-B_k^2}-A_k+C_k\right\} \frac{[d_k,d_k^\dagger ]+[d_{-k},d_{-k}^\dagger ]}{2}\\&\quad = \frac{1}{2}\sum _{k\ne 0}\left\{ \sqrt{A_k^2-B_k^2}-A_k+C_k\right\} -{\mathcal {E}}_4. \end{aligned}$$

$$\square $$

### Proof of the lower bound in Theorem 1

In order to verify the lower bound in Theorem 1, we need sufficient bounds on the error terms ${\mathcal {E}}_i$. For this purpose, we first derive estimates on the increment $\delta _k$ defined in Eq. (31) in the subsequent Lemma 4, which we will use in Lemma 5 in order to control ${\mathcal {E}}_i$. For the rest of this subsection we will always assume that we are in the Gross–Pitaevskii Regime, i.e. we assume $\kappa =0$. Furthermore, recall that we are working on the *N*-particle Hilbert space $L^2_\textrm{sym} \! \left( \Lambda ^N\right)$ and the claimed estimates hold restricted to this space.

#### Lemma 4

There exists a constant $C>0$, with a uniform dependence on *V* in the sense of Eq. (12), such that

$$\begin{aligned} \sum _{k\ne 0}\left( \delta _k^\dagger \delta _k+\delta _k \delta _k^\dagger \right) \le \frac{C}{N-{\mathcal {N}}}({\mathcal {N}}+1)^2. \end{aligned}$$

#### Proof

Let us define $h(x):=\frac{1}{\sqrt{1+\frac{1}{N}-x}}-\sqrt{1-x}$ as well as the coefficients

37 $$\begin{aligned} f_{\ell ,k}&:= \left( \left( T-1\right) _{(\ell -k)k, \ell 0}+\left( T-1\right) _{(\ell -k)k, 0 \ell }\right) \nonumber \\&=\frac{1}{|k|^2 \! + \! |\ell \! - \! k|^2}\left\{ \Big (V_{N^{1-\kappa }} \! - \! V_{N^{1-\kappa }} R V_{N^{1-\kappa }}\Big )_{(\ell -k)k, \ell 0} \! \right. \nonumber \\  &\quad \left. + \! \Big (V_{N^{1-\kappa }} \! - \! V_{N^{1-\kappa }} R V_{N^{1-\kappa }}\Big )_{(\ell -k)k, 0 \ell }\right\} , \end{aligned}$$

in case $\ell \ne 0$, and $f_{\ell ,k}:=0$ otherwise. Then we can write $\delta _k=-\delta _k'+\delta _k''$ with

$$\begin{aligned} \delta _k'&:= \sqrt{a_0^\dagger a_0}\sum _{|\ell |<K}f_{\ell ,k}\, a^{\dagger }_{\ell -k}a_\ell ,\\ \delta _k''&:= w_k\left( \frac{1}{\sqrt{a_0a_0^\dagger }}a_0-\frac{\sqrt{a_0^\dagger a_0}a_0}{N}\right) a_{-k}^\dagger =w_k a_{-k}^\dagger \frac{a_0}{\sqrt{N}} h\!\left( \frac{{\mathcal {N}}}{N}\right) , \end{aligned}$$

where $w_k$ is defined in Eq. (22). Note that $|h(x)|\lesssim \frac{x+\frac{1}{N}}{\sqrt{1-x}}$ and $|w_k|\lesssim \frac{1}{|k|^2}$, and consequently

$$\begin{aligned} \sum _{k\ne 0} (\delta _k'')^\dagger \delta _k''\lesssim \sum _{k\ne 0}h\!\left( \frac{{\mathcal {N}}}{N}\right) \frac{a_{-k}a_{-k}^\dagger }{|k|^4} h\!\left( \frac{{\mathcal {N}}}{N}\right) \! \lesssim \! h\!\left( \frac{{\mathcal {N}}}{N}\right) ^2 \! \! ({\mathcal {N}} \! + \! 1) \! \lesssim \! \frac{({\mathcal {N}}+1)^3}{N(N \! - \! {\mathcal {N}})}, \end{aligned}$$

where we have used $\sum _{k\ne 0}\frac{1}{|k|^4}<\infty$ and the fact that $\left( \frac{a_0}{\sqrt{N}}\right) ^\dagger \frac{a_0}{\sqrt{N}}\le 1$. In a similar fashion, one can show the estimate

$$\begin{aligned} \sum _{k\ne 0} \delta _k''(\delta _k'')^\dagger \lesssim \frac{({\mathcal {N}}+1)^3}{N(N \! - \! {\mathcal {N}})}. \end{aligned}$$

Regarding the estimates on $\delta '_k$, note that $|f_{\ell ,k}|\le \frac{D }{N |k|^2}$ for a suitable constant *D* by Lemma 1 and $a_0^\dagger a_0\le N$, and therefore we obtain by the Cauchy-Schwarz inequality for $\epsilon _k>0$

$$\begin{aligned} \pm 2\mathfrak {Re}\delta _k'\le \sum _{|\ell |\le K}\left( \epsilon _k^{-1} |f_{\ell ,k}|^2a_0^\dagger a_0\, a_{\ell -k}^\dagger a_{\ell -k}+\epsilon _k\, a_\ell ^\dagger a_\ell \right)&\le \frac{D^2}{N|k|^4 \epsilon _k}{\mathcal {N}}+\epsilon _k {\mathcal {N}}\\  &\le \frac{2D}{\sqrt{N}|k|^2}{\mathcal {N}}, \end{aligned}$$

where we have used the optimal choice $\epsilon _k:=\frac{D}{\sqrt{N}|k|^2}$ in the last estimate. Analogously

$$\begin{aligned} \pm \mathfrak {Im}\delta _k'\le \frac{D }{\sqrt{N} |k|^2}{\mathcal {N}} . \end{aligned}$$

Since ${\mathcal {N}}$ commutes with both $\mathfrak {Re}\delta _k'$ and $\mathfrak {Im}\delta _k'$ we can square these inequalities and obtain

$$\begin{aligned} \left( \delta '_k\right) ^\dagger \delta '_k+\delta '_k \left( \delta '_k\right) ^\dagger \le 4\left( \mathfrak {Re}\delta _k'\right) ^2+4\left( \mathfrak {Im}\delta _k'\right) ^2\le \frac{8D^2}{N|k|^4}{\mathcal {N}}^2. \end{aligned}$$

$$\square $$

In the following let ${\mathcal {F}}^+_M\subset L^2_\textrm{sym} \! \left( \Lambda ^N\right)$ denote the spectral subspace ${\mathcal {N}}\le M$, and let us define for an operator *X* the restricted operator $X\Big |_{{\mathcal {F}}^+_M}$ as

$$\begin{aligned} X\Big |_{{\mathcal {F}}^+_M}:=\pi _{{\mathcal {F}}^+_M} X \pi _{{\mathcal {F}}^+_M}, \end{aligned}$$

where $\pi _{{\mathcal {F}}^+_M}$ is the orthogonal projection onto ${\mathcal {F}}^+_M$.

#### Lemma 5

For all $K<\infty$ and $0<r<1$, there exists a constant $C_{K,r}>0$ such that

$$\begin{aligned} \pm {\mathcal {E}}_i\Big |_{{\mathcal {F}}^+_M}\le C_{K,r} \sqrt{\frac{M+1}{N}}({\mathcal {N}}+1). \end{aligned}$$

for all $M\le \min \{rN,N-1\}$ and $i\in \{1,2,3,4\}$. Furthermore, $C_{K,r}$ depends uniformly on *V* in the sense of Eq. (12).

#### Proof

Using $|\sigma _k|\lesssim 1$, see Lemma 1, we immediately obtain

$$\begin{aligned} \pm {\mathcal {E}}_2\le C N^{-1}({\mathcal {N}}+1)^2, \end{aligned}$$

which concludes the case $i=2$. Regarding the case $i=3$, we have by the Cauchy-Schwarz inequality and Lemma 4

38 $$\begin{aligned} \pm {\mathcal {E}}_3 \! \le \! \epsilon \sum _{k\ne 0}\left\{ a_k^\dagger a_k \! + \! c_k^\dagger c_k \! + \! d_k^\dagger d_k \! + \! (d_k \! + \! w_k d_{-k}^\dagger )^\dagger (d_k \! + \! w_k d_{-k}^\dagger )\right\} \! + \! \epsilon ^{-1} \frac{C}{N \! - \! {\mathcal {N}}}({\mathcal {N}}+1)^2 \end{aligned}$$

for any $\epsilon >0$. Writing $d_k$ defined in Eq. (30) as $d_k=\frac{1}{\sqrt{a_0^\dagger a_0}}a_0^\dagger a_k-\delta _k$, we obtain by Lemma 4

39 $$\begin{aligned} \sum _{k\ne 0}d_k^\dagger d_k\le 2\sum _{k\ne 0}a_k^\dagger a_k + 2\sum _{k\ne 0}\delta _k^\dagger \delta _k\lesssim \left( 1-\frac{{\mathcal {N}}}{N}\right) ^{-1}\big ({\mathcal {N}}+1\big ). \end{aligned}$$

Furthermore, we have the estimate

40 $$\begin{aligned} \sum _{k\ne 0}c_k^\dagger c_k&=\sum _{k\ne 0}\left( d_k+w_k a_{-k}^\dagger a_0\frac{1}{\sqrt{a_0^\dagger a_0}}\right) ^\dagger \left( d_k+w_k a_{-k}^\dagger a_0\frac{1}{\sqrt{a_0^\dagger a_0}}\right) \nonumber \\&\le 2\sum _{k\ne 0} d_k^\dagger d_k + \sum _{k\ne 0}|w_k|^2 a_k a_k^\dagger \nonumber \\&\lesssim \left( 1-\frac{{\mathcal {N}}}{N}\right) ^{-1}\big ({\mathcal {N}}+1\big ), \end{aligned}$$

and in a similar fashion

41 $$\begin{aligned} \sum _{k\ne 0}(d_k+w_k d_{-k}^\dagger )^\dagger (d_k+w_k d_{-k}^\dagger )\lesssim \left( 1-\frac{{\mathcal {N}}}{N}\right) ^{-1} \big ({\mathcal {N}}+1\big ). \end{aligned}$$

Combining Eq. (38), Eq. (39), Eq. (40) and Eq. (41) yields

$$\begin{aligned} \pm {\mathcal {E}}_3\Big |_{{\mathcal {F}}^+_M}\lesssim \epsilon \left( 1-\frac{M}{N}\right) ^{-1} \! \! \big ({\mathcal {N}}+1\big )+\epsilon ^{-1} \frac{M+1}{N-M}\big ({\mathcal {N}}+1\big ), \end{aligned}$$

which concludes the proof of the case $i=3$ for the optimal choice $\epsilon :=\sqrt{\frac{M+1}{N-M}}$. Regarding the case $i=4$, we observe that $a_0^\dagger \frac{1}{a_0 a_0^\dagger }a_0 \Psi =\Psi$ for $\Psi \in {\mathcal {F}}^+_{N-1}$ and hence

42 $$\begin{aligned} \left[ a_0^\dagger \frac{1}{\sqrt{a_0 a_0^\dagger }}a_k,a_k^\dagger \frac{1}{\sqrt{a_0 a_0^\dagger }}a_0\right] \Bigg |_{{\mathcal {F}}^+_{N-1}}=1|_{{\mathcal {F}}^+_{N-1}}. \end{aligned}$$

Note that the restriction to ${\mathcal {F}}^+_{N-1}$ is necessary for Eq. (42) to hold, e.g. the state $\Psi :=(a_j^\dagger )^N \Omega$, where $\Omega$ is the vacuum and $j\notin \{0,k\}$, satisfies $a_0\Psi =0$ and $a_k\Psi =0$, and as a consequence

$$\begin{aligned} \left[ a_0^\dagger \frac{1}{\sqrt{a_0 a_0^\dagger }}a_k,a_k^\dagger \frac{1}{\sqrt{a_0 a_0^\dagger }}a_0\right] \Psi =0\ne \Psi . \end{aligned}$$

Using Eq. (42), we can write ${\mathcal {E}}_4|_{{\mathcal {F}}^+_{N-1}}$ as

$$\begin{aligned} \frac{1}{2}\sum _{k\ne 0}\left\{ A_k-\sqrt{A_k^2-B_k^2}-C_k\right\} \left( \left[ \delta _k,a_k^\dagger \frac{1}{\sqrt{a_0 a_0^\dagger }}a_0\right] +\left[ a_0^\dagger \frac{1}{\sqrt{a_0 a_0^\dagger }}a_k,\delta _k^\dagger \right] +\left[ \delta _k,\delta _k^\dagger \right] \right) \Bigg |_{{\mathcal {F}}^+_{N-1}}. \end{aligned}$$

Multiplying out the commutators and estimating the resulting products in the same way as we did in the case $i=3$, concludes the proof of the case $i=4$. Regarding the final case $i=1$, let us write

$$\begin{aligned} {\mathcal {E}}_1&=\sum _{(\ell ,\ell ')\in A_K}{\mathcal {G}}^{\ell ,\ell '}+\sum _{k\ne 0}|k|^2 N^{-2}|w_k|^2 a_0^{2\dagger }a_0^2 a_k^\dagger a_k-\sum _{k\ne 0}|k|^2|w_k|^2 a_k^\dagger a_k\\&=\frac{1}{2}\sum _{(\ell ,\ell ')\in A_K}\left( {\mathcal {G}}^{\ell ,\ell '}+\mathrm {H.c.}\right) \\&\quad +\sum _{k\ne 0}|k|^2 |w_k|^2 N^{-2}\left( a_0^{2\dagger }a_0^2-N^2\right) a_k^\dagger a_k, \end{aligned}$$

where we define the set $A_K\subseteq 2\pi {\mathbb {Z}}^6$ and the operators ${\mathcal {G}}^{\ell ,\ell '}$ as

$$\begin{aligned} A_K&:= \{(\ell ,\ell ')\ne 0: |\ell |,|\ell '|<K\} ,\\ {\mathcal {G}}^{\ell ,\ell '}&:= \sum _{k\ne 0}|k|^2 \overline{f_{\ell ,k}}f_{\ell ',k} a_{\ell '-k}^\dagger a_{\ell }^\dagger a_0^\dagger a_{\ell '} a_0 a_{\ell -k} , \end{aligned}$$

and $f_{\ell ,k}$ is defined in Eq. (37). Note that $\left| |k|^2\overline{f_{\ell ,k}}f_{\ell ',k}\right| \lesssim N^{-2}$, see for example the proof of Lemma 4, and we have for $(\ell ,\ell ')\ne (0,0)$ the estimate

$$\begin{aligned} \left\| \left( a_{\ell '}^\dagger a_0^\dagger a_\ell a_0\right) \big |_{{\mathcal {F}}^+_M} \right\| \le \sqrt{M}N^{\frac{3}{2}}. \end{aligned}$$

Consequently, we have by the Cauchy-Schwarz inequality

$$\begin{aligned} \pm \left( {\mathcal {G}}^{\ell ,\ell '}+\mathrm {H.c.}\right) \big |_{{\mathcal {F}}^+_M}\lesssim \sqrt{\frac{M}{N}}\sum _{k\ne 0}\left( a^\dagger _{\ell '-k}a_{\ell '-k}+a_{\ell -k}^\dagger a_{\ell -k}\right) \le 2\sqrt{\frac{M}{N}}{\mathcal {N}}. \end{aligned}$$

This concludes the proof, since the set $A_K$ is finite and we have by Eq. (28)

$$\begin{aligned} \pm \left( \sum _{k\ne 0}|k|^2 |w_k|^2 N^{-2}\left( a_0^{2\dagger }a_0^2-N^2 \right) a_k^\dagger a_k\right) \bigg |_{{\mathcal {F}}^+_M}\lesssim \frac{M+1}{N}{\mathcal {N}}. \end{aligned}$$

$$\square $$

With Lemma 5 at hand, we are now in a position to verify the lower bound in Theorem 1. In the following let $\Psi _N$ be the ground state of the operator $H_N$ and let $f,g:{\mathbb {R}}\longrightarrow [0,1]$ be smooth functions satisfying $f^2+g^2=1$ and $f(x)=1$ for $x\le \frac{1}{2}$ as well as $f(x)=0$ for $x\ge 1$. Then we define for $0<\rho <1$ the truncated states

$$\begin{aligned} \Theta _N:=\left\| f \! \left( \frac{{\mathcal {N}}}{\rho N}\right) \! \Psi _N \right\| ^{-1} f \! \left( \frac{{\mathcal {N}}}{\rho N}\right) \! \Psi _N. \end{aligned}$$

Note that the ground state $\Psi _N$ satisfies complete Bose-Einstein condensation

$$\begin{aligned} \frac{1}{N}{\langle {\Psi _{N},{\mathcal {N}}\Psi _{N}}\rangle }\underset{N\rightarrow \infty }{\longrightarrow }0, \end{aligned}$$

according to the well known results in [15]. Consequently, we obtain by the IMS inequality, which can for example be found in [12, Proposition 20], respectively which follows from the methods presented in our more general Lemma 18,

43 $$\begin{aligned} {\langle {\Theta _N,H_N\Theta _N}\rangle }\le E_{N}+\frac{N}{\rho N-2{\langle {\Psi _{N},{\mathcal {N}}\Psi _{N}}\rangle }}\frac{C'}{N}\le E_{N}+\frac{C}{N} \end{aligned}$$

for suitable $C',C$. Since $\Theta _N$ is an element of ${\mathcal {F}}^+_{\rho N}$, we have by Lemma 5

44 $$\begin{aligned} \left\langle \Theta _N,\left( \epsilon {\mathcal {N}}-\sum _{i=1}^4 {\mathcal {E}}_i\right) \Theta _N\right\rangle&\ge \left( \epsilon -4C_{K,\frac{1}{2}}\sqrt{\rho +\frac{1}{N}}\right) {\langle {\Theta _N,{\mathcal {N}}\Theta _N}\rangle } -4C_{K,\frac{1}{2}}\sqrt{\rho +\frac{1}{N}}\nonumber \\&\ge -4C_{K,\frac{1}{2}}\sqrt{\rho +\frac{1}{N}}. \end{aligned}$$

for $\epsilon >0$, $K<\infty$ and $0<\rho <\frac{1}{2}$ small enough such that $\epsilon -4C_{K,\frac{1}{2}}\sqrt{\rho +\frac{1}{N}}\ge 0$ for *N* large enough. Assuming that *N* and *K* are large enough and $\epsilon$ small enough such that the assumptions of Corollary 1 hold, we obtain by Eq. (36) together with Eq. (43) and Eq. (44)

$$\begin{aligned} E_N \! - \! 4\pi {\mathfrak {a}}_N (N \! - \! 1)&\ge {\langle {\Theta _N,H_N\Theta _N}\rangle } - 4\pi {\mathfrak {a}}_N (N - 1)-\frac{C}{N}\\&\ge \frac{1}{2}\sum _{k\ne 0}\left\{ \sqrt{A_k^2-B_k^2}-A_k+C_k\right\} \\  &\quad + \left\langle \Theta _N,\left( \epsilon {\mathcal {N}}-\sum _{i=1}^4 {\mathcal {E}}_i\right) \Theta _N\right\rangle -\frac{C}{N}\\&\ge \frac{1}{2}\sum _{k\ne 0}\left\{ \sqrt{A_k^2-B_k^2}-A_k+C_k\right\} -4C_{K,\frac{1}{2}}\sqrt{\rho +\frac{1}{N}}-\frac{C}{N}. \end{aligned}$$

Since $|\sqrt{A_k^2-B_k^2}-A_k+C_k|\lesssim \frac{1}{|k|^4}$ by Lemma 1, we conclude using dominated convergence

$$\begin{aligned}&\lim _{\epsilon \rightarrow 0,K\rightarrow \infty } \lim _{\rho \rightarrow 0}\lim _{N\rightarrow \infty } \left[ \sum _{k\ne 0}\left\{ \sqrt{A_k^2-B_k^2}-A_k+C_k\right\} -4C_{K,\frac{1}{2}}\sqrt{\rho +\frac{1}{N}}-\frac{C}{N}\right] \\&\quad \ =\sum _{k\ne 0}\left\{ \sqrt{|k|^4+16\pi {\mathfrak {a}} |k|^2}-|k|^2-8\pi {\mathfrak {a}}+\frac{(8\pi {\mathfrak {a}})^2}{2|k|^2}\right\} . \end{aligned}$$

While the upper bound in Theorem 1 follows immediately from Theorem 3 proven in Sect. 5, we want to emphasize that way less careful estimates are sufficient to obtain an error term of the order $o_{N\rightarrow \infty }(1)$ compared to the claimed error term $O_{N\rightarrow \infty }\! \left( \frac{\log N}{N}\right)$ in Theorem 3.

## Beyond the Gross–Pitaevskii Regime

In this section we will explain how to treat the case $\kappa >0$. For this purpose we use the following algebraic version of Bogoliubov’s Lemma, see for example [16, 20],

$$\begin{aligned}&\frac{1}{2}\sum _{k\ne 0} \left\{ A_k\left( d_k^\dagger d_k+d_{-k}^\dagger d_{-k}\right) +B_k\left( d_k d_{-k}+d_{-k}^\dagger d_k^\dagger \right) +C_k \frac{[d_k,d_k^\dagger ]+[d_{-k},d_{-k}^\dagger ]}{2}\right\} \\&\quad = \sum _{k\ne 0}e_k\left( \gamma _k d_k \! + \! \nu _k d_{-k}^\dagger \right) ^\dagger \left( \gamma _k d_k \! + \! \nu _k d_{-k}^\dagger \right) \! \\  &\quad + \! \frac{1}{2}\sum _{k\ne 0}\left\{ \sqrt{A_k^2 \! - \! B_k^2} \! - \! A_k+C_k\right\} \frac{[d_k,d_k^\dagger ] \! + \! [d_{-k},d_{-k}^\dagger ]}{2}, \end{aligned}$$

where we define the coefficients

$$\begin{aligned} e_k&:= \sqrt{(A_k)^2-(B_k)^2},\\ \alpha _k&:= \frac{1}{B_k}\left( A_k-\sqrt{A_k^2-B_k^2}\right) ,\\ \gamma _k&:= \frac{1}{\sqrt{1-\alpha _k^2}},\\ \nu _k&:= \frac{\alpha _k}{\sqrt{1-\alpha _k^2}}. \end{aligned}$$

Note that by Lemma 1, it is easy to see that $0\le \nu _k\le \gamma _k \lesssim \frac{N^{\frac{\kappa }{4}}}{\sqrt{|k|}}$ for $|k|\lesssim N^{\frac{\kappa }{2}}$ and $|\nu _k|\lesssim \frac{N^\kappa }{|k|^2}$ globally. Setting $\epsilon :=0$ and using the estimate in Eq. (25) together with the identity in Eq. (34), we therefore obtain

45 $$\begin{aligned}&H_{N,\kappa } -4\pi {\mathfrak {a}}_{N^{1-\kappa }}N^\kappa (N-1)- \frac{1}{2}\sum _{k\ne 0}\left\{ \sqrt{A_k^2-B_k^2}-A_k+C_k\right\} \nonumber \\&\quad \ge \sum _{k\ne 0}e_k\left( \gamma _k d_k+\nu _k d_{-k}^\dagger \right) ^\dagger \left( \gamma _k d_k+\nu _k d_{-k}^\dagger \right) -\sum _{i=1}^4 {\mathcal {E}}_i. \end{aligned}$$

### Control of the error terms ${\mathcal {E}}_i$

In order to obtain a useful representation of the error terms ${\mathcal {E}}_1$ and ${\mathcal {E}}_3$, recall the definition $f_{\ell ,k}$ from Eq. (37) and let us introduce

$$\begin{aligned} \Lambda _{k \ell , k' \ell '}&:= \delta _{k+\ell =k'+\ell '}|k-\ell '|^2 N\overline{f_{\ell ',\ell '-k}}f_{\ell ,\ell -k'},\\ \Upsilon ^{(1)}_{\ell ,k}&:= 2 N^{\frac{1}{2}} |k|^2\overline{f_{\ell ,-k}}w_k,\\ \Upsilon ^{(2)}_{\ell ,k}&:= -N^{\frac{1}{2}}|k|^2 \overline{w_k} f_{\ell ,-k},\\ \Upsilon ^{(3)}_{\ell ,k}&:= -N^{\frac{1}{2}}\left( \mathbbm {1}(|k|<K)\frac{4\sigma _k}{N}-\frac{2\sigma _0}{N}\right) f_{\ell +k,k}. \end{aligned}$$

Furthermore, we define the operators

$$\begin{aligned} O_1&:= N^{-\frac{3}{2}}a_0^\dagger a_0^2 ,\\ O_2&:= O_3:=\frac{1}{\sqrt{a_0 a_0^\dagger }}\frac{a_0}{\sqrt{N}} \sqrt{a_0 a_0^\dagger }. \end{aligned}$$

Using the decomposition $\delta _k=-\delta _k'+\delta _k''$ from the proof of Lemma 4, we can then write

46 $$\begin{aligned} {\mathcal {E}}_1&=\sum _{k \ell , k' \ell '}\Lambda _{k \ell , k' \ell '}\, a_{\ell '}^\dagger a_{k'}^\dagger \frac{a_0^\dagger a_0}{N} a_k a_\ell +\left( \sum _{\ell ,k}\Upsilon ^{(1)}_{\ell ,k} O_1\, a_k^\dagger a_\ell ^\dagger a_{k+\ell }+\mathrm {H.c.}\right) \nonumber \\&\quad +\sum _{k\ne 0}|k|^2 |w_k|^2 N^{-2}\left( a_0^{2\dagger }a_0^2-N^2\right) a_k^\dagger a_k, \end{aligned}$$

47 $$\begin{aligned} {\mathcal {E}}_3&=\left( \sum _{\ell ,k}\Upsilon ^{(2)}_{\ell ,k}O_2\, a_k^\dagger a_\ell ^\dagger a_{k+\ell }+\mathrm {H.c.}\right) +\left( \sum _{\ell ,k}\Upsilon ^{(3)}_{\ell ,k}O_3\, a_k^\dagger a_\ell ^\dagger a_{k+\ell }+\mathrm {H.c.}\right) \nonumber \\&\quad +\sum _k |k|^2 |w_k|^2\big ([\delta _k,d_k^\dagger ]+[d_k,\delta _k^\dagger ]+[\delta _k,\delta _k^\dagger ]\big )+F_1+F_1^\dagger + F_2, \end{aligned}$$

where we have defined

$$\begin{aligned} F_1&:= -\sum _{k\ne 0}a_k^\dagger \frac{1}{\sqrt{a_0 a_0^\dagger }}a_0 \left( N\left( \mathbbm {1}(|k|<K)\frac{4\sigma _k}{N}-\frac{2\sigma _0}{N}\right) \delta ''_k+|k|^2 w_k(\delta ''_{-k})^\dagger \right) ,\\ F_2&:= \sum _k |k|^2 w_k \left( \delta _k^\dagger \delta _{-k}^\dagger +\delta _{-k}\delta _k\right) . \end{aligned}$$

This Subsection is devoted to the derivation of suitable bounds on the most prominent error contributions $\sum _{k \ell , k' \ell '}\Lambda _{k \ell , k' \ell '}\, a_{\ell '}^\dagger a_{k'}^\dagger \frac{a_0^\dagger a_0}{N} a_k a_\ell$ and $\sum _{\ell ,k}\Upsilon ^{(i)}_{\ell ,k} O_i\, a_k^\dagger a_\ell ^\dagger a_{k+\ell }$ in Lemma 6 and Lemma 7. The residual error terms are then taken care of in Appendix B. Let us at this point introduce the new operators

$$\begin{aligned} b_k&:= \gamma _k a_0^\dagger \frac{1}{\sqrt{a_0 a_0^\dagger }}a_k+\nu _k a_{-k}^\dagger \frac{1}{\sqrt{a_0 a_0^\dagger }} a_0,\\ \widetilde{{\mathcal {N}}}&:= \sum _{k\ne 0}b_k^\dagger b_k. \end{aligned}$$

Notably, the operators $b_k$ satisfy the CCR on ${\mathcal {F}}^+_{N-1}$, i.e. $\left[ b_k,b_\ell ^\dagger \right] \Psi =\delta _{k,\ell }\Psi$ for $\Psi \in {\mathcal {F}}^+_{N-1}$. Let us furthermore introduce ${\mathcal {F}}^{\le }_{M_0}$ as the spectral subspace $\sum _{0<|k|<K}a_k^\dagger a_k\le M_0$.

#### Lemma 6

For $0<\delta <\frac{1}{5}$, there exists a constant $C>0$ such that we have

48 $$\begin{aligned}&\pm \! \! \! \sum _{k \ell , k' \ell '} \! \! \! \Lambda _{k \ell , k' \ell '}\, a_{\ell '}^\dagger a_{k'}^\dagger \frac{a_0^\dagger a_0}{N} a_k a_\ell \bigg |_{{\mathcal {F}}^{\le }_{M_0}} \! \! \! \! \! \le \! C\! \! \left( \! \! N^{\frac{5\kappa }{2}+3\delta }\frac{M_0 \! + \! 1}{N}+N^{\frac{11\kappa }{2}+3\delta -1} \! \! \right) \! \!\nonumber \\  &\quad \times \left( \! \! \sum _k |k|^{1-2\delta } b_k^\dagger b_k\Big |_{{\mathcal {F}}^{\le }_{M_0}} \! \! \! \! + \! 1 \! \! \right) \! \! , \end{aligned}$$

49 $$\begin{aligned}&\pm \! \! \sum _{k \ell , k' \ell '}\Lambda _{k \ell , k' \ell '}\, a_{\ell '}^\dagger a_{k'}^\dagger \frac{a_0^\dagger a_0}{N} a_k a_\ell \! \le \! C N^{\frac{11\kappa }{2}+3\delta -1} \! \left( \widetilde{{\mathcal {N}}} \! + \! 1\right) \! \! \left( \sum _k |k|^{1-2\delta } b_k^\dagger b_k \! + \! 1\right) \! \! . \end{aligned}$$

Furthermore, *C* depends uniformly on *V* in the sense of Eq. (12).

#### Proof

Using $a_k=\frac{1}{\sqrt{a_0 a_0^\dagger }}a_0\gamma _k b_k+\frac{1}{\sqrt{a_0 a_0^\dagger }}a_0\nu _{-k}b_{-k}^\dagger$ allows us to rewrite

50 $$\begin{aligned} \sum _{k \ell , k' \ell '}\Lambda _{k \ell , k' \ell '}\, a_{\ell '}^\dagger a_{k'}^\dagger \frac{a_0^\dagger a_0}{N} a_k a_\ell&=\sum _{k \ell , k' \ell '}\Lambda _{k \ell , k' \ell '}\, a_{\ell '}^\dagger \left( \gamma _{k'} b_{k'}+\nu _{-k'}b_{-k'}^\dagger \right) ^\dagger \nonumber \\  &\quad \times O \left( \gamma _k b_k+\nu _{-k}b_{-k}^\dagger \right) a_\ell \end{aligned}$$

with $O:=a_0^\dagger \frac{1}{\sqrt{a_0 a_0^\dagger }}\frac{a_0^\dagger a_0}{N}\frac{1}{\sqrt{a_0 a_0^\dagger }}a_0$. In order to establish a good upper bound on the matrix $\Lambda$, let us define the auxiliary matrix

$$\begin{aligned} \Lambda ^{(\delta )}_{k \ell , k' \ell '}:=\Lambda _{k \ell ,k' \ell '}|k|^{\delta -\frac{1}{2}}|k'|^{\delta -\frac{1}{2}}. \end{aligned}$$

Making use of

$$\begin{aligned} |\ell '-k|^2 | f_{\ell ,\ell -k'}| \!&\le \! |(V_{N^{1-\kappa }} \! - \! V_{N^{1-\kappa }}RV_{N^{1-\kappa }})_{k'(\ell -k'),\ell 0} \! \\  &\quad + \! (V_{N^{1-\kappa }} \! - \! V_{N^{1-\kappa }}RV_{N^{1-\kappa }})_{k'(\ell -k'),0 \ell }| \! \lesssim \! N^{\kappa -1} \end{aligned}$$

by Lemma 1, we obtain that $\Lambda ^{(\delta )}$ satisfies the weighted Schur test

51 $$\begin{aligned} \sum _k p(k)\left| \Lambda ^{(\delta )}_{k (k'+\ell '-k),k' \ell '}\right| \le C p(k')N^{\kappa } \sup _{\ell '}\sum _k |k|^{2\delta -1}\left| f_{\ell ',\ell '-k}\right| , \end{aligned}$$

with the weight function $p(k):=|k|^{\delta -\frac{1}{2}}$ for a suitable constant *C* and all $\ell '$ and $k'$. Defining

$$\begin{aligned} h_{\ell '}(k):=|k|^{3\delta +2}|f_{\ell ',\ell '-k}|, \end{aligned}$$

we obtain as a consequence of the weighted Schur Test in Eq. (51)

$$\begin{aligned} \Vert \Lambda ^{(\delta )}\Vert \!&\lesssim \! N^{\kappa }\sup _{\ell '}\!\!\sum _k|k|^{2\delta -1}\!\!\left| f_{\ell ',\ell '-k}\right| \\&= \! N^{\kappa }\sup _{\ell '} \! \sum _k h_{\ell '}(k) |k|^{-(3+\delta )}\\&\lesssim N^{\kappa }\sup _{\ell '} \! \left( \sum _k h_{\ell '}(k)^{\frac{1+\delta }{3\delta }} |k|^{-(3+\delta )}\right) ^{\frac{3\delta }{1+\delta }}\\&=N^{2\kappa -1}\sup _{\ell '}\! \left( \sum _k |k|^{-2}\left( N^{1-\kappa }|k|^2 f_{\ell ',\ell '-k}\right) ^{\frac{1+\delta }{3\delta }}\right) ^{\frac{3\delta }{1+\delta }}, \end{aligned}$$

where we have used that there is an embedding of $L^{1}$ in $L^{\frac{1+\delta }{3\delta }}$ for the finite measure with discrete density $|k|^{-(3+\delta )}$. By Lemma 1 we know that $N^{1-\kappa }|k|^2 f_{\ell ',\ell '-k}\lesssim 1$, which yields together with the assumption $\delta <\frac{1}{5}$, or equivalently $\frac{1+\delta }{3\delta }>2$, the estimate

$$\begin{aligned} \sum _k |k|^{-2}\left( N^{-\kappa }|k|^2 f_{\ell ',\ell '-k}\right) ^{\frac{1+\delta }{3\delta }} \! \lesssim \! N^{2-2\kappa }\sum _k |k|^{2}(f_{\ell ',\ell '-k})^2 \! \lesssim N^{1-\kappa }\le N, \end{aligned}$$

where we have used Lemma 14. Therefore, $\Vert \Lambda ^{(\delta )}\Vert \lesssim N^{2\kappa +3\delta -1}$, or equivalently we have $\pm \Lambda \lesssim N^{2\kappa +3\delta -1}(-\Delta _x)^{\frac{1}{2}-\delta }$. Consequently, we obtain

$$\begin{aligned}&\pm \! \sum _{k \ell , k' \ell '}\Lambda _{k \ell , k' \ell '}\, a_{\ell '}^\dagger a_{k'}^\dagger \frac{a_0^\dagger a_0}{N} a_k a_\ell \\&\quad \lesssim \! N^{2\kappa +3\delta -1} \! \! \! \! \! \sum _{ 0<|\ell |< K}\sum _{k\ne 0}|k|^{1-2\delta } a_{\ell }^\dagger \left( \gamma _{k} b_{k} \! + \! \nu _{-k}b_{-k}^\dagger \right) ^\dagger \! \! \left( \gamma _k b_k \! + \! \nu _{-k}b_{-k}^\dagger \right) \! a_\ell \\&\quad \lesssim N^{2\kappa +3\delta -1}\sum _{0<|\ell |< K}\sum _{k\ne 0}|k|^{1-2\delta }(\gamma _k^2+\nu _k^2) a_{\ell }^\dagger b_{k}^\dagger b_k a_\ell \\&\qquad +N^{2\kappa +3\delta -1}\left( \sum _{k\ne 0} |k|^{1-2\delta }\nu _k^2\right) \sum _{0<|\ell |< K} a_{\ell }^\dagger a_\ell \end{aligned}$$

by Eq. (50), where we have used $\Vert O\Vert \lesssim 1$ and $[b_k,b_k^\dagger ]\le 1$. Note that

$$\begin{aligned} N^{2\kappa +3\delta -1}\left( \sum _{k\ne 0} |k|^{1-2\delta }\nu _k^2\right) \sum _{0<|\ell |< K} a_{\ell }^\dagger a_\ell&\lesssim N^{4\kappa +3\delta -1}\sum _{0<|\ell |< K} a_{\ell }^\dagger a_\ell \\  &\lesssim N^{\frac{11\kappa }{2}+3\delta -1}\! \left( \widetilde{{\mathcal {N}}}+1\right) . \end{aligned}$$

Furthermore, $\gamma _k^2+\nu _k^2\lesssim N^{\frac{\kappa }{2}}$ and $[ b_k, a_\ell ]=-\delta _{k,-\ell }\frac{1}{\sqrt{a_0 a_0^\dagger }}a_0\nu _{k}$, and therefore

$$\begin{aligned} \sum _{0<|\ell |< K}\sum _{k\ne 0}|k|^{1-2\delta }(\gamma _k^2+\nu _k^2) a_{\ell }^\dagger b_{k}^\dagger b_k a_\ell \!&\lesssim \! N^{\frac{\kappa }{2}}\sum _{k\ne 0}|k|^{1-2\delta } b_k^\dagger \left( \sum _{0<|\ell |< K}a_\ell ^\dagger a_\ell \! \right) \! b_k \! \\  &\quad + \! N^{\frac{\kappa }{2}}\sum _{k\ne 0}|k|^{1-2\delta }\nu _k^2. \end{aligned}$$

Making use of the fact that

$$\begin{aligned} N^{-1}b_k^\dagger \left( \sum _{0<|\ell |< K}a_\ell ^\dagger a_\ell \! \right) \! b_k\bigg |_{{\mathcal {F}}^{\le }_{M_0}}\le \frac{M_0+1}{N} b_k^\dagger b_k \end{aligned}$$

concludes the proof of Eq. (48), and making use of the fact that

$$\begin{aligned} b_k^\dagger \left( \sum _{0<|\ell |< K}a_\ell ^\dagger a_\ell \! \right) \! b_k\le N^{\frac{3\kappa }{2}}\widetilde{{\mathcal {N}}} b_k^\dagger b_k, \end{aligned}$$

see Lemma 16, yields Eq. (49). $\square$

#### Lemma 7

For $r<1$ and $\kappa \le 1$, there exists a $C>0$, depending uniformly on *V* in the sense of Eq. (12), such that for $i\in \{1,2,3\}$

52 $$\begin{aligned} \pm \! \left( \sum _{\ell ,k}\Upsilon ^{(i)}_{\ell ,k}O_i\, a_k^\dagger a_\ell ^\dagger a_{k+\ell } \! + \! \mathrm {H.c.}\right) \bigg |_{{\mathcal {F}}^{\le }_{M_0}\cap {\mathcal {F}}^+_{rN}} \! \! \! \le \! C N^{\frac{5\kappa }{2}}\sqrt{\frac{M_0 \! + \! 1}{N} \! + \! N^{\frac{3\kappa }{2}-1}}\left( \widetilde{{\mathcal {N}}}\Big |_{{\mathcal {F}}^{\le }_{M_0}\cap {\mathcal {F}}^+_{rN}} \! \! + \! 1 \! \right) \! . \end{aligned}$$

Furthermore, $$\pm \! \left( \sum _{\ell ,k}\Upsilon ^{(i)}_{\ell ,k}O_i\, a_k^\dagger a_\ell ^\dagger a_{k+\ell } \! + \! \mathrm {H.c.}\right) \bigg |_{{\mathcal {F}}^+_{rN}}\lesssim N^{\frac{13\kappa }{4}-\frac{1}{2}}\! \left( \widetilde{{\mathcal {N}}}+1\right) ^2\bigg |_{{\mathcal {F}}^+_{rN}}$$.

#### Proof

Using $a_k=\frac{1}{\sqrt{a_0 a_0^\dagger }}a_0\gamma _k b_k+\frac{1}{\sqrt{a_0 a_0^\dagger }}a_0\nu _{-k}b_{-k}^\dagger$, we can rewrite

$$\begin{aligned} \left( \sum _{\ell ,k}\Upsilon ^{(i)}_{\ell ,k}O_i\, a_k^\dagger a_\ell ^\dagger a_{k+\ell } \! + \! \mathrm {H.c.}\right) \!&= \! \left( \sum _{\ell ,k}X^{(i)}_{\ell ,k}{\widetilde{O}}_i b_k^\dagger a_\ell ^\dagger b_{k+\ell } \! + \! \mathrm {H.c.}\right) \! \\  &\quad + \! \left( \sum _{\ell ,k}Y^{(i)}_{\ell ,k}{\widetilde{O}}_i a_\ell ^\dagger b_{k+\ell }b_{-k} \! + \! \mathrm {H.c.}\right) , \end{aligned}$$

where we define

$$\begin{aligned} X^{(i)}_{\ell ,k}&:= \gamma _k \gamma _{\ell +k}\Upsilon ^{(i)}_{\ell ,k} +\nu _{k}\nu _{k+\ell }\Upsilon ^{(i)}_{\ell ,-(k+\ell )},\\ Y^{(i)}_{\ell ,k}&:= \big (\nu _k \gamma _{k+\ell }+\nu _{k+\ell }\gamma _k\big )\Upsilon ^{(i)}_{\ell ,k},\\ {\widetilde{O}}_i&:= O_i \end{aligned}$$

in the case $i\in \{1,2\}$, and

$$\begin{aligned} X^{(3)}&:= \nu _{-k}\gamma _{k+\ell }\overline{\Upsilon ^{(3)}_{-k,k+\ell }}+\nu _{k+\ell }\gamma _{-k} \overline{\Upsilon ^{(3)}_{k+\ell ,-k}} ,\\ Y^{(3)}_{\ell ,k}&:= \big (\gamma _{-k}\gamma _{k+\ell }+\nu _{-k}\nu _{k+\ell }\big )\overline{\Upsilon ^{(3)}_{-k,\ell +k}},\\ {\widetilde{O}}_3&:= O_3^\dagger \left( \frac{1}{\sqrt{a_0 a_0^\dagger }}a_0\right) ^2. \end{aligned}$$

Using the fact that we can write

$$\begin{aligned} \left( T-1\right) _{(\ell -k)k, \ell 0}=\frac{1}{|k|^2+|\ell -k|^2}\Big (V_{N^{1-\kappa }}-V_{N^{1-\kappa }} R V_{N^{1-\kappa }}\Big )_{(\ell -k)k, \ell 0}, \end{aligned}$$

the bounds from Lemma 1 and the simple observation that $|\gamma _k|,|\nu _k|\lesssim N^{\frac{\kappa }{4}}$, yields the inequalities $|X_{\ell ,k}|,|Y_{\ell ,k}|\lesssim N^{\frac{5\kappa -1}{2}}\frac{1}{|\ell |^2+|k|^2}$. Consequently

53 $$\begin{aligned}&\left( \sum _{\ell ,k}X^{(i)}_{\ell ,k}{\widetilde{O}}_i b_k^\dagger a_\ell ^\dagger b_{k+\ell }+\mathrm {H.c.}\right) \le \epsilon \sum _{k,\ell }\frac{b_{k+\ell }^\dagger b_{k+\ell }}{(|\ell |^2+|k|^2)^2}\nonumber \\&\quad +\epsilon ^{-1}N^{5\kappa -1}\sum _{k} {\widetilde{O}}_i b_k^\dagger \left( \sum _{0<\ell <K} a_\ell ^\dagger a_\ell \right) b_k{\widetilde{O}}_i^\dagger , \end{aligned}$$

where we have used that $X_{\ell ,k}=0$ in case $|\ell |\ge K$. Note at this point that

$$\begin{aligned} {[}a_\ell b_k,{\widetilde{O}}_i^\dagger ]=\gamma _k a_\ell a_k\big [a_0^\dagger \frac{1}{\sqrt{a_0 a_0^\dagger }},{\widetilde{O}}_i^\dagger \big ]+\nu _k a_\ell a_{-k}^\dagger \big [\frac{1}{\sqrt{a_0 a_0^\dagger }}a_0,{\widetilde{O}}_i^\dagger \big ] , \end{aligned}$$

and moreover

$$\begin{aligned}&\big [a_0^\dagger \frac{1}{\sqrt{a_0 a_0^\dagger }},{\widetilde{O}}_i^\dagger \big ]^\dagger \big [a_0^\dagger \frac{1}{\sqrt{a_0 a_0^\dagger }},{\widetilde{O}}_i^\dagger \big ] \Big |_{{\mathcal {F}}^+_{rN}} \lesssim \frac{1}{N^2},\\&\quad \big [\frac{1}{\sqrt{a_0 a_0^\dagger }}a_0,{\widetilde{O}}_i^\dagger \big ]^\dagger \big [\frac{1}{\sqrt{a_0 a_0^\dagger }}a_0,{\widetilde{O}}_i^\dagger \big ]\Big |_{{\mathcal {F}}^+_{rN}} \lesssim \frac{1}{N^2}, \end{aligned}$$

as well as ${\widetilde{O}}_i{\widetilde{O}}_i^\dagger \Big |_{{\mathcal {F}}^+_{rN}}\lesssim 1$. Consequently,

54 $$\begin{aligned} \sum _{k}\! \! {\widetilde{O}}_i b_k^\dagger \! \left( \! \sum _{0<\ell<K} a_\ell ^\dagger a_\ell \! \right) \! b_k{\widetilde{O}}_i^\dagger \Big |_{{\mathcal {F}}^+_{rN}}\! \! \!&\lesssim \! \sum _{k} \! b_k^\dagger \! \left( \! \sum _{0<\ell<K} a_\ell ^\dagger a_\ell \! \right) \! b_k\Big |_{{\mathcal {F}}^+_{rN}}\!\nonumber \\  &\quad \! +\! \frac{1}{N^2}\! \sum _{k\ne 0}\! \gamma _k^2 a_k^\dagger \! \left( \! \sum _{0<\ell<K} a_\ell ^\dagger a_\ell \! \right) \! a_k\Big |_{{\mathcal {F}}^+_{rN}}\nonumber \\&\quad + \! \frac{1}{N^2} \! \sum _{k\ne 0}|\nu _k|^2 a_k\! \left( \sum _{0<\ell<K} a_\ell ^\dagger a_\ell \right) \! a_k^\dagger \Big |_{{\mathcal {F}}^+_{rN}} \nonumber \\&\lesssim \! \sum _{k} b_k^\dagger \! \left( \sum _{0<\ell <K} a_\ell ^\dagger a_\ell \right) \! b_k\Big |_{{\mathcal {F}}^+_{rN}}\! \! +\! N^{2\kappa -1}\! \left( \widetilde{{\mathcal {N}}}+1\right) \! \Big |_{{\mathcal {F}}^+_{rN}} , \end{aligned}$$

where we have used $\sum _{0<\ell <K} a_\ell ^\dagger a_\ell \le N$ and applied Lemma 16 in the last estimate. Using

$$\begin{aligned} \sum _{k,\ell }\frac{1}{(|\ell |^2+|k|^2)^2}b_{k+\ell }^\dagger b_{k+\ell }\lesssim \widetilde{{\mathcal {N}}}, \end{aligned}$$

and that $b_k$ and $b_k^\dagger$ map ${\mathcal {F}}^{\le }_{M_0}$ into ${\mathcal {F}}^{\le }_{M_0+1}$, we have by Eq. (53)

$$\begin{aligned} \left( \sum _{\ell ,k}X^{(i)}_{\ell ,k} {\widetilde{O}}_i b_k^\dagger a_\ell ^\dagger b_{k+\ell } \! + \! \mathrm {H.c.}\right) \! \! \bigg |_{{\mathcal {F}}^{\le }_{M_0}\cap {\mathcal {F}}^+_{rN}} \! \! \! \!&\lesssim \! \! \left( \epsilon \! + \! \epsilon ^{-1}N^{5\kappa }\frac{M_0+1}{N} \! + \! \epsilon ^{-1}N^{5\kappa }N^{2\kappa -2}\right) \! \! \left( \widetilde{{\mathcal {N}}} \! + \! 1\right) \bigg |_{{\mathcal {F}}^{\le }_{M_0}\cap {\mathcal {F}}^+_{rN}}\\&\lesssim N^{\frac{5\kappa }{2}}\sqrt{\frac{M_0+1}{N}+N^{2\kappa -2}}\left( \widetilde{{\mathcal {N}}}\bigg |_{{\mathcal {F}}^{\le }_{M_0}\cap {\mathcal {F}}^+_{rN}}+1\right) \end{aligned}$$

for an optimal choice of $\epsilon$. Regarding the *Y*-contributions, we are going to estimate

55 $$\begin{aligned} \left( \sum _{\ell ,k}Y^{(i)}_{\ell ,k} {\widetilde{O}}_i a_\ell ^\dagger b_{k+\ell }b_{-k} +\mathrm {H.c.}\right) \lesssim \epsilon \, \widetilde{{\mathcal {N}}}+\epsilon ^{-1}\sum _{k,\ell ,\ell '}\overline{Y_{\ell ',k}} Y_{\ell ,k}\, {\widetilde{O}}_i a^\dagger _\ell b_{\ell +k}b_{\ell '+k}^\dagger a_{\ell '}{\widetilde{O}}_i^\dagger . \end{aligned}$$

Defining the operator *G* via its coefficients

$$\begin{aligned} G_{\ell k, \ell ' k'}:=\delta _{\ell +k=\ell '+k'}\overline{Y_{\ell ',k-\ell '}} Y_{\ell ,k-\ell }, \end{aligned}$$

and using the fact that $b_k$ satisfies the CCR on ${\mathcal {F}}^+_{N-1}$ as well as $a_\ell {\mathcal {F}}^+_{N-1}\subset {\mathcal {F}}^+_{N-2}$, we furthermore obtain

$$\begin{aligned} \sum _{k,\ell ,\ell '} \! \overline{Y_{\ell ',k}} Y_{\ell ,k}\,{\widetilde{O}}_i a^\dagger _\ell b_{\ell +k}b_{\ell '+k}^\dagger a_{\ell '}{\widetilde{O}}_i^\dagger \Big |_{{\mathcal {F}}^+_{N-2}} \! \! \! \! \! \! \!&= \! \sum _{k,\ell } \! \overline{Y_{\ell ,k}} Y_{\ell ,k}\,a^\dagger _\ell {\widetilde{O}}_i {\widetilde{O}}_i^\dagger a_{\ell }\Big |_{{\mathcal {F}}^+_{N-2}} \! \! \! \! \! \! \\  &\quad + \! \sum _{k\ell ,k\ell '} \! G_{\ell k, \ell ' k'}{\widetilde{O}}_i a_\ell ^\dagger b_k^\dagger b_{k'}a_{\ell '}{\widetilde{O}}_i^\dagger \Big |_{{\mathcal {F}}^+_{N-2}}. \end{aligned}$$

Note that we have by Lemma 16 the operator inequalities

$$\begin{aligned} \sum _{k,\ell }\overline{Y_{\ell ,k}} Y_{\ell ,k}\,a^\dagger _\ell {\widetilde{O}}_i {\widetilde{O}}_i^\dagger a_{\ell }\lesssim \sum _{k,\ell }\overline{Y_{\ell ,k}} Y_{\ell ,k}\,a^\dagger _\ell a_{\ell }\lesssim N^{5\kappa -1}{\mathcal {N}}\lesssim N^{5\kappa }N^{\frac{3\kappa }{2}-1}\left( \widetilde{{\mathcal {N}}}+1\right) . \end{aligned}$$

Using furthermore the bound on the operator norm $\Vert G\Vert \lesssim N^{5\kappa -1}$ yields

$$\begin{aligned} \pm \! \! \sum _{k\ell ,k\ell '} \! G_{\ell k, \ell ' k'} {\widetilde{O}}_i a_\ell ^\dagger b_k^\dagger b_{k'}a_{\ell '}{\widetilde{O}}_i^\dagger \!&\lesssim N^{5\kappa -1}\sum _{0<|\ell |<K,k}{\widetilde{O}}_i a_\ell ^\dagger b_k^\dagger b_k a_\ell {\widetilde{O}}_i^\dagger \\&\lesssim \! N^{5\kappa -1} \! \sum _k \! {\widetilde{O}}_i b_k^\dagger \! \! \left( \! \sum _{0<|\ell |<K} \! \! a_\ell ^\dagger a_\ell \! \! \right) \! b_k{\widetilde{O}}_i^\dagger \! \\&\quad + \! N^{5\kappa -1} \! \sum _k \! {\widetilde{O}}_i [b_k, \! a_{-k}]^\dagger [b_k, \! a_{-k}]{\widetilde{O}}_i^\dagger \! . \end{aligned}$$

Furthermore, $$\sum _k {\widetilde{O}}_i [b_k,a_{-k}]^\dagger [b_k,a_{-k}]{\widetilde{O}}_i^\dagger \Big |_{{\mathcal {F}}^{+}_{rN}} \lesssim \sum _k |\nu _k|^2{\widetilde{O}}_i {\widetilde{O}}_i^\dagger \Big |_{{\mathcal {F}}^{+}_{rN}}\lesssim \sum _k |\nu _k|^2\lesssim N^{\frac{3\kappa }{2}}$$, hence

56 $$\begin{aligned} \sum _{k,\ell ,\ell '}\overline{Y_{\ell ',k}} Y_{\ell ,k}\, {\widetilde{O}}_i a^\dagger _\ell b_{\ell +k}b_{\ell '+k}^\dagger a_{\ell '}{\widetilde{O}}_i^\dagger \!&\lesssim \! N^{5\kappa -1}\sum _k {\widetilde{O}}_i b_k^\dagger \! \left( \sum _{0<|\ell |<K}a_\ell ^\dagger a_\ell \right) \! b_k{\widetilde{O}}_i^\dagger \! \nonumber \\  &\quad + \! N^{5\kappa -1}N^{\frac{3\kappa }{2}} \! \left( \widetilde{{\mathcal {N}}} \! + \! 1 \! \right) \! . \end{aligned}$$

Therefore, we obtain in combination with Eq. (54)

$$\begin{aligned} \pm \! \! \sum _{k,\ell ,\ell '}\overline{Y_{\ell ',k}} Y_{\ell ,k}\, {\widetilde{O}}_i a^\dagger _\ell b_{\ell +k}b_{\ell '+k}^\dagger a_{\ell '}{\widetilde{O}}_i^\dagger \Big |_{{\mathcal {F}}^{\le }_{M_0}\cap {\mathcal {F}}^{+}_{rN}} \! \! \!&\lesssim \! N^{5\kappa } \! \left( \! \frac{M_0 \! + \! 1}{N} \! + \! N^{2\kappa -2} \! + \! N^{\frac{3\kappa }{2}-1}\right) \!\\  &\quad \times \left( \widetilde{{\mathcal {N}}}\Big |_{{\mathcal {F}}^{\le }_{M_0}\cap {\mathcal {F}}^{+}_{rN}} \! \! + \! 1 \! \right) \! . \end{aligned}$$

Using the optimal choice $\epsilon := N^{\frac{5\kappa }{2}}\sqrt{\frac{M_0+1}{N}+N^{\frac{3\kappa }{2}-1}}$ concludes the proof of Eq. (52). Regarding the second statement of the Lemma, note that

$$\begin{aligned} \sum _{0<\ell <K} a_\ell ^\dagger a_\ell \lesssim N^{\frac{3\kappa }{2}-1}\! \left( \widetilde{{\mathcal {N}}}+1\right) \end{aligned}$$

by Lemma 16, and therefore we obtain by Eq. (54)

$$\begin{aligned} \sum _{k}\! \! {\widetilde{O}}_i b_k^\dagger \! \left( \! \sum _{0<\ell <K} a_\ell ^\dagger a_\ell \! \right) \! b_k{\widetilde{O}}_i^\dagger \Big |_{{\mathcal {F}}^+_{rN}} \! \! \lesssim N^{\frac{3\kappa }{2}}\! \left( \widetilde{{\mathcal {N}}}+1\right) ^2\Big |_{{\mathcal {F}}^+_{rN}} \! . \end{aligned}$$

In combination with Eq. (53), respectively Eq. (55) and Eq. (56), this concludes the proof with the concrete choice $\epsilon :=N^{\frac{13\kappa }{4}-\frac{1}{2}}$. $\square$

At this point we want to emphasise that our error estimates in Lemma 6 and Lemma 7 can only produce good bounds in case we apply them for states in $\Psi \in {\mathcal {F}}^\le _M$ with suitably small number of particles *M*. As we will demonstrate in Appendix C, we can always restrict our attention to such states.

### Proof of Theorem 2

In the following let $0<\kappa <\frac{1}{8}$ and $d\in {\mathbb {N}}$, and let us introduce the concrete choices $\lambda _0:=\frac{3}{8}$, $\lambda :=\frac{21}{32}$ and $K:=N^{\frac{5}{16}+\frac{\kappa }{2}}$. As we explain later in this subsection, we can assume

$$\begin{aligned} E^{(d)}_{N,\kappa }\le E_{N,\kappa }+CN^{\frac{\kappa }{2}} \end{aligned}$$

without loss of generality, and therefore there exists a *d* dimensional subspace ${\mathcal {V}}_d \subseteq {\mathcal {F}}^{\le }_{N^{\lambda _0}}\cap {\mathcal {F}}^+_{N^\lambda }$, such that according to Lemma 18

57 $$\begin{aligned} E^{(d)}_{N,\kappa }\ge \sup _{\Psi \in {\mathcal {V}}_d:\Vert \Psi \Vert =1} {\langle {\Psi , H_{N,\kappa }\Psi }\rangle }-C N^{-\tau }, \end{aligned}$$

with $\tau :=\frac{5}{2} \! \left( \frac{1}{8}-\kappa \right)$. We want to emphasise at this point that Lemma 18 depends crucially on the a priori estimates derived in [6] as we explain in Appendix C. In order to control the error terms ${\mathcal {E}}_i$, we first observe that for any $\Psi$ in ${\mathcal {V}}_d$ with $\Vert \Psi \Vert =1$ we obtain

$$\begin{aligned} {\langle {\Psi ,{\mathcal {E}}_2 \Psi }\rangle }\le \frac{1}{N}{\langle {\Psi ,\sum _{0<|k|<K} \sigma _k {\mathcal {N}}a_k^\dagger a_k \Psi }\rangle }&\lesssim N^{\kappa +\lambda _0-1}{\langle {\Psi ,{\mathcal {N}}\Psi }\rangle }\\  &\lesssim N^{\frac{5}{2}\kappa +\lambda _0-1}{\langle {\Psi ,\big (\widetilde{{\mathcal {N}}}+1\big )\Psi }\rangle }, \end{aligned}$$

where we used Lemma 16 and $\widetilde{{\mathcal {N}}}$ is defined above Lemma 6. Again by Lemma 16, we have

$$\begin{aligned} | {\langle {\Psi , \! \! \sum _{k\ne 0}|k|^2 |w_k|^2 N^{-2}\left( a_0^{2\dagger }a_0^2 \! - \! N^2 \right) a_k^\dagger a_k\Psi }\rangle } | \!&\lesssim \! N^{2\kappa -1} \! \! {\langle {\Psi ,{\mathcal {N}} \! \left( {\mathcal {N}} \! + \! 1\right) \Psi }\rangle } \! \\  &\lesssim \! N^{\frac{5\kappa }{2}+\lambda -1}\! {\langle {\Psi ,\left( \widetilde{{\mathcal {N}}} \! + \! 1\right) \Psi }\rangle } \! , \end{aligned}$$

which is a term appearing in the definition of ${\mathcal {E}}_1$ in Eq. (46). Using the representation of ${\mathcal {E}}_1$ and ${\mathcal {E}}_3$ from Eq. (46) and Eq. (47), the estimates from Lemmata 6, 7 and 15, as well as Eq. (95) and Eq. (94), we therefore obtain

$$\begin{aligned} \left\langle \Psi ,\sum _{i=1}^4 {\mathcal {E}}_i \Psi \right\rangle \!&\lesssim \! N^{-\tau } \! \left\langle \! \Psi ,\left( \! \widetilde{{\mathcal {N}}} \! + \! \sum _{k}|k|^{1-2\delta }b_k^\dagger b_k \! + \! 1 \! \right) \Psi \! \right\rangle \! \\  &\le \! N^{-\tau } \! \left\langle \! \Psi ,\left( \! \sum _{k}|k|^{1-2\delta }b_k^\dagger b_k \! + \! 1\right) \Psi \! \right\rangle . \end{aligned}$$

Furthermore, we have by Eq. (93)

$$\begin{aligned} \left\langle \! \Psi ,\left( \! \sum _{k}|k|^{1-2\delta }b_k^\dagger b_k \! + \! 1\right) \Psi \! \right\rangle {\lesssim } N^{-\frac{\kappa }{2}}\left\langle \! \Psi ,\sum _{k\ne 0}e_k\left( \gamma _k d_k \! + \! \nu _k d_{-k}^\dagger \right) ^\dagger \left( \gamma _k d_k \! + \! \nu _k d_{-k}^\dagger \right) \Psi \! \right\rangle {+} 1. \end{aligned}$$

Combining what we have so far with the lower bound in Eq. (45) yields for a suitable $C>0$

58 $$\begin{aligned}&{\langle {\Psi , H_{N,\kappa }\Psi }\rangle }\ge 4\pi {\mathfrak {a}}_{N^{1-\kappa }}N^\kappa (N-1) + \frac{1}{2}\sum _{k\ne 0}\left\{ \sqrt{A_k^2-B_k^2}-A_k+C_k\right\} -C N^{-\tau }\nonumber \\&\quad +\left( 1-CN^{-\tau }N^{-\frac{\kappa }{2}}\right) \left\langle \! \Psi ,\sum _{k\ne 0}e_k\left( \gamma _k d_k \! + \! \nu _k d_{-k}^\dagger \right) ^\dagger \left( \gamma _k d_k \! + \! \nu _k d_{-k}^\dagger \right) \Psi \! \right\rangle . \end{aligned}$$

We furthermore obtain by Lemma 13 the following estimate on $\sum _{k\ne 0}\left\{ \sqrt{A_k^2-B_k^2}-A_k+C_k\right\}$

59 $$\begin{aligned}&\left| \sum _{k\ne 0}\left\{ \sqrt{A_k^2-B_k^2}-A_k+C_k\right\} -\sum _{k\ne 0}\left\{ \sqrt{|k|^4+16\pi {\mathfrak {a}} N^\kappa |k|^2}-|k|^2-8\pi {\mathfrak {a}} N^\kappa \right. \right. \nonumber \\  &\left. \left. +\frac{(8\pi {\mathfrak {a}} N^\kappa )^2}{2|k|^2}\right\} \right| \nonumber \quad \lesssim N^{4\kappa -1}\log N+\frac{N^{3\kappa }}{K}\lesssim N^{-\tau }. \end{aligned}$$

Using $e_k\ge 0$ in Eq. (58) and the state $\Psi _0$ which spans the space ${\mathcal {V}}_1$, we can consequently verify the first statement Eq. (4)

$$\begin{aligned} E^{(1)}_{N,\kappa } \!&\ge \! \! {\langle {\Psi _0, H_{N,\kappa }\Psi _0}\rangle } \! - \! C_1 N^{-\tau } \\&\ge \! 4\pi {\mathfrak {a}}_{N^{1-\kappa }}N^\kappa (N \! - \! 1) \! + \! \frac{1}{2} \sum _{k\ne 0} \! \left\{ \! \sqrt{A_k^2 \! - \! B_k^2} \! - \! A_k \! + \! C_k \! \right\} \! - \! C_2 N^{-\tau }\\&\ge \! 4\pi {\mathfrak {a}}_{N^{1-\kappa }}N^\kappa (N \! - \! 1) \! + \! \frac{1}{2}\sum _{k\ne 0} \! \left\{ \! \sqrt{|k|^4 \! + \! 16\pi {\mathfrak {a}} N^\kappa |k|^2} \! - \! |k|^2 \! - \! 8\pi {\mathfrak {a}} N^\kappa \! + \! \frac{(8\pi {\mathfrak {a}} N^\kappa )^2}{2|k|^2} \! \right\} \! \\  &\quad - \! C_3 N^{-\tau } \!, \end{aligned}$$

where $C_1,C_2,C_3>0$ are suitable constants.

In order to verify the second statement of Theorem 2, recall the definition of $\lambda ^{(d)}_{N,\kappa }$ in Theorem 2 and let us define ${\widetilde{e}}_k:=\min \{e_k,\lambda ^{(d)}_{N,\kappa }+1\}$. By Eq. (58) we obtain for a suitable constant $C>0$ the lower bound

60 $$\begin{aligned}&{\langle {\Psi , H_{N,\kappa }\Psi }\rangle }\ge 4\pi {\mathfrak {a}}_{N^{1-\kappa }}N^\kappa (N-1) + \frac{1}{2}\sum _{k\ne 0}\left\{ \sqrt{A_k^2-B_k^2}-A_k+C_k\right\} -CN^{-\tau }\nonumber \\&\quad +\left( 1-CN^{-\tau }N^{-\frac{\kappa }{2}}\right) \left\langle \! \Psi ,\sum _{k\ne 0}{\widetilde{e}}_k\left( \gamma _k d_k \! + \! \nu _k d_{-k}^\dagger \right) ^\dagger \left( \gamma _k d_k \! + \! \nu _k d_{-k}^\dagger \right) \Psi \! \right\rangle . \end{aligned}$$

Since $|{\widetilde{e}}_k|\lesssim N^{\frac{\kappa }{2}}$ uniformly in *k*, we furthermore have by Eq. (92) for $\Psi \in {\mathcal {V}}_d$

$$\begin{aligned}&\left\langle \Psi ,\left\{ \sum _{k\ne 0}{\widetilde{e}}_k b_k^\dagger b_k -\sum _{k\ne 0} {\widetilde{e}}_k\left( \gamma _k d_k \! + \! \nu _k d_{-k}^\dagger \right) ^\dagger \left( \gamma _k d_k \! + \! \nu _k d_{-k}^\dagger \right) \right\} \Psi \right\rangle \\&\quad \lesssim \left( N^{\frac{5\kappa +\lambda _0-1}{2}}+N^{2\kappa +\lambda -1}\right) \left( {\langle {\Psi ,\widetilde{{\mathcal {N}}}\Psi }\rangle }+1\right) \\&\quad \lesssim N^{-\tau }\left( {\langle {\Psi ,\widetilde{{\mathcal {N}}}\Psi }\rangle }+1\right) , \end{aligned}$$

where the operators $b_k$ are introduced above Lemma 6. In combination with Eq. (59), Eq. (57) and Eq. (60), we therefore obtain for a suitable constant $C>0$

$$\begin{aligned} E_{N,\kappa }^{(d)}&\ge 4\pi {\mathfrak {a}}_{N^{1-\kappa }}N^\kappa (N-1) + \frac{1}{2}\sum _{k\ne 0} \left\{ \sqrt{|k|^4+16\pi {\mathfrak {a}} N^\kappa |k|^2}-|k|^2-8\pi {\mathfrak {a}} N^\kappa \right. \\  &\quad \left. +\frac{(8\pi {\mathfrak {a}} N^\kappa )^2}{2|k|^2}\right\} +\left( 1-C N^{-\tau }N^{-\frac{\kappa }{2}}\right) \sup _{\Psi \in {\mathcal {V}}_d:\Vert \Psi \Vert =1}\left\langle \! \Psi ,\sum _{k\ne 0}{\widetilde{e}}_k b_k^\dagger b_k\Psi \! \right\rangle \\  &\quad -CN^{-\tau }. \end{aligned}$$

Following the work [5], respectively by making use of the excitation map $U_N$ introduced in [14], we note that the operators $b_k$ can be extended from ${\mathcal {F}}^+_{N-1}$ to operators satisfying the CCR, to be precise there exists a Hilbert space extension $L^2_{\textrm{sym}} \! \left( \Lambda ^N\right) \subseteq {\mathfrak {h}}$ and operators ${\mathfrak {b}}_k$ defined on ${\mathfrak {h}}$, such that the family $\{{\mathfrak {b}}_k:k\in 2\pi {\mathbb {Z}}^3\setminus \{0\}\}$ is unitarily equivalent to the standard annihilation operators and ${\mathfrak {b}}_k \Psi =b_k \Psi$ for $\Psi \in {\mathcal {F}}^+_{N-1}$. Denoting the *d*-th eigenvalue of $\sum _{k\ne 0}{\widetilde{e}}_k {\mathfrak {b}}_k^\dagger {\mathfrak {b}}_k$ as ${\widetilde{\lambda }}^{(d)}_{N,\kappa }$ and making use of the fact that ${\mathcal {V}}_d\subseteq {\mathcal {F}}^+_{N-1}$ for *N* large enough, we obtain by the min-max principle

61 $$\begin{aligned} \sup _{\Psi \in {\mathcal {V}}_d:\Vert \Psi \Vert =1}\left\langle \! \Psi ,\sum _{k\ne 0}{\widetilde{e}}_k b_k^\dagger b_k\Psi \! \right\rangle = \sup _{\Psi \in {\mathcal {V}}_d:\Vert \Psi \Vert =1}\left\langle \! \Psi ,\sum _{k\ne 0}{\widetilde{e}}_k {\mathfrak {b}}_k^\dagger {\mathfrak {b}}_k\Psi \! \right\rangle \ge {\widetilde{\lambda }}^{(d)}_{N,\kappa }. \end{aligned}$$

Since the operators ${\mathfrak {b}}_k$ are unitarily equivalent to standard annihilation operators, we know that the eigenvalues ${\widetilde{\lambda }}^{(d)}_{N,\kappa }$ are an enumeration of $\left\{ \sum _{k\ne 0} n_k {\widetilde{e}}_k: n_k\in {\mathbb {N}}_0\right\}$ in increasing order. Using the fact that

$$\begin{aligned} \left| |k|^2+8\pi {\mathfrak {a}} N^{\kappa }-2A_k\right| \lesssim N^{2\kappa -1}(1+|k|) \end{aligned}$$

for $|k|<K$ and $\left| 8\pi {\mathfrak {a}} N^{\kappa }-2B_k\right| \lesssim N^{2\kappa -1}(1+|k|)$, we obtain

$$\begin{aligned} |e_k-\sqrt{|k|^4+16\pi {\mathfrak {a}} N^\kappa |k|^2}|\lesssim N^{2\kappa -1} \end{aligned}$$

for fixed *k*, and consequently $|{\widetilde{\lambda }}^{(d)}_{N,\kappa }-\lambda ^{(d)}_{N,\kappa }|\lesssim N^{2\kappa -1}\le N^{-\tau }$. Using that $|\lambda ^{(d)}_{N,\kappa }|\lesssim N^{\frac{\kappa }{2}}$, yields for suitable constants $C,C'>0$

62 $$\begin{aligned}&E_{N,\kappa }^{(d)} - 4\pi {\mathfrak {a}}_{N^{1-\kappa }}N^\kappa (N-1) \\  &- \frac{1}{2}\sum _{k\ne 0}\left\{ \sqrt{|k|^4+16\pi {\mathfrak {a}} N^\kappa |k|^2}-|k|^2-8\pi {\mathfrak {a}} N^\kappa \right. \nonumber \left. +\frac{(8\pi {\mathfrak {a}} N^\kappa )^2}{2|k|^2}\right\} \nonumber \\  &\quad \ge \left( 1-C' N^{-\tau }N^{-\frac{\kappa }{2}}\right) \lambda ^{(d)}_{N,\kappa }- C' N^{-\tau }\ge \lambda ^{(d)}_{N,\kappa }-CN^{-\tau }. \end{aligned}$$

We also want to emphasize that the constant $C>0$ in the lower bound in Eq. (61) depends uniformly on *V* in the sense of Eq. (12). Together with Theorem 3, this concludes the proof of Theorem 2.

## Trial States and Their Energy

In this Section we want to verify the upper bound on $E_{N,\kappa }$ in Theorem 3. For this purpose let us use the concrete choice $K:=\infty$ for the cut-off parameter and let us keep the term

$$\begin{aligned} \frac{1}{2}\sum _{j k,mn} \left( \pi _{\mathcal {H}} V_{N^{1-\kappa }}\pi _{\mathcal {H}}\right) _{j k,mn}\psi _{j k}^\dagger \psi _{m n}=\frac{1}{2}\sum _{j k,mn\ne 0} \left( V_{N^{1-\kappa }}\right) _{j k,mn}\psi _{j k}^\dagger \psi _{m n}, \end{aligned}$$

which we have bounded from below by 0 in Eq. (20), yielding the identity

63 $$\begin{aligned} H'_{N,\kappa } \! = \! \sum _{k\ne 0}e_k\left( \gamma _k d_k \! + \! \nu _k d_{-k}^\dagger \right) ^\dagger \! \left( \gamma _k d_k \! + \! \nu _k d_{-k}^\dagger \right) \! + \! \frac{1}{2}\sum _{j k,mn\ne 0} \left( V_{N^{1-\kappa }}\right) _{j k,mn}\psi _{j k}^\dagger \psi _{m n} \! - \! \sum _{i=1}^4 {\mathcal {E}}_i, \end{aligned}$$

with the excitation Hamiltonian $H'_{N,\kappa }$ defined as

$$\begin{aligned} H'_{N,\kappa }:=H_{N,\kappa }-4\pi {\mathfrak {a}}_{N^{1-\kappa }}N^\kappa (N-1)- \frac{1}{2}\sum _{k\ne 0}\left\{ \sqrt{A_k^2-B_k^2}-A_k+C_k\right\} . \end{aligned}$$

In order to obtain from this representation of $H_{N,\kappa }$ an upper bound on its eigenvalues $E_{N,\kappa }^{(d)}$, we need to find trial states $\Psi _d$ which simultaneously annihilate the variables $\gamma _k d_k+\nu _k d_{-k}^\dagger$ for $k\ne 0$ and $\psi _{j k}$ for $j,k\ne 0$, at least in an approximate sense. This will be carried out in the following two Sects. 5.1 and 5.2, using the operators ${\mathfrak {b}}_k$ introduced above Eq. (60) as well as ${\mathfrak {a}}_k:=\gamma _k {\mathfrak {b}}_k-\nu _k {\mathfrak {b}}_{-k}^\dagger$, which is an extension of $a_0^\dagger \frac{1}{\sqrt{a_0 a_0^\dagger }}a_k$ from ${\mathcal {F}}^+_{N-1}$ to ${\mathfrak {h}}$.

### Annihilation of $\gamma _k d_k+\nu _k d_{-k}^\dagger$

We start by expressing $\gamma _k d_k+\nu _k d_{-k}^\dagger$ in terms of the extensions ${\mathfrak {b}}_k$ of the operators $b_k$. In order to do this, note that

$$\begin{aligned} \gamma _k d_k+\nu _k d_{-k}^\dagger =b_k-\gamma _k \delta _k-\nu _k \delta _{-k}^\dagger , \end{aligned}$$

where $\delta _k$ is as in Lemma 4, and $b_k \Psi ={\mathfrak {b}}_k \Psi$, as well as $a_0^\dagger \frac{1}{\sqrt{a_0 a_0^\dagger }}a_k \Psi ={\mathfrak {a}}_k \Psi$, for states $\Psi \in {\mathcal {F}}^+_{N-1}$. Therefore, we obtain

64 $$\begin{aligned} \left( \gamma _k d_k+\nu _k d_{-k}^\dagger \right) \Psi =\left( {\mathfrak {b}}_k+\sum _p \sqrt{N}f_{p,k}{\mathfrak {b}}^\dagger _{p-k}{\mathfrak {a}}_p+\epsilon _k\right) \Psi , \end{aligned}$$

for $\Psi \in {\mathcal {F}}^+_{N-1}$, with $\delta _k''$ as in Lemma 4 and

$$\begin{aligned} \epsilon _k&=-\nu _k \delta _{-k}^\dagger -\gamma _k \delta _k''+\sum _p\left( \gamma _k\gamma _{p-k}\sqrt{a_0^\dagger a_0}-\sqrt{N}\right) f_{p,k}{\mathfrak {b}}^\dagger _{p-k}{\mathfrak {a}}_p\\&\quad +\sum _{p}\gamma _k \nu _{p-k}\sqrt{a_0^\dagger a_0}f_{p,k} {\mathfrak {b}}_{k-p}{\mathfrak {a}}_p. \end{aligned}$$

In the following Lemma 8 we show that the contribution coming from the $\epsilon _k$ can be regarded as a small error.

#### Lemma 8

There exists a constant $C>0$, such that we have for $\kappa <1$ the estimate

$$\begin{aligned} \sum _k e_k\, \epsilon _k^\dagger \epsilon _k\le C N^{6\kappa -1}\! \left( \widetilde{{\mathcal {N}}}+1\right) ^3. \end{aligned}$$

#### Proof

Due to the positivity of $e_k$ we can estimate the square separately for each term in the definition of $\epsilon _k$. Starting with $-\nu _k \delta _{-k}^\dagger$ we obtain by a slight adaptation of Eq. (96)

$$\begin{aligned} \sum _{k\ne 0}e_k \left( \nu _k \delta _{-k}^\dagger \right) ^\dagger \nu _k \delta _{-k}^\dagger \lesssim \left( \sum _{k\ne 0}e_k \nu _k^2 |k|^{-4}\right) N^{5\kappa -1}\! \left( \widetilde{{\mathcal {N}}}+1\right) ^3\lesssim N^{6\kappa -1}\! \left( \widetilde{{\mathcal {N}}}+1\right) ^3. \end{aligned}$$

By a slight modification of the upper bound on $\left( \delta _k''\right) ^\dagger \delta _k''$ obtained in Lemma 17, we have

$$\begin{aligned} \sum _{k\ne 0}e_k \left( \delta _k''\right) ^\dagger \delta _k''\lesssim \frac{\sum _{k\ne 0}e_k \gamma _k^2 |w_k|^2}{N}N^{\frac{11\kappa }{2}-1} \! \left( \widetilde{{\mathcal {N}}}+1\right) \lesssim N^{\frac{11\kappa }{2}-1} \! \left( \widetilde{{\mathcal {N}}}+1\right) . \end{aligned}$$

Regarding the term $\sum _p\left( \gamma _k\gamma _{p-k}\sqrt{\frac{a_0^\dagger a_0}{N}}-1\right) \sqrt{N}f_{p,k}{\mathfrak {b}}^\dagger _{p-k}{\mathfrak {a}}_p=X_k+{\widetilde{X}}_k$ with

$$\begin{aligned} X_k&:= \sum _p\left( \sqrt{\frac{a_0^\dagger a_0}{N}}-1\right) \sqrt{N}f_{p,k}{\mathfrak {b}}^\dagger _{p-k}{\mathfrak {a}}_p, \\ {\widetilde{X}}_k&:= \sum _p\left( \gamma _k\gamma _{p-k}-1\right) \sqrt{\frac{a_0^\dagger a_0}{N}} \sqrt{N}f_{p,k}{\mathfrak {b}}^\dagger _{p-k}{\mathfrak {a}}_p, \end{aligned}$$

note that we have the estimates

$$\begin{aligned} \left| \sqrt{\frac{a_0^\dagger a_0}{N}}-1\right| ^2&\lesssim \frac{{\mathcal {N}}^2}{N^2},\\ \left| \left( \gamma _k\gamma _{p-k}-1\right) \sqrt{\frac{a_0^\dagger a_0}{N}}\right| ^2&\lesssim \frac{N^{\kappa }}{|k|^{2}}+\frac{N^{\kappa }}{|p-k|^{2}}. \end{aligned}$$

Since $e_k\lesssim N^{\frac{\kappa }{2}}|k|^2$, we therefore have

$$\begin{aligned} \sum _{k\ne 0}e_k X_k^\dagger X_k&\lesssim N^{\frac{\kappa }{2}}\sum _{k\ne 0}|k|^2 X_k^\dagger X_k\lesssim N^{\frac{3\kappa }{2}}\frac{{\mathcal {N}}^3}{N^2}\lesssim N^{6\kappa -2}(\widetilde{{\mathcal {N}}}+1)^3,\\ \sum _{k\ne 0} \! e_k {\widetilde{X}}_k^\dagger {\widetilde{X}}_k \!&\lesssim \! N^{\frac{3\kappa }{2}} \! \sup _{p} \! \left\{ \! \! \sum _k \! |k|^2 \big (\sqrt{N}f_{p,k}\big )^2 \! \! \left( \frac{1}{|k|^2} \! + \! \frac{1}{|p-k|^2}\right) \! \! \right\} \! \sum _p \! {\mathfrak {a}}_p^\dagger \! \left( \! \widetilde{{\mathcal {N}}} \! + \! 1 \! \right) \! {\mathfrak {a}}_p \! \\  &\lesssim \! \frac{N^{5\kappa }}{N}\! \left( \! \widetilde{{\mathcal {N}}} \! + \! 1 \! \right) ^2, \end{aligned}$$

where we have used ${\mathcal {N}}^3\lesssim N^{\frac{9\kappa }{2}}\left( \widetilde{{\mathcal {N}}}+1\right) ^m$, $\sum _k |k|^2 \big (\sqrt{N}f_{p,k}\big )^2 \! \left( \frac{1}{|k|^2} \! + \! \frac{1}{|p-k|^2}\right) \lesssim N^{2\kappa -1}$ and $\sum _p a_p^\dagger \left( \widetilde{{\mathcal {N}}} \! + \! 1\right) a_p\lesssim N^{\frac{3\kappa }{2}}\! \left( \widetilde{{\mathcal {N}}}+1\right) ^2$. The final term in $\epsilon _k$ can be estimated similarly.

$$\square $$

In order to cancel the term $\sum _p \sqrt{N}f_{p,k}{\mathfrak {b}}^\dagger _{p-k}{\mathfrak {a}}_p$ in Eq. (64), let us introduce the operators

$$\begin{aligned} G&:= \frac{1}{2}\sum _{p,k}\sqrt{N}f_{p,k}{\mathfrak {b}}_k^\dagger {\mathfrak {b}}_{p-k}^\dagger {\mathfrak {a}}_{p},\\ \Theta _\ell&:= \frac{1}{2}\sum _{p}\nu _\ell \sqrt{N}f_{-\ell ,p}{\mathfrak {b}}^\dagger _{-(\ell +p)}{\mathfrak {b}}^\dagger _p, \end{aligned}$$

which allows us to write

65 $$\begin{aligned} \left( \gamma _k d_k+\nu _k d_{-k}^\dagger \right) \! \left( 1 \! - \! G \right) \Psi {=}\left( {\mathfrak {b}}_k - \Theta _k \! + \! \epsilon _k \! \left( 1 \! - \! G \right) \! {- }\! G{\mathfrak {b}}_k \! {-} \! \left( \sum _p \sqrt{N}f_{p,k}{\mathfrak {b}}^\dagger _{p-k}{\mathfrak {a}}_p\right) \! G\right) \Psi \end{aligned}$$

for $\Psi \in {\mathcal {F}}^+_{N-2}$, where we have used that $(1-G){\mathcal {F}}^+_{N-2}\subset {\mathcal {F}}^+_{N-1}$.

#### Corollary 2

Let us define the coefficients $\Pi _k$ and operators $\Xi _k$ as

$$\begin{aligned} \Pi _k&:= \frac{1}{4}\sum _{j\ne -k} e_{j+k}\nu _{j+k}^2 N f_{j+k,j}\! \left( f_{j+k,j}+f_{j+k,k}\right) ,\\ \Xi _k&:= \left( \gamma _k d_k+\nu _k d_{-k}^\dagger \right) \! \left( 1 \! - \! G \right) -{\mathfrak {b}}_k+\Theta _k. \end{aligned}$$

Then there exists a $C>0$, such that

66 $$\begin{aligned}&\sum _k e_k\Xi _k^\dagger \, \Xi _k\Big |_{{\mathcal {F}}^+_{N-2}} \le C N^{\frac{11\kappa }{2}-1} \! \left( \widetilde{{\mathcal {N}}}+1\right) ^2 \! \left( \sum _k e_k {\mathfrak {b}}_k^\dagger {\mathfrak {b}}_k+1\right) \Big |_{{\mathcal {F}}^+_{N-2}}. \end{aligned}$$

Furthermore, we have $|\sum _k \Pi _k|\le C N^{\frac{9\kappa }{2}}\frac{\log N}{N}$ as well as

$$\begin{aligned} \pm \left( \sum _\ell e_\ell \Theta _\ell ^\dagger \Theta _\ell -\sum _k \Pi _k\right) \le C N^{\frac{9\kappa }{2}-1} \left( \widetilde{{\mathcal {N}}}+1\right) ^2 \! \left( \sum _k e_k {\mathfrak {b}}_k^\dagger {\mathfrak {b}}_k+1\right) . \end{aligned}$$

#### Proof

Defining $X_k:=\left( \sum _p \sqrt{N}f_{p,k}{\mathfrak {b}}^\dagger _{p-k}{\mathfrak {a}}_p\right) \! G$, we obtain by Eq. (65)

67 $$\begin{aligned} \sum _k e_k\Xi _k^\dagger \, \Xi _k\Big |_{{\mathcal {F}}^+_{N-2}} \!&\lesssim \! \sum _k e_k (1\! - \! G)^\dagger \epsilon _{k}^\dagger \epsilon _{k}(1\! - \! G)\Big |_{{\mathcal {F}}^+_{N-2}} \! \! + \! \sum _{k\ne 0} e_k {\mathfrak {b}}_k^\dagger G^\dagger G {\mathfrak {b}}_k\Big |_{{\mathcal {F}}^+_{N-2}} \! \! \nonumber \\  &\quad + \! \sum _{k\ne 0}e_k X_k^\dagger X_k\Big |_{{\mathcal {F}}^+_{N-2}} \! \! . \end{aligned}$$

First of all note that we have by Lemma 8

$$\begin{aligned} \sum _{ k\ne 0}e_k (1\! - \! G)^\dagger \epsilon _{k}^\dagger \epsilon _{k}(1\! - \! G)&\lesssim N^{6\kappa -1}\! (1\! - \! G)^\dagger \left( \widetilde{{\mathcal {N}}}+1\right) ^3(1\! - \! G)\\&\lesssim N^{\frac{11\kappa }{2}-1}\left( \widetilde{{\mathcal {N}}}+1\right) ^2\left( \sum _k e_k {\mathfrak {b}}_k^\dagger {\mathfrak {b}}_k+1\right) . \end{aligned}$$

Furthermore, we have that $G^\dagger G\lesssim N^{2\kappa -1}\! \left( {\mathcal {N}}+1\right) ^2\lesssim N^{5\kappa -1}\! \left( \widetilde{{\mathcal {N}}}+1\right) ^2$, and consequently

$$\begin{aligned} \sum _{k\ne 0} e_k {\mathfrak {b}}_k^\dagger G^\dagger G {\mathfrak {b}}_k\lesssim N^{5\kappa -1}\! \left( \widetilde{{\mathcal {N}}}+1\right) ^2 \! \left( \sum _k e_k {\mathfrak {b}}_k^\dagger {\mathfrak {b}}_k+1\right) . \end{aligned}$$

Regarding the $X_k$ term, note that

$$\begin{aligned} \sum _{k\ne 0}e_k \! \left( \! \sum _p \sqrt{N}f_{p,k}{\mathfrak {b}}^\dagger _{p-k}{\mathfrak {a}}_p \! \right) ^\dagger \! \left( \! \sum _p \sqrt{N}f_{p,k}{\mathfrak {b}}^\dagger _{p-k}{\mathfrak {a}}_p \! \right) \!&\lesssim \! N^\kappa \left( {\mathcal {N}} \! + \! \sum _{\ell \ne 0}{\mathfrak {b}}_\ell ^\dagger {\mathcal {N}}{\mathfrak {b}}_\ell \right) \\  &\! \lesssim \! N^{\frac{5\kappa }{2}} \! \left( \widetilde{{\mathcal {N}}}^2 \! + \! 1\right) , \end{aligned}$$

where we have used $\sum _{k\ne 0}|k|^2 f_{p,k}^2=\sum _{k\ne 0}|k|^2 f_{p,p-k}^2\lesssim N^{\kappa -1 }$ by Lemma 14, and hence

$$\begin{aligned} \sum _{k\ne 0}e_k X_k^\dagger X_k\lesssim N^{\frac{5\kappa }{2}} G^\dagger \left( \widetilde{{\mathcal {N}}}^2 \! + \! 1\right) G\lesssim N^{\frac{11\kappa }{2}-1}\left( \widetilde{{\mathcal {N}}}^4 \! + \! 1\right) . \end{aligned}$$

This concludes the proof of Eq. (66) by Eq. (67). In order to estimate $\sum _\ell e_\ell \Theta _\ell ^\dagger \Theta _\ell$, let us define the operator

$$\begin{aligned} (\Pi )_{jk,mn}:=\frac{1}{4}\delta _{j+k=m+n}e_{j+k}\nu _{j+k}^2 N f_{j+k,j} f_{m+n,m}, \end{aligned}$$

which allows us to write

$$\begin{aligned} \sum _\ell e_\ell \Theta _\ell ^\dagger \Theta _\ell =\sum _{k\ne 0} \Pi _k \! \left( 2{\mathfrak {b}}_k^\dagger {\mathfrak {b}}_k+1\right) \! +\sum _{jk,mn}(\Pi )_{jk,mn}{\mathfrak {b}}_k^\dagger {\mathfrak {b}}_j^\dagger {\mathfrak {b}}_m {\mathfrak {b}}_n. \end{aligned}$$

Note that $|N f_{j+k,j}|\lesssim \frac{N^\kappa }{|j|^2+|k|^2}$ by Lemma 1 and $e_k\lesssim N^{\frac{\kappa }{2}}|k|^2$, and therefore

$$\begin{aligned} |\Pi _k|\lesssim N^{\frac{5\kappa }{2}-1}\sum _{j\ne -k}\frac{|j+k|\nu _{j+k}^2}{ \left( |j|^2 + |k|^2\right) ^2}\lesssim N^{\frac{5\kappa }{2}-1}\sum _{j\ne 0}\frac{|j|\nu _{j}^2}{ \left( |j|^2 + |k|^2\right) ^2}. \end{aligned}$$

Again by Lemma 1 we have $0\le \nu _j\lesssim \frac{N^\kappa }{|j|^2}$, which immediately gives us $|\Pi _k|\lesssim N^{\frac{9\kappa }{2}-1}$ and therefore $\pm \sum _{k\ne 0} \Pi _k{\mathfrak {b}}_k^\dagger {\mathfrak {b}}_k\lesssim N^{\frac{9\kappa }{2}-1}\widetilde{{\mathcal {N}}}$. Making use of the fact that

$$\begin{aligned} \sum _{k}\frac{1}{\left( |j|^2 + |k|^2\right) ^2}\lesssim \int _{{\mathbb {R}}^3}\frac{\textrm{d}x}{\left( |j|^2+|x|^2\right) ^2}=\left( \int _{{\mathbb {R}}^3} \frac{\textrm{d}x}{\left( 1+|x|^2\right) ^2}\right) \frac{1}{|j|}\lesssim \frac{1}{|j|}, \end{aligned}$$

we obtain the estimate

$$\begin{aligned} \left| \sum _{0<|j|\le N}\sum _k \frac{|j|\nu _{j}^2}{ \left( |j|^2 + |k|^2\right) ^2}\right| \lesssim N^{2\kappa }\sum _{0<|j|\le N}|j|^{-3}\lesssim N^{2\kappa }\log N . \end{aligned}$$

Consequently, we derive the estimate

$$\begin{aligned} \sum _{k\ne 0} \left| \Pi _k\right|&\lesssim N^{\frac{9\kappa }{2}-1}\log N+N^{\frac{5\kappa }{2}-1}\left| \sum _{|j|>N}\sum _k \frac{|j|\nu _{j}^2}{ \left( |j|^2 + |k|^2\right) ^2}\right| \\&\lesssim N^{\frac{9\kappa }{2}-1}\log N+N^{\frac{5\kappa }{2}-1}\sum _{|j|>N}|j| \nu _j^2, \end{aligned}$$

and we furthermore have by Lemma 14

$$\begin{aligned} \sum _{|j|>N}|j| \nu _j^2\le N^{\frac{5\kappa }{2}-2}\sum _{|j|>0}|j|^2 \nu _j^2\lesssim N^{\frac{7\kappa }{2}-1}\lesssim N^{\frac{9\kappa }{2}-1}\log N. \end{aligned}$$

Finally, we have $\pm \Pi \lesssim N^{\frac{9\kappa }{2}-1}(-\Delta _2)$ and therefore we conclude that

$$\begin{aligned} \pm \sum _{jk,mn}(\Pi )_{jk,mn}{\mathfrak {b}}_k^\dagger {\mathfrak {b}}_j^\dagger {\mathfrak {b}}_m {\mathfrak {b}}_n\lesssim N^{\frac{9\kappa }{2}-1} \! \left( \widetilde{{\mathcal {N}}}+1\right) \left( \sum _k e_k {\mathfrak {b}}_k^\dagger {\mathfrak {b}}_k+1\right) . \end{aligned}$$

$$\square $$

### Annihilation of $\psi _{j k}$

Using the definition of $f_{j,k}$ in Eq. (37) and introducing $\chi :=\left( a_0^\dagger \frac{1}{\sqrt{a_0 a_0^\dagger }}\right) ^2$, we can rewrite

68 $$\begin{aligned} \chi \psi _{j k}\Psi =\left( {\mathfrak {b}}_j{\mathfrak {b}}_k + \sqrt{N}f_{k+j,k}{\mathfrak {a}}_{k+j}+\epsilon _{j k}\right) \Psi \end{aligned}$$

for states $\Psi \in {\mathcal {F}}^{+}_{N-2}$ and $j,k\ne 0$, with

$$\begin{aligned} \epsilon _{j k}&:= \delta _{j+k=0}\! \left( w_k-\gamma _k \nu _k \right) +(\gamma _j \gamma _k-1){\mathfrak {b}}_j {\mathfrak {b}}_k-\nu _j \gamma _k {\mathfrak {b}}_{-j}^\dagger {\mathfrak {b}}_k-\nu _k \gamma _j {\mathfrak {b}}_{-k}^\dagger {\mathfrak {b}}_j+\nu _k \nu _j{\mathfrak {b}}_{-k}^\dagger {\mathfrak {b}}_j^\dagger \\&+ \delta _{j+k=0}w_k\left[ \chi \frac{a_0^2}{N}-1\right] +\sum _{p\ne 0}\left( \sqrt{a_0^\dagger a_0-1}-\sqrt{N}\right) f_{p,k}{\mathfrak {a}}_k. \end{aligned}$$

As the following Lemma 9 demonstrates, the operators $\epsilon _{j k}$ can be regarded as being small.

#### Lemma 9

We have the estimate $\sum _{j k, mn\ne 0}\left( V_{N^{1-\kappa }}\right) _{jk,mn}\epsilon _{jk}^\dagger \epsilon _{mn}\lesssim N^{4\kappa -1}\! \left( \widetilde{{\mathcal {N}}}^2+1\right)$.

#### Proof

Due to the sign of $V_{N^{1-\kappa }}$ it is enough to verify the statement for each term in the definition of $\epsilon _{j k}$ individually. Regarding the term $\delta _{j+k=0}\! \left( w_k-\gamma _k \nu _k \right)$ note that

$$\begin{aligned} |w_k-\gamma _k \nu _k|\lesssim \frac{N^\kappa }{|k|^2}\mathbbm {1}\! \left( |k|\le N^{\frac{\kappa }{2}}\right) +\frac{N^{2\kappa }}{|k|^4}\mathbbm {1}\! \left( |k|> N^{\frac{\kappa }{2}}\right) , \end{aligned}$$

and consequently

$$\begin{aligned} \sum _{j k\ne 0}\left( V_{N^{1-\kappa }}\right) _{(-k)k,(-j)j}|w_k-\gamma _k \nu _k||w_j-\gamma _j \nu _j|\lesssim N^{4\kappa -1}. \end{aligned}$$

For the next term $(\gamma _j \gamma _k-1){\mathfrak {b}}_j {\mathfrak {b}}_k$, note that the operator

$$\begin{aligned} A_{jk,mn}:=\left( V_{N^{1-\kappa }}\right) _{jk,mn}(\gamma _j \gamma _k-1)(\gamma _m \gamma _n-1) \end{aligned}$$

has an operator norm bounded by $CN^{\kappa -1}\sup _T \sum _p (\gamma _p \gamma _{p+T}-1)^2$, which follows from the weighted Schur test, see for example the proof of Lemma 6. Since $\sum _p (\gamma _p \gamma _{p+T}-1)^2\lesssim N^{\frac{3\kappa }{2}}$, uniformly in *T*, we obtain

$$\begin{aligned} \sum _{j k, mn\ne 0}\left( A\right) _{jk,mn}{\mathfrak {b}}_k^\dagger {\mathfrak {b}}_j^\dagger {\mathfrak {b}}_m {\mathfrak {b}}_n\lesssim N^{\frac{5\kappa }{2}-1}\left( \widetilde{{\mathcal {N}}}+1\right) ^2. \end{aligned}$$

Coming to the next term $\nu _j \gamma _k {\mathfrak {b}}_{-j}^\dagger {\mathfrak {b}}_k$ we first notice that

69 $$\begin{aligned}&\sum _{j k, mn\ne 0}\left( V_{N^{1-\kappa }}\right) _{jk,mn}\! \left( \nu _j \gamma _k {\mathfrak {b}}_{-j}^\dagger {\mathfrak {b}}_k \right) ^\dagger \! \left( \nu _m \gamma _n {\mathfrak {b}}_{-m}^\dagger {\mathfrak {b}}_n\right) =\sum _{q}\mu _q {\mathfrak {b}}_q^\dagger {\mathfrak {b}}_q\nonumber \\&\quad +\sum _{j k, mn\ne 0}\left( A'\right) _{jk,mn}{\mathfrak {b}}_k^\dagger {\mathfrak {b}}_j^\dagger {\mathfrak {b}}_m {\mathfrak {b}}_n, \end{aligned}$$

where we define

$$\begin{aligned} \mu _q&:= \gamma _q^2\sum _p \left( V_{N^{1-\kappa }}\right) _{p q,p q}\nu _p^2 ,\\ \left( A'\right) _{jk,mn}&:= \left( V_{N^{1-\kappa }}\right) _{jn,mk}\gamma _j \nu _k \gamma _m \nu _n. \end{aligned}$$

Since $|\mu _q|\lesssim N^{\frac{7\kappa }{2}-1}$ and $\Vert A'\Vert \lesssim N^{\frac{7\kappa }{2}-1}$, the term in Eq. (69) is bounded by $N^{\frac{7\kappa }{2} -1}\! \left( \widetilde{{\mathcal {N}}}^2+1\right)$. Note that the terms $\nu _k \gamma _j {\mathfrak {b}}_{-k}^\dagger {\mathfrak {b}}_j$ and $\nu _k \nu _j{\mathfrak {b}}_{-k}^\dagger {\mathfrak {b}}_j^\dagger$ can be analysed analogously. Coming to the term $\delta _{j+k=0}w_k\left[ \chi \frac{a_0^2}{N}-1\right]$ we observe that

$$\begin{aligned} \left[ \chi \frac{a_0^2}{N}-1\right] ^\dagger \left[ \chi \frac{a_0^2}{N}-1\right] \lesssim N^{-2}\! \left( {\mathcal {N}}+1\right) ^2\lesssim N^{3\kappa -2}\! \left( \widetilde{{\mathcal {N}}}+1\right) ^2. \end{aligned}$$

Therefore, $$\sum _{j k\ne 0}\left( V_{N^{1-\kappa }}\right) _{(-k)k,(-j)j}w_k w_j\left[ \chi \frac{a_0^2}{N}-1\right] ^\dagger \left[ \chi \frac{a_0^2}{N}-1\right] \lesssim N^{3\kappa -2}\mu \! \left( \widetilde{{\mathcal {N}}}+1\right) ^2$$ with

$$\begin{aligned} \mu :&= \! \! \! \sum _{j k\ne 0} \! \! \left( V_{N^{1-\kappa }}\right) _{(-k)k,(-j)j}w_k w_j \! = \! N^2 \! \! {\langle {V_{N^{1-\kappa }},R V_{N^{1-\kappa }} R V_{N^{1-\kappa }}}\rangle }\\  &\! \le \! N^2 {\langle {V_{N^{1-\kappa }},R V_{N^{1-\kappa }}}\rangle } \! \lesssim \! N^{1+\kappa }. \end{aligned}$$

The final contribution $\sum _{p\ne 0}\left( \sqrt{a_0^\dagger a_0-1}-\sqrt{N}\right) f_{p,k}{\mathfrak {a}}_k$ can be dealt with analogously. $\square$

In order to get rid of the term $\sqrt{N}f_{k+j,k}{\mathfrak {a}}_{k+j}$ in Eq. (68), let us again use the operator *G* introduced above Eq. (65), which allows us to write

70 $$\begin{aligned} \chi \psi _{j k} \! \left( 1 \! - \! G\right) \Psi =\left( {\mathfrak {b}}_j{\mathfrak {b}}_k +\epsilon _{j,k} \left( 1 \! - \! G\right) - {\widetilde{\epsilon }}_{j,k}\right) \Psi \end{aligned}$$

for states $\Psi \in {\mathcal {F}}^{+}_{N-3}$, where we have used $(1-G){\mathcal {F}}^{+}_{N-3}\subset {\mathcal {F}}^{+}_{N-2}$, with

$$\begin{aligned} {\tilde{\epsilon }}_{j,k}: \! \!&= \! \! \frac{1}{2}\sum _{p,q} \! \sqrt{N}f_{p,q}{\mathfrak {b}}_q^\dagger {\mathfrak {b}}_{p-q}^\dagger {\mathfrak {b}}_j {\mathfrak {b}}_k {\mathfrak {a}}_p \! + \! \sum _p \! \sqrt{N}f_{p,k} {\mathfrak {b}}_{p-k}^\dagger {\mathfrak {b}}_j {\mathfrak {a}}_p \! \\&\quad + \! \sum _p \! \sqrt{N}f_{p,j} {\mathfrak {b}}_{p-j}^\dagger {\mathfrak {b}}_k {\mathfrak {a}}_p \! + \! \sqrt{N}f_{k+j,k}{\mathfrak {a}}_{k+j} G. \end{aligned}$$

#### Lemma 10

Let ${\mathbb {K}}:=\sum _k e_k {\mathfrak {b}}_k^\dagger {\mathfrak {b}}_k+\frac{1}{2}\sum _{j k,mn\ne 0} \left( V_{N^{1-\kappa }}\right) _{j k,mn} {\mathfrak {b}}_k^\dagger {\mathfrak {b}}_j^\dagger {\mathfrak {b}}_m {\mathfrak {b}}_n$. Then we have

$$\begin{aligned} \sum _{j k, mn\ne 0}\left( V_{N^{1-\kappa }}\right) _{jk,mn}{\tilde{\epsilon }}_{jk}^\dagger {\tilde{\epsilon }}_{mn}\lesssim N^{\frac{11\kappa }{2}-1}\! \left( \widetilde{{\mathcal {N}}}^4+1\right) \! \! \left( {\mathbb {K}}+1\right) . \end{aligned}$$

#### Proof

Note that we can carry out the estimate for each term in ${\tilde{\epsilon }}_{jk}$ individually due to the positivity of *V*. Starting with the analysis of $\sqrt{N}f_{k+j,k}{\mathfrak {a}}_{k+j} G$, we obtain

$$\begin{aligned} \sum _{j k, mn\ne 0} \left( V_{N^{1-\kappa }}\right) _{jk,mn}\left( \sqrt{N}f_{k+j,k}{\mathfrak {a}}_{k+j} G\right) ^\dagger \left( \sqrt{N}f_{n+m,n}{\mathfrak {a}}_{n+m} G\right) \lesssim \sum _{p\ne 0}\mu _p G^\dagger a_p^\dagger a_p G \end{aligned}$$

with $\mu _p:=N\underset{j+k=p}{\sum _{j k, mn\ne 0}}\left( V_{N^{1-\kappa }}\right) _{jk,mn}\overline{f_{k+j,k}}f_{n+m,n}$. Note that

$$\begin{aligned} \mu _p=N{\langle {e^{ipx}V_{N^{1-\kappa }},R V_{N^{1-\kappa }} R e^{ipx}V_{N^{1-\kappa }}}\rangle }\le N{\langle {e^{ipx}V_{N^{1-\kappa }},R e^{ipx}V_{N^{1-\kappa }}}\rangle }\lesssim N^{\kappa }, \end{aligned}$$

and therefore

$$\begin{aligned} \sum _{p\ne 0}\mu _p G^\dagger a_p^\dagger a_p G\lesssim N^{\kappa }G^\dagger {\mathcal {N}} G\lesssim N^{\frac{5\kappa }{2}}G^\dagger \left( \widetilde{{\mathcal {N}}}+1\right) G\lesssim N^{\frac{11\kappa }{2}-1}\left( \widetilde{{\mathcal {N}}}^3+1\right) . \end{aligned}$$

Regarding the other terms, we are going to expand them further according to

$$\begin{aligned} {\mathfrak {a}}_k=\gamma _k {\mathfrak {b}}_k-\nu _k {\mathfrak {b}}_{-k}^\dagger , \end{aligned}$$

and bringing them in normal order, e.g.

$$\begin{aligned} \sum _p \sqrt{N}f_{p,k} {\mathfrak {b}}_{p-k}^\dagger {\mathfrak {b}}_j {\mathfrak {a}}_p&=\sum _p \gamma _p \sqrt{N}f_{p,k} {\mathfrak {b}}_{p-k}^\dagger {\mathfrak {b}}_j {\mathfrak {b}}_p-\sum _p \nu _p \sqrt{N}f_{p,k} {\mathfrak {b}}_{p-k}^\dagger {\mathfrak {b}}_j {\mathfrak {b}}_p^\dagger \\&=\sum _p \gamma _p \sqrt{N}f_{p,k} {\mathfrak {b}}_{p-k}^\dagger {\mathfrak {b}}_j {\mathfrak {b}}_p\\&\quad -\sum _p \nu _p \sqrt{N}f_{p,k} {\mathfrak {b}}_{p-k}^\dagger {\mathfrak {b}}_p^\dagger {\mathfrak {b}}_j -\nu _j \sqrt{N}f_{j,k} {\mathfrak {b}}_{j-k}^\dagger . \end{aligned}$$

Let us illustrate how to proceed, by analysing the $\nu _j \sqrt{N}f_{j,k} {\mathfrak {b}}_{j-k}^\dagger$ term in more detail

$$\begin{aligned}&\sum _{j k, mn\ne 0}\left( V_{N^{1-\kappa }}\right) _{jk,mn}\left( \nu _j \sqrt{N}f_{j,k} {\mathfrak {b}}_{j-k}^\dagger \right) ^\dagger \left( \nu _m \sqrt{N}f_{m,n} {\mathfrak {b}}_{m-n}^\dagger \right) \\&\quad \le 2 \sum _{r=1}^2\underset{j,m\in I_r}{\sum _{j k, mn\ne 0:}}\left( V_{N^{1-\kappa }}\right) _{jk,mn}\left( \nu _j \sqrt{N}f_{j,k} {\mathfrak {b}}_{j-k}^\dagger \right) ^\dagger \left( \nu _m \sqrt{N}f_{m,n} {\mathfrak {b}}_{m-n}^\dagger \right) , \end{aligned}$$

where $I_1:=\{j: |j|\le N\}$ and $I_2:=\{j: |j|> N\}$. Regarding the $r=2$ term, let us define the operator *B* in coordinates as

$$\begin{aligned} (B)_{jk,mn}:=\frac{\mathbbm {1}_{I_2}(j)}{|j|^{\frac{1+\delta }{2}}|k|}\frac{\mathbbm {1}_{I_2}(m)}{|m|^{\frac{1+\delta }{2}}|n|}\left( V_{N^{1-\kappa }}\right) _{jk,mn}, \end{aligned}$$

for $\delta >0$, and apply the weighted Schur test in order to estimate the operator norm

$$\begin{aligned} \Vert B\Vert \le \sup _{j,k}\sum _{|m|>N,n\ne 0}\left| \frac{1}{|m|^{1+\delta }|n|^2}\left( V_{N^{1-\kappa }}\right) _{jk,mn}\right|&\lesssim N^{\kappa -1}\sup _{z}\sum _{|m|>N}\left| \frac{1}{|m|^{1+\delta }|z-m|^2}\right| \\  &\lesssim N^{\kappa -\delta -1}. \end{aligned}$$

Consequently,

71 $$\begin{aligned}&\underset{j,m\in I_2}{\sum _{j k, mn\ne 0:}}\left( V_{N^{1-\kappa }}\right) _{jk,mn}\left( \nu _j \sqrt{N}f_{j,k} {\mathfrak {b}}_{j-k}^\dagger \right) ^\dagger \left( \nu _m \sqrt{N}f_{m,n} {\mathfrak {b}}_{m-n}^\dagger \right) \nonumber \\&\quad = \underset{j,m\in I_2}{\sum _{j k, mn\ne 0:}}\left( B\right) _{jk,mn}\left( |j|^{\frac{1+\delta }{2}}|k|\nu _j \sqrt{N}f_{j,k} {\mathfrak {b}}_{j-k}^\dagger \right) ^\dagger \left( |m|^{\frac{1+\delta }{2}}|n|\nu _m \sqrt{N}f_{m,n} {\mathfrak {b}}_{m-n}^\dagger \right) \nonumber \\&\quad \lesssim N^{\kappa -\delta -1} \! \sum _{jk}|k|^{2}|j|^{1+\delta }\left( \nu _j \sqrt{N}f_{j,k} {\mathfrak {b}}_{j-k}^\dagger \right) ^\dagger \! \left( \nu _j \sqrt{N}f_{j,k} {\mathfrak {b}}_{j-k}^\dagger \right) \nonumber \\&\quad \lesssim \! N^{5\kappa -2}\widetilde{{\mathcal {N}}} \! + \! N^{2\kappa -\delta -1}\sum _j |j|^{1+\delta }\nu _j^2 \end{aligned}$$

for $0<\delta <1$, where we have used $\sum _{k\ne 0}|k|^2 f_{j,k}^2=\sum _{k\ne 0}|k|^2 f_{j,j-k}^2\lesssim N^{\kappa -1 }$ by Lemma 14. Furthermore, $\sum _j |j|^{1+\delta }\nu _j^2\lesssim N^{2\kappa +\delta }$, again by Lemma 14, which concludes the argument for $r=2$. The corresponding estimate for $r=1$ follows from the bounds

$$\begin{aligned} |N f_{j+k,j}| \lesssim \frac{N^\kappa }{|j|^2+|k|^2} ,\ \ \ \ \ \ \ 0\le \nu _j \lesssim \frac{N^\kappa }{|j|^2}, \end{aligned}$$

see Lemma 1. Regarding the final term in the definition of ${\tilde{\epsilon }}_{jk}$, we note that

$$\begin{aligned} A_{p,p'}:=\left( \sum _{q'}\sqrt{N}\gamma _{p'} f_{p',q'}{\mathfrak {b}}_{q'}^\dagger {\mathfrak {b}}_{p'-q'}^\dagger \right) ^\dagger \left( \sum _q \sqrt{N}\gamma _p f_{p,q}{\mathfrak {b}}_q^\dagger {\mathfrak {b}}_{p-q}^\dagger \right) \end{aligned}$$

satisfies $A\lesssim N^{\frac{5\kappa }{2}-1} \! \left( \widetilde{{\mathcal {N}}}+1\right) ^2$, and therefore

$$\begin{aligned}&\sum _{j k, mn\ne 0}\left( V_{N^{1-\kappa }}\right) _{jk,mn}\left( \sum _{p,q} \! \sqrt{N}f_{p,q}{\mathfrak {b}}_q^\dagger {\mathfrak {b}}_{p-q}^\dagger {\mathfrak {b}}_j {\mathfrak {b}}_k {\mathfrak {a}}_p\right) ^\dagger \left( \sum _{p,q} \! \sqrt{N}f_{p,q}{\mathfrak {b}}_q^\dagger {\mathfrak {b}}_{p-q}^\dagger {\mathfrak {b}}_m {\mathfrak {b}}_n {\mathfrak {a}}_p\right) \\&\quad \lesssim N^{\frac{5\kappa }{2}-1} \sum _{j k, mn\ne 0}\left( V_{N^{1-\kappa }}\right) _{jk,mn} {\mathfrak {b}}_k^\dagger {\mathfrak {b}}_j^\dagger \! \left( \widetilde{{\mathcal {N}}}+1\right) ^2 \! {\mathfrak {b}}_m {\mathfrak {b}}_n + N^{3\kappa -1}\widetilde{{\mathcal {N}}}\\&\quad \lesssim N^{3\kappa -1} \! \left( \widetilde{{\mathcal {N}}}+1\right) ^2 {\mathbb {K}}. \end{aligned}$$

$$\square $$

#### Corollary 3

Let $\Xi _{jk}:=\psi _{j k}(1 \! - \! G)-{\mathfrak {b}}_j {\mathfrak {b}}_k$. Then there exists a $C>0$ such that for $\kappa <\frac{2}{11}$

72 $$\begin{aligned} \sum _{j k,mn\ne 0} \! \! \left( V_{N^{1-\kappa }}\right) _{j k,mn}\Xi _{jk}^\dagger \, \Xi _{mn}\Big |_{{\mathcal {F}}^{+}_{N-3}} \! \le \! C N^{\frac{11\kappa }{2}-1} \! \left( \widetilde{{\mathcal {N}}} \! + \! 1\right) ^4 \! \left( {\mathbb {K}} \! + \! 1\right) \Big |_{{\mathcal {F}}^{+}_{N-3}}. \end{aligned}$$

#### Proof

By Eq. (70) we have the bound

$$\begin{aligned} \sum _{j k,mn\ne 0} \! \! \left( V_{N^{1-\kappa }}\right) _{j k,mn}\Xi _{jk}^\dagger \, Xi_{mn}\Big |_{{\mathcal {F}}^{+}_{N-3}}&\lesssim \sum _{j k, mn\ne 0}\left( V_{N^{1-\kappa }}\right) _{jk,mn}(1\! - \! G)^\dagger \epsilon _{jk}^\dagger \epsilon _{mn}(1\! - \! G)\Big |_{{\mathcal {F}}^{+}_{N-3}}\\  &\quad + \sum _{j k, mn\ne 0}\left( V_{N^{1-\kappa }}\right) _{jk,mn}{\tilde{\epsilon }}_{jk}^\dagger {\tilde{\epsilon }}_{mn}\Big |_{{\mathcal {F}}^{+}_{N-3}}. \end{aligned}$$

Note that by Lemma 9

$$\begin{aligned} \sum _{j k, mn\ne 0} \! \! \! \! \! \! \left( V_{N^{1-\kappa }}\right) _{jk,mn}(1\! - \! G)^\dagger \! \epsilon _{jk}^\dagger \epsilon _{mn}(1\! - \! G) \!&\lesssim \! N^{\frac{7\kappa }{2}-1}\! (1\! - \! G)^\dagger \! \left( \widetilde{{\mathcal {N}}}^2+1\right) \! (1\! - \! G) \!\\  &\lesssim N^{\frac{7\kappa }{2}-1} \! \left( \widetilde{{\mathcal {N}}}^5 \! + \! 1\right) . \end{aligned}$$

In combination with Lemma 10 this concludes the proof. $\square$

### Proof of Theorem 3

Regarding the verification of Theorem 3, let us define for $\delta >0$ the trial states

$$\begin{aligned} \Psi _d:=Z_d^{-1}(1 \! - \! G)\chi _\delta \, \Gamma _d, \end{aligned}$$

where we define the operator

$$\begin{aligned} \chi _\delta :=\mathbbm {1} \! \left( {\mathcal {N}}\le N^{\frac{3\kappa }{2}+\delta }\right) =\mathbbm {1} \! \left( \sum _{k\ne 0}{\mathfrak {a}}^\dagger _k {\mathfrak {a}}_k\le N^{\frac{3\kappa }{2}+\delta }\right) , \end{aligned}$$

and $\Gamma _d$ is the *d*-th eigenvector of the operator $\sum _k e_k{\mathfrak {b}}_k^\dagger {\mathfrak {b}}_k$, i.e. $\Gamma _d$ is the eigenvector of $\sum _k e_k{\mathfrak {b}}_k^\dagger {\mathfrak {b}}_k$ corresponding to the eigenvalue ${\widetilde{\lambda }}_{N,\kappa }^{(d)}$ defined above Eq. (60), and $Z_j$ is a normalization constant. Note that $\Psi _d\in {\mathfrak {h}}$ is an element of ${\mathcal {F}}^{+}_{N-1}\subset L^2_\textrm{sym} \! \left( \Lambda ^N\right)$ for *N* large enough, and therefore an appropriate trial state. The following Lemma 11 demonstrates that the eigenstates $\Gamma _d$ are already in the range of the projections $\chi _\delta$ up to an error that decays faster than any power in $\frac{1}{N}$.

#### Lemma 11

For any $\lambda \in {\mathbb {R}}$, $m\in {\mathbb {N}}$ and $\delta >0$, there exists a constant $C>0$ such that

73 $$\begin{aligned} \bigg \langle (1 \! - \! \chi _\delta ) \Gamma _d,\Big (\sum _{k\ne 0}{\mathfrak {b}}^\dagger _k {\mathfrak {b}}_k\Big )^m {\mathbb {K}} \, (1 \! - \! \chi _\delta ) \Gamma _d\bigg \rangle \le C N^{-\lambda }, \end{aligned}$$

where ${\mathbb {K}}$ is defined in Lemma 10. Furthermore, $|Z_d-1|\le C' N^{\frac{7\kappa }{4}-\frac{1}{2}}$ for a suitable $C'$.

#### Proof

In the following let $\widetilde{{\mathbb {K}}}:=\sum _k |k|^2 {\mathfrak {a}}_k^\dagger {\mathfrak {a}}_k+\frac{1}{2}\sum _{k \ell , m n}(V_{N^{1-\kappa }})_{k \ell , m n} {\mathfrak {a}}^\dagger _k {\mathfrak {a}}^\dagger _\ell {\mathfrak {a}}_m {\mathfrak {a}}_n$ and note

$$\begin{aligned} \Big (\sum _{k\ne 0}{\mathfrak {b}}^\dagger _k {\mathfrak {b}}_k\Big )^m {\mathbb {K}} \lesssim N^{\gamma _m} \Big (\sum _{k\ne 0}{\mathfrak {a}}^\dagger _k {\mathfrak {a}}_k+1\Big )^{m} \left( \widetilde{{\mathbb {K}}}+1\right) =:N^{\gamma _m} {\mathcal {M}} \end{aligned}$$

by Lemma 16, with $\gamma _m:=\frac{3(m+1)\kappa }{2}+1$. Since $1-\chi _\delta \le N^{-\ell \left( \frac{3\kappa }{2}+\delta \right) }\Big (\sum _{k\ne 0}{\mathfrak {a}}^\dagger _k {\mathfrak {a}}_k\Big )^\ell$ for any $\ell$, and both $\chi _\delta$ and ${\mathcal {N}}$ commute with the non-negative operator ${\mathcal {M}}$, we have

$$\begin{aligned} (1 \! - \! \chi _\delta ) {\mathcal {M}} (1 \! - \! \chi _\delta ) \lesssim N^{-\ell \left( 3\kappa +2\delta \right) } \Big (\sum _{k\ne 0}{\mathfrak {a}}^\dagger _k {\mathfrak {a}}_k+1\Big )^{2\ell }{\mathcal {M}} , \end{aligned}$$

and therefore $\bigg \langle (1 \! - \! \chi _\delta ) \Gamma _d,\Big (\sum _{k\ne 0}{\mathfrak {b}}^\dagger _k {\mathfrak {b}}_k\Big )^m {\mathbb {K}} \, (1 \! - \! \chi _\delta ) \Gamma _d\bigg \rangle$ is bounded from above by

$$\begin{aligned}&N^{\gamma _m-\ell \left( 3\kappa +2\delta \right) } \Big \langle \Gamma _d,\Big (\sum _{k\ne 0}{\mathfrak {a}}^\dagger _k {\mathfrak {a}}_k+1\Big )^{2\ell +m} \left( \widetilde{{\mathbb {K}}}+1\right) \Gamma _d\Big \rangle \\&\quad \lesssim N^{2\gamma _m-2\ell \delta }\bigg \langle \Gamma _d,\Big (\sum _{k\ne 0}{\mathfrak {b}}^\dagger _k {\mathfrak {b}}_k+1\Big )^{2\ell +m} \left( {\mathbb {K}}+1\right) \Gamma _d\bigg \rangle , \end{aligned}$$

where we used Lemma 16 and we observe that $\bigg \langle \Gamma _d,\Big (\sum _{k\ne 0}{\mathfrak {b}}^\dagger _k {\mathfrak {b}}_k+1\Big )^{2\ell +m+1} \left( {\mathbb {K}}+1\right) \Gamma _d\bigg \rangle$ is finite since $\Gamma _d$ is an eigenstate. Choosing $2\ell \delta \ge 2\gamma _m+\lambda$ concludes the proof of Eq. (73). Regarding the estimate on $Z_d$, note that $Z_d^2=\Vert \chi _\delta \Gamma _d\Vert ^2-2\mathfrak {Re}{\langle {\chi _\delta \Gamma _d,G\chi _\delta \Gamma _d}\rangle }+{\langle {\chi _\delta \Gamma _d,G^\dagger G\chi _\delta \Gamma _d}\rangle }$. Making use of the operator inequality $G^\dagger G \lesssim N^{\frac{7\kappa }{2}-1}\! \left( \widetilde{{\mathcal {N}}}+1\right) ^2$ therefore yields

$$\begin{aligned} |Z_d^2-1|\le 1-\Vert \chi _\delta \Gamma _d\Vert ^2+N^{\frac{7\kappa }{4}-\frac{1}{2}}\! \left( \Vert \chi _\delta \Gamma _d\Vert ^2+{\langle {\chi _\delta \Gamma _d,\! \left( \widetilde{{\mathcal {N}}}+1\right) ^2 \! \chi _\delta \Gamma _d}\rangle }\right) . \end{aligned}$$

By Eq. (73) we know that $1-\Vert \chi _\delta \Gamma _d\Vert ^2\lesssim N^{-\lambda }$ for any $\lambda >0$ and

$$\begin{aligned} {\langle {\chi _\delta \Gamma _d,\! \left( \widetilde{{\mathcal {N}}}+1\right) ^2 \! \chi _\delta \Gamma _d}\rangle }\lesssim {\langle { \Gamma _d,\! \big (\sum _{k\ne 0}{\mathfrak {b}}^\dagger _k {\mathfrak {b}}_k+1\big )^2 \Gamma _d}\rangle }+N^{-\lambda }\lesssim 1, \end{aligned}$$

which concludes the proof. $\square$

In order to compute the energy of $\Psi _d$, we are first going to combine the results from Sects. 5.1 and 5.2, as well as the results in Sect. 4, yielding the following Theorem 4.

#### Theorem 4

For $\kappa <\frac{2}{13}$, let us define the operator $W_\textrm{log}:=-\frac{1}{2}\sum _{p,k}e_k \nu _k \sqrt{N}f_{-k,p} {\mathfrak {b}}^\dagger _{-(k+p)}{\mathfrak {b}}_k^\dagger {\mathfrak {b}}^\dagger _p$ and the (*N* dependent) constant $C_\textrm{log}:=\sum _{k} \Pi _k-D_\textrm{log}$, where $\Pi _k$ is defined in Corollary 2, $D_\textrm{log}:=\sum _{k,k'}\nu _{k'}\nu _k \gamma _{k'}\gamma _k \Lambda _{k (-k), k' (-k')}$ and $\Lambda$ is introduced above Eq. (46). Then we have

74 $$\begin{aligned} (1 \! - \! G)^\dagger H_{N,\kappa }' (1 \! - \! G)\Big |_{{\mathcal {F}}^{+}_{N-3}}=\left( C_\textrm{log}+ {\mathbb {K}} + W_\textrm{log}+W_\textrm{log}^\dagger + X\right) \Big |_{{\mathcal {F}}^{+}_{N-3}}, \end{aligned}$$

where *X* is an operator satisfying

$$\begin{aligned} \pm X\Big |_{{\mathcal {F}}^+_{rN}}\le C_r N^{\frac{11\kappa }{4}-\frac{1}{2}} \! \left( \widetilde{{\mathcal {N}}}+1\right) ^6 \! \Big ({\mathbb {K}}+1\Big )\Big |_{{\mathcal {F}}^+_{rN}} \end{aligned}$$

for $0<r<1$. Furthermore, $\big | \! \left\langle \chi _\delta \Gamma _1,X \, \chi _\delta \Gamma _1\right\rangle \! \big | \lesssim \frac{\sqrt{log N}}{N}$ as well as $\big | \! \left\langle \chi _\delta \Gamma _1,W_\textrm{log} \, \chi _\delta \Gamma _1\right\rangle \! \big | \lesssim \frac{1}{N}$ in the case $\kappa =0$, and $|D_\textrm{log}|\lesssim N^{\frac{9\kappa }{2}}\frac{\log N}{N}$.

#### Proof

We first derive the estimate on $D_\textrm{log}$. By Lemma 1 we have the estimates $|\nu _{k'}|\lesssim \frac{N^{\kappa }}{|k'|^2}$ and $|\Lambda _{k (-k), k' (-k')}|\lesssim \frac{N^{2\kappa -1}}{|k|^2+|k'|^2}$. Combining this with $|\gamma _k|\lesssim N^{\frac{\kappa }{4}}$ yields

$$\begin{aligned} |D_\textrm{log}| \!&\lesssim \! \frac{N^{\frac{7\kappa }{2}}}{N}\sum _{k,k'}|\nu _k|\frac{1}{|k'|^2 (|k|^2 \! + \! |k'|^2)} \! \lesssim \! \frac{N^{\frac{7\kappa }{2}}}{N}\sum _{k}|\nu _k|\int _{{\mathbb {R}}^3}\frac{\textrm{d}k'}{|k'|^2 (|k|^2 \! + \! |k'|^2)} \! \\&= \! \frac{N^{\frac{7\kappa }{2}}}{N}\mu \sum _{k}|k|^{-1} |\nu _k| \end{aligned}$$

with $\mu :=\int _{{\mathbb {R}}^3}\frac{\textrm{d}k'}{|k'|^2 (1+|k'|^2)}<\infty$. Furthermore, note that we have

$$\begin{aligned} \frac{N^{\frac{7\kappa }{2}}}{N}\mu \sum _{0<|k|\le N}|k|^{-1} |\nu _k|\lesssim \frac{N^{\frac{9\kappa }{2}}}{N}\sum _{0<|k|\le N}|k|^{-3}\lesssim \frac{N^{\frac{9\kappa }{2}}}{N}\int _{1}^N \frac{\textrm{d}r}{r}=N^{\frac{9\kappa }{2}}\frac{\log N}{N}, \end{aligned}$$

as well as $\sum _{|k|>N}|k|^{-1} |\nu _k|\le \sqrt{\sum _{|k|>N}|k|^{-4}}\sqrt{\sum _{k}|k|^{2}|\nu _k|^2}\lesssim N^{\frac{\kappa }{2}}$ by Lemma 14, which provides the desired bound on $D_\textrm{log}$. In order to achieve the decomposition in Eq. (74), recall the definition of $\Xi _k$ and $\Xi _{jk}$ from Corollary 2 and Corollary 3. By Eq. (63) we can write

$$\begin{aligned}&(1 - G)^\dagger \left( H_{N,\kappa }' +\sum _{i=1}^4 {\mathcal {E}}_i\right) (1 - G)\\&\quad = \sum _{k\ne 0}e_k \left( {\mathfrak {b}}_k-\Theta _k+\Xi _k\right) ^\dagger \left( {\mathfrak {b}}_k-\Theta _k+\Xi _k\right) \\&\qquad +\frac{1}{2}\sum _{j k,mn\ne 0} \! \! \left( V_{N^{1-\kappa }}\right) _{j k,mn}\left( {\mathfrak {b}}_k{\mathfrak {b}}_j+\Xi _{jk}\right) ^\dagger \left( {\mathfrak {b}}_m{\mathfrak {b}}_n+\Xi _{mn}\right) . \end{aligned}$$

Recalling the definition of $\Theta _k$ and $\Pi _k$ from Corollary 2, we define

$$\begin{aligned} Y&:= \sum _{k\ne 0}e_k \Xi _k^\dagger \, \Theta _k,\\ {\widetilde{X}}_1&:= \sum _{k\ne 0}e_k \Xi _k^\dagger \, \Xi _k+\frac{1}{2}\sum _{j k,mn\ne 0} \! \! \left( V_{N^{1-\kappa }}\right) _{j k,mn}\Xi _{jk}^\dagger \, \Xi _{mn}\\&\quad +\sum _{k\ne 0}e_k \Theta _k^\dagger \Theta _k- \sum _{k\ne 0}\Pi _k -\! \left( Y \! + \! Y^\dagger \right) ,\\ {\widetilde{X}}_2&:= \sum _{k\ne 0}e_k \Xi _k^\dagger {\mathfrak {b}}_k+\frac{1}{2}\sum _{j k,mn\ne 0} \! \! \left( V_{N^{1-\kappa }}\right) _{j k,mn}\Xi _{jk}^\dagger \, {\mathfrak {b}}_m {\mathfrak {b}}_n, \end{aligned}$$

in order to obtain

75 $$\begin{aligned} (1 - G)^\dagger \left( H_{N,\kappa }' +\sum _{i=1}^4 {\mathcal {E}}_i\right) (1 - G)=\sum _{k\ne 0}\Pi _k +{\mathbb {K}}+ W_\textrm{log}+W_\textrm{log}^\dagger +{\widetilde{X}}_1+{\widetilde{X}}_2+{\widetilde{X}}_2^\dagger , \end{aligned}$$

where we used that

$$\begin{aligned} \sum _{k\ne 0}e_k \left( {\mathfrak {b}}_k \! - \! \Theta _k\right) ^\dagger \left( {\mathfrak {b}}_k \! - \! \Theta _k\right)&+\frac{1}{2}\sum _{j k,mn\ne 0} \! \! \left( V_{N^{1-\kappa }}\right) _{j k,mn}{\mathfrak {b}}_k^\dagger {\mathfrak {b}}_j^\dagger {\mathfrak {b}}_m{\mathfrak {b}}_n=\sum _{k\ne 0}e_k \Theta _k^\dagger \Theta _k \! + \! {\mathbb {K}} \! \\  &\quad + \! W_\textrm{log} \! + \! W_\textrm{log}^\dagger . \end{aligned}$$

Let us furthermore introduce $X:=X_1+X_2$ with

$$\begin{aligned} X_1&:= {\widetilde{X}}_1+(1 - G)^\dagger \left( {\mathcal {E}}_\textrm{log}+{\mathcal {E}}_\textrm{odd}-\sum _{i=1}^4 {\mathcal {E}}_i\right) (1 - G),\\ X_2&:= {\widetilde{X}}_2+{\widetilde{X}}_2^\dagger + D_\textrm{log}-(1 - G)^\dagger \left( {\mathcal {E}}_\textrm{log}+{\mathcal {E}}_\textrm{odd}\right) (1 - G), \end{aligned}$$

where we define

$$\begin{aligned} {\mathcal {E}}_\textrm{log}&:= \sum _{k \ell , k' \ell '}\Lambda _{k \ell , k' \ell '}\, a_{\ell '}^\dagger a_{k'}^\dagger \frac{a_0^\dagger a_0}{N} a_k a_\ell ,\\ {\mathcal {E}}_{\textrm{odd}}&:= \sum _{i=1}^3\left( \sum _{\ell ,k}\Upsilon ^{(i)}_{\ell ,k} O_i\, a_k^\dagger a_\ell ^\dagger a_{k+\ell }+\mathrm {H.c.}\right) , \end{aligned}$$

with $\Lambda$, $\Upsilon ^{(i)}$ and $O_i$ introduced above Eq. (46). Using Eq. (75) it is easy to check that *X* allows a decomposition of $(1 - G)^\dagger H_{N,\kappa }' (1 - G)$ according to Eq. (74). As a consequence of Corollary 2 and Corollary 3 we obtain

$$\begin{aligned} \pm \left( {\widetilde{X}}_2+{\widetilde{X}}_2^\dagger \right) \big |_{{\mathcal {F}}^+_{N-3}}\lesssim N^{\frac{11\kappa }{4}-\frac{1}{2}} \! \left( \widetilde{{\mathcal {N}}}+1\right) ^6 \! \Big ({\mathbb {K}}+1\Big )\big |_{{\mathcal {F}}^+_{N-3}} , \end{aligned}$$

and by Lemma 7 we have

$$\begin{aligned} \pm (1 - G)^\dagger {\mathcal {E}}_\textrm{odd}(1 - G)\Big |_{{\mathcal {F}}^+_{rN}} \! \! \!&\lesssim N^{\frac{13\kappa }{4}-\frac{1}{2}}(1 - G)^\dagger \left( \widetilde{{\mathcal {N}}}+1\right) (1 - G)\Big |_{{\mathcal {F}}^+_{rN}}\\  &\lesssim N^{\frac{13\kappa }{4}-\frac{1}{2}}\left( \widetilde{{\mathcal {N}}}+1\right) ^3 \Big |_{{\mathcal {F}}^+_{rN}}. \end{aligned}$$

In order to analyse ${\mathcal {E}}_{\textrm{log}}$, define $\alpha _\delta :=\sup _p \left\{ \sum _k |k|^{1-2\delta } \big (\sqrt{N}f_{p,k}\big )^2\right\} \lesssim N^{2\kappa -1}$ and observe

76 $$\begin{aligned}&G^\dagger \! \left( \widetilde{{\mathcal {N}}} \! + \! 1\right) \! \! \left( \sum _k |k|^{1-2\delta } b_k^\dagger b_k \! + \! 1\right) \! G \nonumber \\&\quad \lesssim \alpha _{-\frac{1}{2}} \sum _p {\mathfrak {a}}_p^\dagger \left( \widetilde{{\mathcal {N}}} \! + \! 1\right) ^3 \! \! \left( \sum _k |k|^{1-2\delta } b_k^\dagger b_k \! + \! 1\right) {\mathfrak {a}}_p+ \alpha _\delta \sum _p {\mathfrak {a}}_p^\dagger \left( \widetilde{{\mathcal {N}}} \! + \! 1\right) ^3 \! {\mathfrak {a}}_p\nonumber \\&\quad \lesssim N^{4\kappa -1}\left( \widetilde{{\mathcal {N}}} \! + \! 1\right) ^4 \! \! \left( \sum _k |k|^{1-2\delta } b_k^\dagger b_k \! + \! 1\right) . \end{aligned}$$

By Lemma 6 we therefore have

$$\begin{aligned} \pm (1 - G)^\dagger {\mathcal {E}}_\textrm{log}(1 - G)\lesssim N^{\frac{11\kappa }{2}+3\delta -1}\left( \widetilde{{\mathcal {N}}} \! + \! 1\right) ^4 \! \! \left( \sum _k |k|^{1-2\delta } b_k^\dagger b_k \! + \! 1\right) . \end{aligned}$$

Choosing $\delta$ small enough consequently yields

$$\begin{aligned} \pm X_2\Big |_{{\mathcal {F}}^+_{rN}}\lesssim N^{\frac{11\kappa }{4}-\frac{1}{2}} \! \left( \widetilde{{\mathcal {N}}}+1\right) ^6 \! \Big ({\mathbb {K}}+1\Big )\Big |_{{\mathcal {F}}^+_{rN}}. \end{aligned}$$

We further have

$$\begin{aligned} \pm {\widetilde{X}}_1\Big |_{{\mathcal {F}}^+_{N-3}}\lesssim N^{\frac{11\kappa }{2}-1}\sqrt{\log N} \! \left( \widetilde{{\mathcal {N}}}+1\right) ^6 \! \Big ({\mathbb {K}}+1\Big )\Big |_{{\mathcal {F}}^+_{N-3}} \end{aligned}$$

by Corollary 2 and Corollary 3. Combining the results from Eq. (46), Eq. (47), Lemma 15 and Lemma 17, and using the estimates

$$\begin{aligned} \pm {\mathcal {E}}_2&\lesssim N^{4\kappa -1} \! \left( {\mathcal {N}}+1\right) ^2,\\ \pm \sum _{k\ne 0}|k|^2 |w_k|^2 N^{-2}\left( a_0^{2\dagger }a_0^2-N^2\right) a_k^\dagger a_k&\lesssim N^{5\kappa -1} \! \left( \widetilde{{\mathcal {N}}}+1\right) ^2, \end{aligned}$$

which are similar to the ones already obtained in Sect. 4.2, yields

$$\begin{aligned} \pm \! (1-G)^\dagger \left( {\mathcal {E}}_\textrm{odd}+{\mathcal {E}}_\textrm{log}-\sum _{i=1}^4 {\mathcal {E}}_i\right) (1-G)\bigg |_{{\mathcal {F}}^+_{rN}}&\lesssim N^{6\kappa -1}\! (1-G)^\dagger \left( \widetilde{{\mathcal {N}}}+1\right) ^3 (1-G) \Big |_{{\mathcal {F}}^+_{rN}}\\&\lesssim N^{6\kappa -1}\!\left( \widetilde{{\mathcal {N}}}+1\right) ^3 \Big |_{{\mathcal {F}}^+_{rN}}\\&\lesssim N^{\frac{11\kappa }{2}-1}\! \left( \widetilde{{\mathcal {N}}}+1\right) ^2 \! \Big ({\mathbb {K}}+1\Big )\Big |_{{\mathcal {F}}^+_{rN}} \! . \end{aligned}$$

Hence, $\pm X_1\Big |_{{\mathcal {F}}^+_{rN}}\lesssim N^{\frac{11\kappa }{2}-1}\sqrt{\log N} \! \left( \widetilde{{\mathcal {N}}}+1\right) ^6 \! \Big ({\mathbb {K}}+1\Big )\Big |_{{\mathcal {F}}^+_{rN}}$, which concludes together with the corresponding bound on $X_2$ the proof of

$$\begin{aligned} \pm X\Big |_{{\mathcal {F}}^+_{rN}}\le C_r N^{\frac{11\kappa }{4}-\frac{1}{2}} \! \left( \widetilde{{\mathcal {N}}}+1\right) ^6 \! \Big ({\mathbb {K}}+1\Big )\Big |_{{\mathcal {F}}^+_{rN}}. \end{aligned}$$

In the following let $\kappa :=0$. Using again the bound on $X_1$, we obtain

$$\begin{aligned} |{\langle {\chi _\delta \Gamma _1, X_1 \chi _\delta \Gamma _1}\rangle }|\lesssim \frac{\sqrt{\log N}}{N} \! \left\langle \chi _\delta \Gamma _1, \left( \widetilde{{\mathcal {N}}}+1\right) ^6 \! \Big ({\mathbb {K}}+1\Big ) \chi _\delta \Gamma _1\right\rangle \lesssim \frac{\sqrt{\log N}}{N}, \end{aligned}$$

where we have used that ${\mathfrak {b}}_k \Gamma _1=0$, and therefore $\left( \widetilde{{\mathcal {N}}}+1\right) ^6 \! \Big ({\mathbb {K}}+1\Big )\Gamma _1=\Gamma _1$, together with Lemma 11. Regarding $\left\langle \chi _\delta \Gamma _1,X_2 \, \chi _\delta \Gamma _1\right\rangle$, note that by Corollary 2 and Corollary 3

$$\begin{aligned} \pm \left( {\widetilde{X}}_2+{\widetilde{X}}_2^\dagger \right) \Big |_{{\mathcal {F}}^+_{N-3}}\lesssim \epsilon \left( \widetilde{{\mathcal {N}}}+1\right) ^4\left( {\mathbb {K}}+1\right) \Big |_{{\mathcal {F}}^+_{N-3}} +\epsilon ^{-1}{\mathbb {K}}\Big |_{{\mathcal {F}}^+_{N-3}}. \end{aligned}$$

Since ${\mathbb {K}}\Gamma _1=0$, we obtain $\left\langle \chi _\delta \Gamma _1,{\mathbb {K}} \, \chi _\delta \Gamma _1\right\rangle \lesssim N^{-2\lambda }$ for any $\lambda >0$ by Lemma 11, and consequently $\Big | \! \left\langle \chi _\delta \Gamma _1,{\widetilde{X}}_2 \, \chi _\delta \Gamma _1\right\rangle \! \Big |\lesssim N^{-\lambda }$. By a similar argument we obtain

$$\begin{aligned} \left\langle \chi _\delta \Gamma _1,W_\textrm{log} \, \chi _\delta \Gamma _1\right\rangle \lesssim N^{-\lambda }. \end{aligned}$$

In order to estimate the ${\mathcal {E}}_\textrm{odd}$ term, note that $\Gamma _1$ is a quasi-free state with respect to ${\mathfrak {b}}_k$, respectively ${\mathfrak {a}}_k$, and ${\mathcal {E}}_\textrm{odd}$ contains only odd powers in $\{a_k:k\in 2\pi {\mathbb {Z}}^3{\setminus } \{0\}\}$. Therefore, $e^{i\pi {\mathcal {N}}}\chi _\delta \Gamma _1=\chi _\delta e^{i\pi \sum _{k\ne 0}{\mathfrak {a}}_k^\dagger {\mathfrak {a}}_k}\Gamma _1=\chi _\delta \Gamma _1$ is invariant under $e^{i\pi {\mathcal {N}}}$, while $e^{-i\pi {\mathcal {N}}} {\mathcal {E}}_\textrm{odd} e^{i\pi {\mathcal {N}}}=-{\mathcal {E}}_\textrm{odd}$, and therefore $\left\langle \chi _\delta \Gamma _1, {\mathcal {E}}_\textrm{odd}\chi _\delta \Gamma _1\right\rangle =0$, and the same holds for $G^\dagger {\mathcal {E}}_\textrm{odd} G$. Hence

$$\begin{aligned} \left\langle \chi _\delta \Gamma _1, (1 - G)^\dagger {\mathcal {E}}_\textrm{odd}(1 - G)\chi _\delta \Gamma _1\right\rangle = -2\mathfrak {Re}\left\langle \chi _\delta \Gamma _1, {\mathcal {E}}_\textrm{odd}G\chi _\delta \Gamma _1\right\rangle . \end{aligned}$$

A rough, but easy, estimate shows that $G^\dagger G\lesssim N^{-1}\left( \widetilde{{\mathcal {N}}}+1\right) ^3$ and ${\mathcal {E}}_\textrm{odd}^2\lesssim N^{-1}\left( \widetilde{{\mathcal {N}}}+1\right) ^3$ in the case $\kappa =0$, and hence $\pm \left( {\mathcal {E}}_\textrm{odd}G+G^\dagger {\mathcal {E}}_\textrm{odd}\right) \lesssim N^{-1}\left( \widetilde{{\mathcal {N}}}+1\right) ^3$. Therefore, we obtain that

$$\begin{aligned} \Big | \! \left\langle \chi _\delta \Gamma _1, (1 - G)^\dagger {\mathcal {E}}_\textrm{odd}(1 - G)\chi _\delta \Gamma _1\right\rangle \! \Big |\lesssim \frac{1}{N}. \end{aligned}$$

In order to analyse the final term ${\mathcal {E}}_\textrm{log}$, note that we have by Lemma 6 together with Eq. (76)

$$\begin{aligned}&\pm \left\{ (1-G)^\dagger {\mathcal {E}}_{\textrm{log}}(1-G)-{\mathcal {E}}_\textrm{log}\right\} \\&\quad \lesssim \epsilon N^{3\delta -1} \left( \widetilde{{\mathcal {N}}} \! + \! 1\right) \! \! \left( \sum _k |k|^{1-2\delta } b_k^\dagger b_k \! + \! 1\right) +\frac{2}{\epsilon }N^{3\delta -1}G^\dagger \left( \widetilde{{\mathcal {N}}} \! + \! 1\right) \! \! \left( \sum _k |k|^{1-2\delta } b_k^\dagger b_k \! + \! 1\right) G\\&\quad \lesssim \left( \epsilon N^{3\delta -1}+\frac{2}{\epsilon }N^{3\delta -2}\right) \left( \widetilde{{\mathcal {N}}} \! + \! 1\right) \! \! \left( \sum _k |k|^{1-2\delta } b_k^\dagger b_k \! + \! 1\right) \\&\quad \lesssim \frac{1}{N}\left( \widetilde{{\mathcal {N}}} \! + \! 1\right) \left( {\mathbb {K}} \! + \! 1\right) . \end{aligned}$$

for $\epsilon >0$. Furthermore, note that we have ${\mathcal {E}}_\textrm{log}=\sum _{k \ell , k' \ell '}\Lambda _{k \ell , k' \ell '}\, {\mathfrak {a}}_{\ell '}^\dagger {\mathfrak {a}}_{k'}^\dagger {\widetilde{O}} {\mathfrak {a}}_k {\mathfrak {a}}_\ell$, where

$$\begin{aligned} {\widetilde{O}}:=\left( a_0^\dagger \frac{1}{\sqrt{a_0 a_0^\dagger }}\right) ^2\frac{a_0^\dagger a_0}{N}\left( \frac{1}{\sqrt{a_0 a_0^\dagger }}a_0\right) ^2 \end{aligned}$$

satisfies $\pm ({\widetilde{O}}-1)\lesssim \frac{{\mathcal {N}}}{N}$ and $\Lambda$ satisfies $\Vert \Lambda \Vert \lesssim 1$. Therefore,

$$\begin{aligned} \pm \left\{ {\mathcal {E}}_\textrm{log}-{\mathcal {E}}'_\textrm{log}\right\} \lesssim \frac{1}{N}{\mathcal {N}}^3\lesssim \frac{1}{N}(\widetilde{{\mathcal {N}}}+1)^3 \end{aligned}$$

with ${\mathcal {E}}'_{\textrm{log}}:=\sum _{k \ell , k' \ell '}\Lambda _{k \ell , k' \ell '}\, {\mathfrak {a}}_{\ell '}^\dagger {\mathfrak {a}}_{k'}^\dagger {\mathfrak {a}}_k {\mathfrak {a}}_\ell$, and hence

$$\begin{aligned} \pm \Big \langle \chi _\delta \Gamma _1, \Big \{(1-G)^\dagger {\mathcal {E}}_{\textrm{log}}(1-G)-{\mathcal {E}}'_\textrm{log}\Big \} \chi _\delta \Gamma _1\Big \rangle \lesssim \frac{1}{N}. \end{aligned}$$

Using again that $\Vert \Lambda \Vert \lesssim 1$ and Lemma 11 further yields

$$\begin{aligned} \left| \! \Big \langle \chi _\delta \Gamma _1, {\mathcal {E}}'_{\textrm{log}} \chi _\delta \Gamma _1 \! \Big \rangle \! {-}\! \Big \langle \Gamma _1, {\mathcal {E}}'_{\textrm{log}} \Gamma _1 \! \Big \rangle \! \right| \!&\lesssim \! \frac{1}{N} {\langle {\Gamma _1, \! ({\mathcal {N}}\! {+}\! 1)^2 \Gamma _1}\rangle }\! +\! N \! {\langle {(1\! -\! \chi _\delta )\Gamma _1, \! ({\mathcal {N}}\! +\! 1)^2 (1\! -\! \chi _\delta )\Gamma _1}\rangle }\!\\  &\lesssim \! \frac{1}{N}. \end{aligned}$$

Finally, we note that ${\mathfrak {a}}_k {\mathfrak {a}}_\ell \Gamma _1=-\gamma _k\nu _k \delta _{\ell +k=0} \Gamma _1+\nu _k \nu _\ell {\mathfrak {b}}_k {\mathfrak {b}}_\ell \Gamma _1$, which allows us to compute

$$\begin{aligned} \Big \langle \Gamma _1, {\mathcal {E}}'_{\textrm{log}} \Gamma _1 \! \Big \rangle =D_\textrm{log}+\sum _{k \ell }\Lambda _{k \ell , k \ell }\nu _k^2 \nu _\ell ^2. \end{aligned}$$

Combining what we have so far, and using that

$$\begin{aligned} |\sum _{k \ell }\Lambda _{k \ell , k \ell }\nu _k^2 \nu _\ell ^2|&\lesssim \frac{1}{N},\\ \left| D_\textrm{log}-D_\textrm{log}\Vert \chi _\delta \Gamma _1\Vert ^2\right|&\lesssim N^{-\lambda } \end{aligned}$$

for $\lambda >0$, we obtain

$$\begin{aligned} \left| \Big \langle \chi _\delta \Gamma _1, \{(1-G)^\dagger {\mathcal {E}}_{\text {log}}(1-G)-D_\text {log}\} \chi _\delta \Gamma _1 \! \Big \rangle \right| \lesssim \frac{1}{N}. \end{aligned}$$

This concludes the proof of $\big | \! \left\langle \chi _\delta \Gamma _1,X \, \chi _\delta \Gamma _1\right\rangle \! \big | \lesssim \frac{\sqrt{log N}}{N}$. $\square$

Using 4, we are in a position to verify Theorem 3 by writing ${\langle {\Psi _d, H_{N,\kappa }' \Psi _d}\rangle }$ as

77 $$\begin{aligned}&Z_d^{-2} \left\langle \chi _\delta \Gamma _d, \left( C_\text {log}+ {\mathbb {K}} + \! \left( W_\text {log}+W_\text {log}^\dagger \right) \! + X_1 +X_2\right) \chi _\delta \Gamma _d\right\rangle \nonumber \\  &\quad =\lambda _{N,\kappa }^{(d)} \! + \! \left( Z_d^{-2} \left\langle \chi _\delta \Gamma _d, {\mathbb {K}} \chi _\delta \Gamma _d\right\rangle \! - \! \lambda _{N,\kappa }^{(d)}\right) \! \nonumber \\  &\quad + Z_d^{-2} \left\langle \chi _\delta \Gamma _d,\right. \left. \left( C_\text {log} \! + \! W_\text {log} \! + \! W_\text {log}^\dagger \! + \! X_1 \! + \! X_2\right) \chi _\delta \Gamma _d\right\rangle . \end{aligned}$$

In the following we are going to decompose $Z_d^{-2} \left\langle \chi _\delta \Gamma _d, {\mathbb {K}} \chi _\delta \Gamma _d\right\rangle \! - \! \lambda _{N,\kappa }^{(d)}$ as

$$\begin{aligned}&Z_d^{-2}\! \Big (\! \! \left\langle \chi _\delta \Gamma _d, {\mathbb {K}} \chi _\delta \Gamma _d\right\rangle \! -\! \left\langle \Gamma _d, {\mathbb {K}} \Gamma _d\right\rangle \! \! \Big )\! +\! Z_d^{-2}\! \! \left( \left\langle \Gamma _d , {\mathbb {K}}\Gamma _d\right\rangle \! - \! {\widetilde{\lambda }}_{N,\kappa }^{(d)}\right) \! +\! Z_d^{-2}\! \! \left( {\widetilde{\lambda }}_{N,\kappa }^{(d)}\! -\! \lambda ^{(d)}_{N,\kappa }\! \right) \! \\  &\quad +\! \lambda ^{(d)}_{N,\kappa }\! \left( 1\! -\! Z_d^{-2}\right) . \end{aligned}$$

The first term $\left\langle \chi _\delta \Gamma _d, {\mathbb {K}} \chi _\delta \Gamma _d\right\rangle \! -\! \left\langle \Gamma _d, {\mathbb {K}} \Gamma _d\right\rangle$ decays faster than any power in $N^{-\lambda }$ by Lemma 11. Regarding $\left\langle \Gamma _d, {\mathbb {K}}\Gamma _d\right\rangle \! - \! {\widetilde{\lambda }}_{N,\kappa }^{(d)}$, note that $\Gamma _d$ is an eigenvector of $\sum _{k\ne 0}e_k {\mathfrak {b}}_k^\dagger {\mathfrak {b}}_k$ to the eigenvalue ${\widetilde{\lambda }}^{(d)}_{N,\kappa }$, and as such has a finite number of excitations, i.e. there exists a finite set $I_d\subset 2\pi {\mathbb {Z}}^3{\setminus } \{0\}$ and a $C_d$, such that ${\mathfrak {b}}_k \Gamma _d=0$ for $k\notin I$ and $\Vert {\mathfrak {b}}_j {\mathfrak {b}}_k \Gamma _d\Vert \le C$, and therefore we obtain

78 $$\begin{aligned} \pm \left( \left\langle \Gamma _d , {\mathbb {K}}\Gamma _d\right\rangle - {\widetilde{\lambda }}_{N,\kappa }^{(d)}\right)&= \pm \frac{1}{2}\sum _{jk, mn}\left( V_{N^{1-\kappa }}\right) _{jk , mn}{\langle {{\mathfrak {b}}_j {\mathfrak {b}}_k \Gamma _d,{\mathfrak {b}}_m {\mathfrak {b}}_n \Gamma _d}\rangle }\nonumber \\  &\le C_d^2 |I_d|^4 \Vert {\widehat{V}}\Vert _\infty N^{\kappa -1}, \end{aligned}$$

Regarding the third term we have $\left| {\widetilde{\lambda }}_{N,\kappa }^{(d)}\! -\! \lambda ^{(d)}_{N,\kappa }\right| \lesssim N^{2\kappa -1}$, see the estimate above Eq. (61), and again by Lemma 11, we have $\left| 1\! -\! Z_d^{-2}\right| \lesssim N^{-\lambda }$ for any $\lambda >0$. Therefore

$$\begin{aligned} \left| Z_d^{-2} \left\langle \chi _\delta \Gamma _d, {\mathbb {K}} \chi _\delta \Gamma _d\right\rangle \! - \! \lambda _{N,\kappa }^{(d)}\right| \lesssim N^{2\kappa -1}. \end{aligned}$$

Note that for any $\lambda \ge 1-\frac{13\kappa }{4}$ by Theorem 4 and Lemma 11

$$\begin{aligned} Z_d^{-2} \left\langle \chi _\delta \Gamma _d, \left( \! X_1 \! + \! X_2\right) \chi _\delta \Gamma _d\right\rangle \lesssim N^{\frac{11\kappa }{4}-\frac{1}{2}}{\langle {\Gamma _d,\left( \widetilde{{\mathcal {N}}}+1\right) ^6 \! \Big ({\mathbb {K}}+1\Big )\Gamma _d}\rangle }+C_\lambda N^{-\lambda }\lesssim N^{\frac{13\kappa }{4}-\frac{1}{2}} \end{aligned}$$

Arguing similar as in Eq. (78) we further obtain $\big | \! {\langle {\Gamma _d, W_\textrm{log} \Gamma _d}\rangle }\! \big |\lesssim N^{2\kappa -\frac{1}{2}}$, and therefore

$$\begin{aligned} \big | \! Z_d^{-2}{\langle {\chi _\delta \Gamma _d, W_\textrm{log} \chi _\delta \Gamma _d}\rangle }\! \big |\lesssim N^{2\kappa -\frac{1}{2}} \end{aligned}$$

by Lemma 11. Regarding the constant $C_\textrm{log}=\sum _{k} \Pi _k-D_\textrm{log}$, note that we have the inequality $|\sum _k \Pi _k|\lesssim N^{\frac{9\kappa }{2}}\frac{\log N}{N}$ by Corollary 2 and $|D_\textrm{log}|\lesssim N^{\frac{9\kappa }{2}}\frac{\log N}{N}$ by Theorem 4, and therefore

79 $$\begin{aligned} {\langle {\Psi _d, H_{N,\kappa }' \Psi _d}\rangle }\le {\widetilde{\lambda }}_{N,\kappa }^{(d)}+ C N^{\frac{13\kappa }{4}-\frac{1}{2}}. \end{aligned}$$

In case of $\kappa =0$ and $d=1$ we can further improve Eq. (79). Starting again with Eq. (77), we make use of the fact that ${\langle {\Gamma _1,{\mathbb {K}}\Gamma _1}\rangle }=0$ and use the estimates $\big | \! \left\langle \chi _\delta \Gamma _1,X \, \chi _\delta \Gamma _1\right\rangle \! \big | \lesssim \frac{\sqrt{\log N}}{N}$ and $\big | \! \left\langle \chi _\delta \Gamma _1,W_\textrm{log} \, \chi _\delta \Gamma _1\right\rangle \! \big | \lesssim \frac{1}{N}$ from Theorem 4, in order to obtain for any $\lambda >0$ and suitable constants $C',C>0$ by Lemma 11

80 $$\begin{aligned} {\langle {\Psi _1, H_{N,0}' \Psi _1}\rangle }\le Z_d^{-2} C_\textrm{log}+{\langle {\Gamma _1,{\mathbb {K}}\Gamma _1}\rangle }+C'\frac{\sqrt{\log N}}{N}+C' N^{-\lambda }\le C\frac{\log N}{N}. \end{aligned}$$

Using the definition of the excitation Hamiltonian

$$\begin{aligned} H'_{N,\kappa }=H_{N,\kappa }-4\pi {\mathfrak {a}}_{N^{1-\kappa }}N^\kappa (N-1)- \frac{1}{2}\sum _{k\ne 0}\left\{ \sqrt{A_k^2-B_k^2}-A_k+C_k\right\} , \end{aligned}$$

Eq. (79) and Eq. (80) immediately yield

$$\begin{aligned} {\langle {\Psi _d,H_{N,\kappa }\Psi _d}\rangle }&\le 4\pi {\mathfrak {a}}_{N^{1-\kappa }}N^\kappa (N-1)+ \frac{1}{2}\sum _{k\ne 0}\left\{ \sqrt{A_k^2-B_k^2}-A_k+C_k\right\} \\  &\quad +{\widetilde{\lambda }}_{N,\kappa }^{(d)}+C N^{\frac{13\kappa }{4}-\frac{1}{2}},\\ {\langle {\Psi _1,H_{N,0}\Psi _1}\rangle }&\le 4\pi {\mathfrak {a}}_{N^{1-\kappa }}N^\kappa (N-1)+ \frac{1}{2}\sum _{k\ne 0}\left\{ \sqrt{A_k^2-B_k^2}-A_k+C_k\right\} +C\frac{\log N}{N}, \end{aligned}$$

which concludes the proof of Theorem 3 since we have by Eq. (59) that

$$\begin{aligned} \left| \sum _{k\ne 0}\left\{ \sqrt{A_k^2-B_k^2}-A_k+C_k\right\} -\sum _{k\ne 0}\left\{ \sqrt{|k|^4+16\pi {\mathfrak {a}} N^\kappa |k|^2}+|k|^2+8\pi {\mathfrak {a}} N^\kappa -\frac{(8\pi {\mathfrak {a}} N^\kappa )^2}{2|k|^2}\right\} \right| \end{aligned}$$

is of order $N^{4\kappa -1}\log N$ in the case $K:=\infty$.

## Coefficients of the Renormalized Potential

In this part of the Appendix, we are going to verify essential properties of the box scattering length ${\mathfrak {a}}_L$ and the renormalized potential $V_{L}-V_{L} R V_{L}$, starting with the proof of $|{\mathfrak {a}}_L-{\mathfrak {a}}|\lesssim L^{-1}$ in the following Lemma 12, which we will subsequently use in order to verify Lemma 1.

### Lemma 12

Let $V\in L^1 \! \left( {\mathbb {R}}^3\right)$. Then there exists a constant $C>0$, such that

$$\begin{aligned} |{\mathfrak {a}}_L-{\mathfrak {a}}|\le \frac{C}{L}. \end{aligned}$$

Furthermore, for any $v\ge 0$ the constant $C>0$ depends uniformly on *V* for $\Vert V\Vert _{L^1\! \left( {\mathbb {R}}^3\right) }\le v$.

### Proof

Let $\omega$ be the radial solution of $\left( -2\Delta _x+V\right) \omega =V$ in ${\mathbb {R}}^3$ satisfying $\omega (x) \underset{|x|\rightarrow \infty }{\longrightarrow }0$, which allows us to express the scattering length ${\mathfrak {a}}$ as

$$\begin{aligned} 8\pi {\mathfrak {a}}:=\int _{{\mathbb {R}}^3}V(x)\, \textrm{d}x-{\langle {V,\omega }\rangle }. \end{aligned}$$

Note that ${\langle {V,\omega }\rangle }$ is well-defined, since $\omega$ has values in [0, 1] and *V* is in $L^1 \! \left( {\mathbb {R}}^3\right)$. Furthermore, we introduce the re-scaled objects

$$\begin{aligned} V_L(x)&:= L^2 V(L x) ,\\ \omega _L(x)&:= \omega (L x) . \end{aligned}$$

In order to verify the Lemma, let us use the solution $\omega$ in order to create an approximation of the box scattering solution $R V_L\in L^2\! \left( \Lambda ^2\right)$, i.e. for a smooth cut-off function $0\le \chi \le 1$ satisfying $\textrm{supp}(\chi )\subset \textrm{int}(\Lambda )$ and $\chi (x)=1$ for all $|x|\le \frac{1}{4}$, let us define

$$\begin{aligned} \psi _L(x,y)&:= \chi (x-y)\omega _L(x-y),\\ \xi _L(x,y)&:= 2(\Delta \chi )(x-y)\omega _L(x-y)+4(\nabla \chi )(x-y)\nabla \omega _L(x-y), \end{aligned}$$

where $x-y$ is the distance on the torus. For *L* large enough such that $\chi V_L=V_L$, the function $\psi _L\in L^2\! \left( \Lambda ^2\right)$ then satisfies the differential equation

$$\begin{aligned} \big (\! -\! \Delta _2\! +\! V_L\big )\psi _L&=V_L-\xi _L, \end{aligned}$$

where $\Delta _2:=\Delta _x + \Delta _y$, see Sect. 2. Using $R \big (\! -\! \Delta _2\! +\! V_L \big )=1-(1-R V_L)(1-\pi _{{\mathcal {H}}})$, and $(1-\pi _{{\mathcal {H}}})\psi _L=\left( \int \chi \omega _L\right) \! 1$, we can therefore express $R V_L$ as

81 $$\begin{aligned} R V_L=\psi _L+R \xi _L-\left( \int _{{\mathbb {R}}^3} \chi \omega _L\right) \! \big (1-R V_L\big ). \end{aligned}$$

Since *V* has compact support, we have $L{\langle {V_L,\psi _L}\rangle }=L{\langle {V_L,\omega _L}\rangle }={\langle {V,\omega }\rangle }$ for *L* large enough. Consequently, we can express $8\pi {\mathfrak {a}}_L$ according to Eq. (81) as

$$\begin{aligned} 8\pi {\mathfrak {a}}_L&\! = \! L {\langle {V_L,(1 \! - \! R V_L)}\rangle } \\&= \! \int _{{\mathbb {R}}^3}V \! - \! L{\langle {V_L,RV_L}\rangle } \\&= \! \int _{{\mathbb {R}}^3}V \! - \! {\langle {V,\omega }\rangle } \! - \! L \! {\langle {V_L,R \xi _L}\rangle } \! + \! 8\pi {\mathfrak {a}}_L \! \int _{{\mathbb {R}}^3} \! \chi \omega _L\\&= 8 \pi {\mathfrak {a}}-L \! {\langle {V_L,R \xi _L}\rangle } +8\pi {\mathfrak {a}}_L \! \int _{{\mathbb {R}}^3} \chi \omega _L, \end{aligned}$$

or equivalently

82 $$\begin{aligned} 8\pi {\mathfrak {a}}-8\pi {\mathfrak {a}}_L=\left( 1-\frac{1}{1-\int _{{\mathbb {R}}^3} \chi \omega _L}\right) 8\pi {\mathfrak {a}}+\frac{1}{1-\int _{{\mathbb {R}}^3} \chi \omega _L}L \! {\langle {R V_L, \xi _L}\rangle }. \end{aligned}$$

Note that $0\le \int _\Lambda \chi \omega _L \le \frac{C}{L}$, which can be verified easily utilizing the bounds $\Vert {\widehat{\chi }}\Vert _\infty <\infty$ and

$$\begin{aligned} 2L |k|^2 |\widehat{\omega _L}(k)|=2 \left| \frac{k}{L}\right| ^2 \left| {\widehat{\omega }}\left( \frac{k}{L}\right) \right| =\left| {\widehat{V}} \! \left( \frac{k}{L}\right) -\left\langle e^{i\frac{k}{L}x}V,\frac{1}{-2\Delta +V}V\right\rangle \right| \lesssim 1, \end{aligned}$$

where we have used $V\frac{1}{-2\Delta +V}V\le V$ and $|{\langle {e^{i\frac{k}{L}x},Ve^{i\frac{k}{L}x}}\rangle }|= \int _{{\mathbb {R}}^3}V<\infty$. Consequently, the first term on the right hand side of Eq. (82) is of the order $\frac{1}{L}$. Regarding the second term, note that the support of $\Delta \chi (x-y)$ and $\nabla \chi (x-y)$ is contained in $\textrm{int}(\Lambda )\setminus B_{\frac{1}{4}}(0)$, which is disjoint to the support of $V_L$ for *L* large enough, and therefore $\omega _L(x-y)=\frac{{\mathfrak {a}}}{L|x-y|}$ on the support of $\Delta \chi (x-y)$, respectively on the support of $\nabla \chi (x-y)$. This especially means that $\xi _L=\frac{1}{L}\xi$, where we define the $C^\infty _0$ function

$$\begin{aligned} \xi (x,y):=2(\Delta \chi )(x-y)\frac{{\mathfrak {a}}}{|x-y|}-4(\nabla \chi )(x-y)\frac{(x-y){\mathfrak {a}}}{|x-y|^3}. \end{aligned}$$

Hence,

$$\begin{aligned} \left| L{\langle {R V_L,\xi _L}\rangle }\right| \! = \! \left| \left\langle (-\Delta _2)^{-1}R V_L,(-\Delta _2)\xi \right\rangle \right| \! \lesssim \! \Vert (-\Delta _2)^{-1}R V_L\Vert . \end{aligned}$$

In order to compute $\Vert (-\Delta _2)^{-1}R V_L\Vert$, note that the coefficients

$$\begin{aligned} f_k:={\langle {e^{ik(x-y)},(-\Delta _2)R V_L}\rangle }=\left( V_L-V_L R V_L\right) _{k(-k), 0 0} \end{aligned}$$

satisfy $|f_k|\le \frac{D}{L}$ for a suitable constant *D*. Therefore, we obtain

$$\begin{aligned} \Vert (-\Delta _2)^{-1}R V_L\Vert ^2=\sum _k \frac{|f_k|^2}{|k|^4}\le \frac{D}{L^2}\sum _k \frac{1}{|k|^4}, \end{aligned}$$

and consequently $\Vert (-\Delta _2)^{-1}R V_L\Vert \le \frac{C}{L}$ for a suitable constant *C*. $\square$

With the result from Lemma 12 we are finally in a position to verify Lemma 1.

### Proof

(Proof of Lemma 1) Let us first derive the bound on the coefficients $L\Big (V_{L}-V_{L} R V_{L}\Big )_{k_1 k_2, k_3 k_4}$. Since $V\in L^1 \! \left( {\mathbb {R}}^3\right)$ and $VRV\ge 0$, it is enough to bound $L\Big (V_{L} R V_{L}\Big )_{k_1 k_2, k_1 k_2}$. For $(k_1,k_2)\in {\mathcal {H}}$

$$\begin{aligned}&L\Big (V_{L} R V_{L}\Big )_{k_1 k_2, k_1 k_2} \! \! = \! L{\langle {e^{ik_1 x}e^{i k_2 y},\pi _{{\mathcal {H}}}V_L RV_L \pi _{{\mathcal {H}}}e^{ik_1 x}e^{i k_2 y}}\rangle } \! \\&\quad \le \! L{\langle {e^{ik_1 x}e^{i k_2 y},V_L e^{ik_1 x}e^{i k_2 y}}\rangle } \! = \! \int _{{\mathbb {R}}^3} \! V, \end{aligned}$$

where we have used $\pi _{{\mathcal {H}}}V_L RV_L \pi _{{\mathcal {H}}}\le \pi _{{\mathcal {H}}}V_L \pi _{{\mathcal {H}}}$. Regarding the momentum pairs $(k_1,k_2)\in {\mathcal {L}}$, let us define the operator *X* and the function $\chi :{\mathbb {R}}^6\longrightarrow [0,1]$ as

$$\begin{aligned} X&:= \pi _{{\mathcal {L}}}V_L R V_L \pi _{{\mathcal {L}}} ,\\ \chi (x,y)&:= \mathbbm {1}_{B_{L^{-1}R}(0)}(x-y), \end{aligned}$$

where *R* is such that $\textrm{supp}(V)\subset B_R(0)$. Then we obtain by Cauchy-Schwarz

83 $$\begin{aligned} V_L R V_L&=\chi V_L R V_L \chi \nonumber \\&\lesssim \chi \pi _{{\mathcal {H}}} V_L R V_L \pi _{{\mathcal {H}}} \chi +\chi \pi _{{\mathcal {L}}} V_L R V_L\pi _{{\mathcal {L}}}\chi \nonumber \\&\le \chi \pi _{{\mathcal {H}}} V_L \pi _{{\mathcal {H}}} \chi +\chi \pi _{{\mathcal {L}}} X \pi _{{\mathcal {L}}}\chi \nonumber \\&\lesssim V_L +\chi \pi _{{\mathcal {L}}} (V_L+X) \pi _{{\mathcal {L}}}\chi . \end{aligned}$$

Using $\pi _{{\mathcal {L}}} \chi \pi _{{\mathcal {L}}}\le 2\int _{\Lambda ^2} \chi \lesssim L^{-3}$ and $\pi _{{\mathcal {L}}} V_L \pi _{{\mathcal {L}}}\le 2\int _{\Lambda ^2} V_L\lesssim L^{-1}$ we especially obtain

$$\begin{aligned} \Vert X\Vert \le \Vert \pi _{{\mathcal {L}}} V_L \pi _{{\mathcal {L}}}\Vert +\Vert \pi _{{\mathcal {L}}} \chi \pi _{{\mathcal {L}}}\Vert ^2 \left( \Vert \pi _{{\mathcal {L}}} V_L \pi _{{\mathcal {L}}}\Vert +\Vert X\Vert \right) \lesssim L^{-1}+L^{-6}\Vert X\Vert , \end{aligned}$$

and therefore $\Vert X\Vert \lesssim L^{-1}$, which implies $L\Big (V_{L} R V_{L}\Big )_{k_1 k_2, k_1 k_2}\lesssim 1$ for $(k_1,k_2)\in {\mathcal {L}}$.

In order to verify Eq. (11), let $W(x,y):=e^{i k_1 x}e^{i k_2 y}-e^{i(k_1+k_2)x}$. By Eq. (83) we have

$$\begin{aligned} L {\langle {V_L W, R V_L W}\rangle }\lesssim L {\langle {W, V_L W}\rangle }+\Vert \pi _{{\mathcal {L}}}\chi W\Vert ^2\lesssim L {\langle {W, V_L W}\rangle }+L^{-6}, \end{aligned}$$

where we have used that $\Vert \pi _{{\mathcal {L}}} V_L \pi _{{\mathcal {L}}}\Vert , \Vert X\Vert \lesssim L^{-1}$ and $\Vert \pi _{{\mathcal {L}}}\chi W\Vert =\left| \int _{\Lambda ^2}\chi W\right| \lesssim L^{-3}$. Further

$$\begin{aligned} L {\langle {W, V_L W}\rangle }=\int _{{\mathbb {R}}^3} V( z)|e^{i k L^{-1} z}-1|^2\, \textrm{d}z\le L^{-2}|k|^2\int _{{\mathbb {R}}^3}V(z)|z|^2\, \textrm{d}z\lesssim L^{-2}|k_2|^2, \end{aligned}$$

where we have used that *V* has compact support and $V\in L^1 \! \left( {\mathbb {R}}^3\right)$. Consequently

84 $$\begin{aligned} \left| \! L\Big (V_{L} R V_{L}\Big )_{k_1 k_2,k_3 k_4} \! - \! L\Big (V_{L} R V_{L}\Big )_{(k_1+k_2) 0 ,k_3 k_4} \! \right| \!&= \! L \left| \left\langle V_L W, R V_L e^{ik_3 x}e^{ik_4 y}\right\rangle \right| \nonumber \\  &\le \sqrt{L {\langle {V_L, R V_L}\rangle }}\sqrt{L {\langle {V_L W, R V_L W}\rangle }} \nonumber \\  &\lesssim \! L^{-1} |k_2|. \end{aligned}$$

By Eq. (84) and the fact that $\left| L\Big (V_{L}-V_{L} R V_{L}\Big )_{00,00}-8\pi {\mathfrak {a}} \right| \lesssim L^{-1}$, see Lemma 12, we observe that it is enough to verify

85 $$\begin{aligned} \left| \! L\Big (V_{L} R V_{L}\Big )_{k 0,k 0} \! - \! L\Big (V_{L} R V_{L}\Big )_{0 0 ,0 0} \! \right| \lesssim L^{-1}|k| \end{aligned}$$

in order to prove Eq. (11). In order to prove Eq. (85), let us introduce the projection $\pi$ onto the momentum pairs $(\ell _1,\ell _2)$ with $\ell _2\ne 0$ as well as the boosted Laplace operator

$$\begin{aligned} -\Delta ^{(p)}_{2}:=(\frac{1}{i}\nabla _x+p)^2+(\frac{1}{i}\nabla _y)^2, \end{aligned}$$

which satisfies $-\Delta ^{(p)}_{2}=e^{-ip x}(-\Delta _2)e^{ip x}$ for $p\in 2\pi {\mathbb {Z}}^3$. Furthermore, let us introduce the pseudo-inverse $R_p$ of the operator $\pi \left( -\Delta ^{(p)}_{2}+V_L\right) \pi$, and define the state

$$\begin{aligned} \psi :=e^{-ipx}R e^{ipx}V_L. \end{aligned}$$

Using $(-\Delta _2 \! + \! V_L)R=1-(1-\pi _{\mathcal {H}})(1-V_L R)$, yields the equation

$$\begin{aligned} \pi (-\Delta ^{(p)}_{2} \! + \! V_L)\psi&= \pi e^{-ipx}(-\Delta _2 \! + \! V_L)R e^{ipx}V_L= \pi V_L - \lambda e^{ip(y-x)} \end{aligned}$$

where

$$\begin{aligned} \lambda :=\mathbbm {1}(|p|<K)(V_L-V_L R V_L)_{0 p ,p 0}. \end{aligned}$$

Using $R_p (-\Delta ^{(p)}_{2} \! + \! V_L)=1-(1-R_p V_L)(1-\pi )$ we therefore obtain $\psi =R_p V_L - \lambda R_p e^{ip(y-x)}$. By the first part of this proof we have $|\lambda |\lesssim L^{-1}$ and similarly $|{\langle {V_L,R_p e^{i\ell _1 x} e^{i \ell _2 y}}\rangle }|\lesssim L^{-1}$ for $\ell _1,\ell _2\in 2\pi {\mathbb {Z}}^3$, and consequently

$$\begin{aligned} \left| L\Big (V_{L} R V_{L}\Big )_{p 0, p 0}-L{\langle {V_L, R_p V_L}\rangle }\right| =L \left| \lambda {\langle {V_L,R_p e^{ip(y-x)}}\rangle }\right| \lesssim L^{-1}. \end{aligned}$$

In order to prove Eq. (85), it is consequently enough to verify

86 $$\begin{aligned} \left| {\langle { V_L, R_p V_L}\rangle }-{\langle { V_L, R_0 V_L}\rangle }\right| \lesssim L^{-2}|p|. \end{aligned}$$

In order to show Eq. (86), note that $R_p-R_0=R_0\left( -|p|^2+2i\nabla _x\cdot p\right) R_p$ and therefore

87 $$\begin{aligned} \left\langle V_L,R_p V_L \right\rangle \! - \! \left\langle V_L,R_0 V_L \right\rangle&= \frac{1}{2} \! \left\langle V_L, \left( R_p - R_0 \right) V_L \right\rangle + \frac{1}{2} \left\langle V_L, \left( \! R_{-p} - R_0 \! \right) V_L \right\rangle \nonumber \\&= -\frac{|p|^2}{2}\left\langle V_L,R_0 \left( R_p+R_{-p}\right) V_L \right\rangle \nonumber \\&\quad + \left\langle V_L,R_0 \left( i\nabla _x\cdot p\right) \left( R_p-R_{-p}\right) V_L \right\rangle \nonumber \\&= - \! \frac{|p|^2}{2}\sum _{q\ne 0} \overline{\psi _0(q)}\big (\psi _p(q) \! + \! \psi _{-p}(q)\big ) \! \nonumber \\&\quad + \! \sum _{q\ne 0} (p\cdot q)\overline{\psi _0(q)}\big (\psi _p(q) \! - \! \psi _{-p}(q)\big ) \end{aligned}$$

with $\psi _p:=R_{p} V_L$. Defining $\eta _p:=-\Delta _2^{(p)}\psi _p$, we note that

$$\begin{aligned} \eta _p(q)=L^{-1}{\widehat{V}}\left( \frac{q}{L}\right) -{\langle {e^{iq(x-y)}V_L,R_p V_L}\rangle } \end{aligned}$$

for $q\ne 0$ and therefore $|\eta _p(q)|\lesssim \frac{1}{L}$, which can be verified similarly to the bounds on $(V_L R V_L)_{k_1 k_2,k_3 k_4}$ from the first part of this proof. Consequently, $|\psi _p(q)|\lesssim \frac{1}{L(|q|^2+|q+p|^2)}$ and

88 $$\begin{aligned} \frac{|p|^2}{2}\left| \sum _{q\ne 0} \overline{\psi _0(q)}\big (\psi _p(q)+\psi _{-p}(q)\big )\right| \lesssim L^{-2}|p|^2 \sum _{q\ne 0}\frac{1}{2|q|^2 (|q|^2+|q+p|^2)}\lesssim L^{-2}|p|. \end{aligned}$$

Regarding the second term in Eq. (87), let us identify $\sum _{q\ne 0} (p\cdot q)\overline{\psi _0(q)}\big (\psi _p(q)-\psi _{-p}(q)\big )$ as

89 $$\begin{aligned}&\sum _{q\ne 0} \frac{(p\cdot q)\overline{\eta _0(q)}\eta _p(q)}{2|q|^2}\left( \frac{1}{|q|^2 \! + \! |q \! + \! p|^2} \! - \! \frac{1}{|q|^2 \! + \! |q \! - \! p|^2}\right) \! \nonumber \\&\quad + \! \sum _{q\ne 0} \frac{(p\cdot q)\overline{\psi _0(q)}\left( \eta _p(q) \! - \! \eta _{-p}(q)\right) }{|q|^2 \! + \! |q \! - \! p|^2}. \end{aligned}$$

Starting with the first term in Eq. (89), let $f(q,p):=\frac{1}{2|q|^2}\left( \frac{1}{|q|^2 + |q + p|^2} - \frac{1}{|q|^2 + |q - p|^2}\right)$ and note

$$\begin{aligned}&\left| \sum _{q\ne 0}(p\cdot q)\overline{\eta _0(q)}\eta _p(q) f(q,p)\right| \lesssim L^{-2}|p|\sum _{q\ne 0}|q| |f(q,p)|\\&\quad \lesssim L^{-2}|p|\int _{{\mathbb {R}}^3}|q| |f(q,p)| \, \textrm{d}q=L^{-2}|p|\mu , \end{aligned}$$

with $\mu :=\int _{{\mathbb {R}}^3}|q| |f(q,e)| \, \textrm{d}q<\infty$ and *e* being a unit vector. Regarding the second term in Eq. (89), we define $\psi _{p,q}:=R_{p} e^{iq(x-y)}V_L$, which again satisfies $|\psi _{p,q}(\ell )|\lesssim \frac{1}{L(|\ell |^2+|\ell +p|^2)}$. Using Lemma 14 and the fact that $R_{-p}-R_p=-4 R_{-p} \left( i\nabla _x\cdot p\right) R_{p}$, we observe that

$$\begin{aligned} \left| \eta _p(q) - \eta _{-p}(q)\right|&=\left| \left\langle e^{iq(x-y)}V_L,\left( R_{-p}-R_p\right) V_L\right\rangle \right| \\&=4\left| \sum _{\ell \ne 0}(p\cdot \ell )\overline{\psi _{-p,q}(\ell )}\psi _p(\ell )\right| \\&\le 4|p| \sqrt{\sum _{\ell \ne 0}|\psi _{-p,q}(\ell )|^2}\sqrt{\sum _{\ell \ne 0}|\ell |^2 |\psi _{p}(\ell )|^2}\\&\lesssim |p| \sqrt{L^{-2}|p|^{-1}}\sqrt{L^{-1}}. \end{aligned}$$

Consequently, we can estimate the second term in Eq. (89), using Lemma 14 again, by

$$\begin{aligned} \left| \sum _{q\ne 0} \frac{(p\cdot q)\overline{\psi _0(q)}\left( \eta _p(q) \! - \! \eta _{-p}(q)\right) }{|q|^2 \! + \! |q \! - \! p|^2}\right|&\lesssim L^{-\frac{3}{2}}|p|^{\frac{3}{2}}\sum _{q\ne 0}\frac{|q||\psi _0(q)|}{|q|^2 \! + \! |q \! - \! p|^2}\\&\le L^{-\frac{3}{2}}|p|^{\frac{3}{2}}\\  &\quad \times \sqrt{\sum _{q\ne 0}\left( |q|^2 \! + \! |q \! - \! p|^2\right) ^{-2}}\sqrt{\sum _{q\ne 0}|q|^2 |\psi _0(q)|^2}\\&\lesssim L^{-\frac{3}{2}}|p|^{\frac{3}{2}}\sqrt{|p|^{-1}}\sqrt{L^{-1}}. \end{aligned}$$

$$\square $$

With Lemma 1 at hand, we can furthermore verify the following Lemma 13.

### Lemma 13

Let $A_k$, $B_k$ and $C_k$ be the coefficients defined above Eq. (34), and let us define $f_k:=\sqrt{A_k^2-B_k^2}-A_k+C_k$ and $g_k:=\sqrt{|k|^4+16\pi {\mathfrak {a}} N^\kappa |k|^2}-|k|^2-8\pi {\mathfrak {a}} N^\kappa +\frac{(8\pi {\mathfrak {a}} N^\kappa )^2}{2|k|^2}$. Then,

$$\begin{aligned} \sum _{k\ne 0} \Big |f_k-g_k\Big | \le C\frac{N^{4\kappa }\log N}{N}+\frac{N^{3\kappa }}{K} \end{aligned}$$

for $K\ge K_0 N^{\frac{\kappa }{2}}$ and suitable $K_0,C$, where *C* depends uniformly on *V* in the sense of Eq. (12).

### Proof

We will verify the statement separately for the different terms $\sum _{0<|k|\le CN^{\frac{\kappa }{2}}} \big |f_k-g_k\big |$, $\sum _{CN^{\frac{\kappa }{2}}<|k|\le N} \big |f_k-g_k\big |$ and $\sum _{|k|>N} \big |f_k-g_k\big |$, where $C>0$. First of all observe that we have

$$\begin{aligned} f_k&=A_k R\! \left[ \left( \frac{B_k}{A_k}\right) ^2\right] +\frac{(B_k)^2(A_k-|k|^2)}{2|k|^2 A_k},\\ g_k&=\left( |k|^2+8\pi {\mathfrak {a}} N^\kappa \right) R\! \left[ \left( \frac{8\pi {\mathfrak {a}} N^\kappa }{|k|^2+8\pi {\mathfrak {a}} N^\kappa }\right) ^2\right] +\frac{(8\pi {\mathfrak {a}} N^\kappa )^3}{2|k|^2 \left( |k|^2+8\pi {\mathfrak {a}} N^\kappa \right) }, \end{aligned}$$

with the definition of the Taylor residuum $R[x]:=\sqrt{1-x}-1+\frac{x}{2}$. By Lemma 1 we have the estimates $|A_k-|k|^2|\lesssim N^{\kappa }$ and $|B_k|\lesssim N^\kappa$ and therefore we obtain

$$\begin{aligned} \sum _{|k|>N} \big |f_k-g_k\big |\lesssim \sum _{|k|>N} \left( \big |f_k\big |+\big |g_k\big |\right) \lesssim \sum _{|k|>N} \frac{N^{3\kappa }}{|k|^4}\lesssim N^{3\kappa -1}, \end{aligned}$$

where we have used $|R[x]|\lesssim |x|^2$. Furthermore, we have

$$\begin{aligned} \left| |k|^2+8\pi {\mathfrak {a}} N^{\kappa }-2A_k\right|&\lesssim N^{2\kappa -1}(1+|k|)+N^{\kappa }\mathbbm {1}(|k|>K) ,\\ \left| 8\pi {\mathfrak {a}} N^{\kappa }-2B_k\right|&\lesssim N^{2\kappa -1}(1+|k|) \end{aligned}$$

by Lemma 1, and as a consequence

$$\begin{aligned} \big |\frac{(B_k)^2(A_k-|k|^2)}{2|k|^2 A_k}-\frac{(8\pi {\mathfrak {a}} N^\kappa )^3}{2|k|^2 \left( |k|^2+8\pi {\mathfrak {a}} N^\kappa \right) }\big |\lesssim \frac{N^{4\kappa }}{N|k|^3}+\frac{N^{3\kappa }\mathbbm {1}(|k|>K)}{|k|^4}. \end{aligned}$$

Defining $x:=\left( \frac{B_k}{A_k}\right) ^2$ as well as $y:=\left( \frac{8\pi {\mathfrak {a}} N^\kappa }{|k|^2+8\pi {\mathfrak {a}} N^\kappa }\right) ^2$ we have the similar estimate

$$\begin{aligned} |x-y|\lesssim \frac{N^{3\kappa }}{N|k|^3}+\frac{N^{2\kappa }\mathbbm {1}(|k|>K)}{|k|^4}, \end{aligned}$$

and hence we can bound $\big |A_k R[x] \! - \! \left( |k|^2 \! + \! 8\pi {\mathfrak {a}} N^\kappa \right) R[y]\big |$ from above by

$$\begin{aligned}&\big |(A_k \! - \! |k|^2-8\pi {\mathfrak {a}} N^\kappa ) R[x] \big | \! + \! \left( |k|^2 \! + \! 8\pi {\mathfrak {a}} N^\kappa \right) \! \big | R[x] \! - \! R[y]\big |\\&\quad \lesssim \left( N^{2\kappa -1}|k|+N^{\kappa }\mathbbm {1}(|k|>K)\right) \frac{N^{4\kappa }}{|k|^8}+|k|^2 \frac{N^{2\kappa }}{|k|^4}\left( \frac{N^{3\kappa }}{N|k|^3}+\frac{N^{2\kappa }\mathbbm {1}(|k|>K)}{|k|^4}\right) \\&\quad \lesssim \frac{N^{5\kappa }}{N|k|^5}+\frac{N^{4\kappa }\mathbbm {1}(|k|>K)}{|k|^6} \end{aligned}$$

for $|k|>CN^{\frac{\kappa }{2}}$ and *C* large enough such that $x,y\le \frac{1}{2}$, where we have used that

$$\begin{aligned} \big | R[x] \! - \! R[y]\big |\lesssim \max \{x,y\}|y-x| \end{aligned}$$

for such *x* and *y*. Consequently,

$$\begin{aligned} \sum _{CN^{\frac{\kappa }{2}}<|k|\le N} \big |f_k-g_k\big |\lesssim \frac{N^{4\kappa }\log N}{N}+\frac{N^{3\kappa }}{K}. \end{aligned}$$

Regarding the final term $\sum _{0<|k|\le CN^{\frac{\kappa }{2}}} \big |f_k-g_k\big |$, observe that

$$\begin{aligned} \bigg |\sqrt{A_k^2-B_k^2}-\sqrt{|k|^4+16\pi {\mathfrak {a}} N^\kappa |k|^2}\bigg |\lesssim \frac{\big |A_k^2-B_k^2-|k|^4-16\pi {\mathfrak {a}} N^\kappa |k|^2\big |}{\sqrt{|k|^4+16\pi {\mathfrak {a}} N^\kappa |k|^2}}\lesssim \frac{N^{3\kappa -1}}{N^{\frac{\kappa }{2}} |k|}. \end{aligned}$$

Furthermore, we have $\big ||k|^2+8\pi {\mathfrak {a}} N^\kappa -\frac{(8\pi {\mathfrak {a}} N^\kappa )^2}{2|k|^2}-A_k+C_k\big |\lesssim \frac{N^{3\kappa }}{N |k|}$, and therefore we conclude with the estimate $\sum _{0<|k|\le CN^{\frac{\kappa }{2}}} \big |f_k-g_k\big |\lesssim \sum _{0<|k|\le CN^{\frac{\kappa }{2}}} \frac{N^{3\kappa }}{N |k|}\lesssim N^{4\kappa -1}$. $\square$

While it is clear by Lemma 1 that the coefficients $\nu _k$,respectively $f_{\ell ,\ell -k}$, introduced in Sect. 4, respectively Lemma 4, are bounded from above by $\frac{N^{\kappa }}{|k|^2}$, the following Lemma 14 shows that the decay in *k* is even stronger. A similar result holds for the coefficients $\psi _p(\ell )$ defined below Eq. (87).

### Lemma 14

There exists a constant $C>0$, depending uniformly on *V* in the sense of Eq. (12), such that we have $\sum _{k\ne 0} |k|^2 \nu _k^2\le C N^{1+\kappa }$ as well as $\sum _{k\ne 0} |k|^2 f_{\ell ,\ell -k}^2\le C N^{\kappa -1}$ for all $\ell$. Furthermore, $\sum _{\ell \ne 0}|\ell |^2 |\psi _p(\ell )|^2\le \frac{C}{L}$.

### Proof

Let us define $\varphi _{\ell }:=V_{N^{1-\kappa }}(e^{i\ell x}+e^{i\ell y})$ for $|\ell |<K$. Then,

$$\begin{aligned} \sum _{k\ne 0} |k|^2 f_{\ell ,\ell -k}^2 \!&\le \! \sum _k \left( |k|^2 \! + \! |\ell \! - \! k|^{2}\right) (f_{\ell ,\ell -k})^2 = {\langle {R\varphi _{\ell },(-\Delta _2)R\varphi _{\ell } }\rangle } \! \\  &\le \! {\langle {R\varphi _{\ell },(-\Delta _2+V_{N^{1-\kappa }})R\varphi _{\ell } }\rangle } \! , \end{aligned}$$

and furthermore by Lemma 1

90 $$\begin{aligned} {\langle {R\varphi _{\ell },(-\Delta _2 \! + \! V_{N^{1-\kappa }})R\varphi _{\ell } }\rangle } \!&= \! {\langle {\varphi _{\ell },R\varphi _{\ell } }\rangle } \! = \! 2\left\{ (V_{N^{1-\kappa }} R V_{N^{1-\kappa }})_{\ell 0 ,\ell 0} \! \right. \nonumber \\  &\quad \left. + (V_{N^{1-\kappa }} R V_{N^{1-\kappa }})_{\ell 0 ,0 \ell }\right\} \! \lesssim \! N^{\kappa -1}. \end{aligned}$$

The estimate on $\sum _{\ell \ne 0}|\ell |^2 |\psi _p(\ell )|^2$ can be derived in the same way using $\psi _p=R_p {\widetilde{\varphi }}$ with ${\widetilde{\varphi }}:=V_L$. Regarding $\nu _k$ note that $|\nu _k|\lesssim |k|^{-2}B_k=N{\langle {e^{ik(x-y)},R\varphi _0}\rangle }$, and therefore

$$\begin{aligned} \sum _{k\ne 0} |k|^2 \nu _k^2\lesssim N^2 {\langle {R\varphi _{\ell },(-\Delta _2)R\varphi _{\ell } }\rangle }. \end{aligned}$$

Applying Eq. (90) for $\ell =0$ concludes the proof. $\square$

## Additional Error Estimates

In this Section we will discuss estimates which will allow us, together with the results in Sect. 4.1, to control the error term $\sum _{i=1}^4 {\mathcal {E}}_i$.

### Lemma 15

Let $0<r<1$. Then there exists a constant $C>0$, depending uniformly on *V* in the sense of Eq. (12), such that

91 $$\begin{aligned} \pm {\mathcal {E}}_4\Big |_{{\mathcal {F}}^+_{M}}\le C N^{\frac{11\kappa }{2}-1}\left( \widetilde{{\mathcal {N}}}\Big |_{{\mathcal {F}}^+_{M}}+1\right) \end{aligned}$$

for all $M\le \min \{rN,N-1\}$. Furthermore

$$\begin{aligned} \pm \sum _k |k|^2 |w_k|^2\big ([\delta _k,d_k^\dagger ]+[d_k,\delta _k^\dagger ]+[\delta _k,\delta _k^\dagger ]\big )\Big |_{{\mathcal {F}}^+_{M}}\le C N^{\frac{11\kappa }{2}-1}\left( \widetilde{{\mathcal {N}}}\Big |_{{\mathcal {F}}^+_{M}}+1\right) . \end{aligned}$$

### Proof

Recall the representation of the error term ${\mathcal {E}}_4|_{{\mathcal {F}}^+_{N-1}}$ from Lemma 5

$$\begin{aligned} \frac{1}{2}\sum _{k\ne 0}\left\{ A_k-\sqrt{A_k^2-B_k^2}-C_k\right\} \left( \left[ \delta _k,a_k^\dagger \frac{1}{\sqrt{a_0 a_0^\dagger }}a_0\right] +\left[ a_0^\dagger \frac{1}{\sqrt{a_0 a_0^\dagger }}a_k,\delta _k^\dagger \right] +\left[ \delta _k,\delta _k^\dagger \right] \right) \Bigg |_{{\mathcal {F}}^+_{N-1}} \end{aligned}$$

and compute $\left[ \delta _k,a_k^\dagger \frac{1}{\sqrt{a_0 a_0^\dagger }}a_0\right] +\left[ a_0^\dagger \frac{1}{\sqrt{a_0 a_0^\dagger }}a_k,\delta _k^\dagger \right] =\left( T_1+T_2+\mathrm {H.c.}\right)$ with

$$\begin{aligned} T_1&:= \frac{a_0}{\sqrt{N}}\left( \frac{1}{\sqrt{a_0^\dagger a_0}}\sqrt{a_0 a_0^\dagger }-\frac{1}{\sqrt{a_0 a_0^\dagger }}\sqrt{a^\dagger _0 a_0}\right) \frac{a_0}{\sqrt{N}}w_k a_{-k}^\dagger a_k^\dagger ,\\ T_2&:= \frac{1}{\sqrt{a_0 a_0^\dagger }}\left( \sqrt{a_0^\dagger a_0} \! - \! \sqrt{a_0 a_0^\dagger }\right) \! \frac{a_0 a^\dagger _k}{N}\sum _{|\ell |<K}N\left( \left( T \! - \! 1\right) _{(\ell -k)k, \ell 0} \! \right. \\  &\quad \left. + \! \left( T-1\right) _{(\ell -k)k, 0 \ell }\right) a^{\dagger }_{\ell -k}a_\ell . \end{aligned}$$

Using that $\frac{a_0}{\sqrt{N}}\left( \frac{1}{\sqrt{a_0^\dagger a_0}}\sqrt{a_0 a_0^\dagger }-\frac{1}{\sqrt{a_0 a_0^\dagger }}\sqrt{a^\dagger _0 a_0}\right) \frac{a_0}{\sqrt{N}}$ and $\frac{1}{\sqrt{a_0 a_0^\dagger }}\left( \sqrt{a_0^\dagger a_0} \! - \! \sqrt{a_0 a_0^\dagger }\right) \! \frac{a_0 a^\dagger _k}{N}$ is bounded by $\frac{C_r}{N}$ on ${\mathcal {F}}^+_{rN}$, we immediately obtain $(T_1+T_1^\dagger )|_{{\mathcal {F}}^+_{rN}}\lesssim N^{\kappa -1}({\mathcal {N}}+1)$ from $\sum _{k\ne 0}|w_k|^2\lesssim N^{\kappa }$ and following the proof of Lemma 4 we furthermore obtain $(T_2+T_2^\dagger )|_{{\mathcal {F}}^+_{rN}} \lesssim N^{\kappa -1}{\mathcal {N}}$. In a similar fashion, one arrives at the estimate $\left[ \delta _k,\delta _k^\dagger \right] \big |_{{\mathcal {F}}^+_{rN}}\lesssim N^{2\kappa -1}({\mathcal {N}}+1)$. This concludes the proof of Eq. (91), since $\left| A_k-\sqrt{A_k^2-B_k^2}-C_k\right| \lesssim N^{2\kappa }$ by Lemma 1 and therefore

$$\begin{aligned} \pm {\mathcal {E}}_4\Big |_{{\mathcal {F}}^+_{M}}\lesssim N^{4\kappa -1}{\mathcal {N}}\Big |_{{\mathcal {F}}^+_{M}}\lesssim N^{\frac{11\kappa }{2}-1}\left( \widetilde{{\mathcal {N}}}\Big |_{{\mathcal {F}}^+_{M}}+1\right) , \end{aligned}$$

where we have used Lemma 16. The second part of the Lemma can be verified analogously. $\square$

The following Lemma 16 will allow us to compare terms in the variables $a_k$ with terms in the new variables $b_k$.

### Lemma 16

There exists a constant $C>0$, depending uniformly on *V* in the sense of Eq. (12), such that ${\mathcal {N}}\le C N^{\frac{\kappa }{2}}\widetilde{{\mathcal {N}}}+CN^{\frac{3\kappa }{2}}$, and for $0<\delta \le 1$ and $\ell \in {\mathbb {N}}$

$$\begin{aligned} \left( \sum _{k\ne 0}{\mathfrak {a}}_k^\dagger {\mathfrak {a}}_k+1\right) ^\ell \! \! \! \left( \sum _{k}|k|^{1+\delta } {\mathfrak {a}}_k^\dagger {\mathfrak {a}}_k+1\right) \!&\le C \! N^{\left( \frac{3\ell }{2}+2-\delta \right) \kappa +\delta } \! \left( \sum _{k\ne 0}{\mathfrak {b}}_k^\dagger {\mathfrak {b}}_k+1\right) ^\ell \! \! \! \left( \sum _{k}|k|^{1+\delta } {\mathfrak {b}}_k^\dagger {\mathfrak {b}}_k+1\right) \! ,\\ \left( \sum _{k\ne 0}{\mathfrak {b}}_k^\dagger {\mathfrak {b}}_k+1\right) ^\ell \! \! \! \left( \sum _{k}|k|^{1+\delta } {\mathfrak {b}}_k^\dagger {\mathfrak {b}}_k+1\right) \!&\le C \! N^{\left( \frac{3\ell }{2}+2-\delta \right) \kappa +\delta } \! \left( \sum _{k\ne 0}{\mathfrak {a}}_k^\dagger {\mathfrak {a}}_k+1\right) ^\ell \! \! \! \left( \sum _{k}|k|^{1+\delta } {\mathfrak {a}}_k^\dagger {\mathfrak {a}}_k+1\right) . \end{aligned}$$

The same estimate holds when we exchange $\sum _{k}|k|^{1+\delta } {\mathfrak {b}}_k^\dagger {\mathfrak {b}}_k$ with ${\mathbb {K}}$ defined in Lemma 10 and $\sum _{k}|k|^{1+\delta } {\mathfrak {a}}_k^\dagger {\mathfrak {a}}_k$ with $\sum _{k}|k|^{2} {\mathfrak {a}}_k^\dagger {\mathfrak {a}}_k+\frac{1}{2}\sum _{k\ell ,mn}(V_{N^{1-\kappa }})_{\ell k,mn}{\mathfrak {a}}_k^\dagger {\mathfrak {a}}_\ell ^\dagger {\mathfrak {a}}_m {\mathfrak {a}}_n$. Furthermore,

$$\begin{aligned} \left( {\mathcal {N}}+1\right) ^\ell \! \left( \sum _{k}|k|^{1+\delta } a_k^\dagger a_k+1\right) \big |_{{\mathcal {F}}^+_{M_\ell }} \! \le C \! N^{\left( \frac{3\ell }{2}+2-\delta \right) \kappa +\delta } \! \left( \widetilde{{\mathcal {N}}}+1\right) ^\ell \! \left( \sum _{k}|k|^{1+\delta } b_k^\dagger b_k+1\right) \big |_{{\mathcal {F}}^+_{M_\ell }} \end{aligned}$$

with the definition $M_\ell :=N-2(\ell +1)$.

### Proof

Using $a_k=\frac{1}{\sqrt{a_0 a_0^\dagger }}a_0\gamma _k b_k+\frac{1}{\sqrt{a_0 a_0^\dagger }}a_0\nu _{-k}b_{-k}^\dagger$, we can rewrite

$$\begin{aligned} {\mathcal {N}}=\sum _{k\ne 0}\left( \gamma _k b_k+\nu _{-k}b_{-k}^\dagger \right) ^\dagger \left( \gamma _k b_k+\nu _{-k}b_{-k}^\dagger \right) \lesssim \sum _{k\ne 0}\left( \gamma _k^2+\nu _k^2\right) b_k^\dagger b_k+\sum _{k\ne 0}\nu _k^2. \end{aligned}$$

Since $\sum _{k\ne 0}\nu _k^2\lesssim N^{\frac{3\kappa }{2}}$ and $|\nu _k|\le |\gamma _k|\lesssim N^{\frac{\kappa }{4}}$, we obtain ${\mathcal {N}}\Big |_{{\mathcal {F}}^+_{N-1}}\lesssim N^{\frac{\kappa }{2}}\widetilde{{\mathcal {N}}}\Big |_{{\mathcal {F}}^+_{N-1}}+N^{\frac{3\kappa }{2}}$. For the other statements recall that ${\mathfrak {a}}_k=\gamma _k {\mathfrak {b}}_k+\nu _{-k}{\mathfrak {b}}_{-k}^\dagger$, and let us define the vector $\nu ^\delta _k:=|k|^{\frac{1+\delta }{2}}\nu _k$. Then we can estimate $\left( \sum _{k\ne 0}{\mathfrak {a}}_k^\dagger {\mathfrak {a}}_k+1\right) ^\ell \! \! \! \left( \sum _{k}|k|^{1+\delta } {\mathfrak {a}}_k^\dagger {\mathfrak {a}}_k+1\right)$ from above by

$$\begin{aligned}&C_{\ell } \max \{\Vert \nu \Vert ^2,\Vert \gamma \Vert ^2_\infty \}^\ell \max \{\Vert \nu ^\delta \Vert ^2,\Vert \gamma \Vert ^2_\infty \}\left( \sum _{k\ne 0}{\mathfrak {b}}_k^\dagger {\mathfrak {b}}_k+1\right) ^\ell \! \! \! \left( \sum _{k}|k|^{1+\delta } {\mathfrak {b}}_k^\dagger {\mathfrak {b}}_k+1\right) \\&\quad \lesssim N^{\left( \frac{3\ell }{2}+2-\delta \right) \kappa +\delta } \! \left( \sum _{k\ne 0}{\mathfrak {b}}_k^\dagger {\mathfrak {b}}_k+1\right) ^\ell \! \! \! \left( \sum _{k}|k|^{1+\delta } {\mathfrak {b}}_k^\dagger {\mathfrak {b}}_k+1\right) . \end{aligned}$$

for a suitable constant $C_{\ell }$, where we have used $\Vert \nu \Vert ^2=\sum _{k\ne 0}\nu _k^2\lesssim N^{\frac{3\kappa }{2}}$, $\Vert \gamma \Vert ^2_\infty \lesssim N^{\frac{\kappa }{2}}$ and $\Vert \nu ^\delta \Vert ^2\lesssim N^{2\kappa +\delta (1-\kappa )}$. The same statement holds for the operator ${\mathfrak {a}}_k$ exchanged with ${\mathfrak {b}}_k$, since ${\mathfrak {b}}_k=\gamma _k {\mathfrak {a}}_k+\nu _{k}{\mathfrak {a}}_{-k}^\dagger$. The final statement follows from the fact that

$$\begin{aligned} \left( {\mathcal {N}}+1\right) ^\ell \! \left( \sum _{k}|k|^{1+\delta } a_k^\dagger a_k+1\right) \Psi =\left( \sum _{k\ne 0}{\mathfrak {a}}_k^\dagger {\mathfrak {a}}_k+1\right) ^\ell \! \! \! \left( \sum _{k}|k|^{1+\delta } {\mathfrak {a}}_k^\dagger {\mathfrak {a}}_k+1\right) \Psi \end{aligned}$$

for $$\Psi \in {\mathcal {F}}^+_{N-1}$$, and

$$\begin{aligned} \left( \widetilde{{\mathcal {N}}}+1\right) ^\ell \! \left( \sum _{k}|k|^{1+\delta } b_k^\dagger b_k+1\right) \Psi =\left( \sum _{k\ne 0}{\mathfrak {b}}_k^\dagger {\mathfrak {b}}_k+1\right) ^\ell \! \! \! \left( \sum _{k}|k|^{1+\delta } {\mathfrak {b}}_k^\dagger {\mathfrak {b}}_k+1\right) \Psi \end{aligned}$$

for $\Psi \in {\mathcal {F}}^+_{M_\ell }$, where we have used $b_k^\dagger b_k {\mathcal {F}}^+_{M}\subset {\mathcal {F}}^+_{M+2}$. $\square$

While the most prominent error terms appearing in the proof of Theorem 2 are being estimated in Sect. 4.1, the following Lemma 17 provides good estimates on the residual terms, which will furthermore be important for the proof of Theorem 3.

### Lemma 17

Let $0<\lambda _0<1-4\kappa$ and $\frac{5\kappa }{2}<\lambda <1-\frac{3\kappa }{2}$. Then there exists a constant $C>0$, depending uniformly on *V* in the sense of Eq. (12), such that

92 $$\begin{aligned}&b_k^\dagger b_k\Big |_{{\mathcal {F}}^{\le }_{N^{\lambda _0}}\cap {\mathcal {F}}^+_{N^\lambda }}-\left( \gamma _k d_k+\nu _k d_k^\dagger \right) ^\dagger \left( \gamma _k d_k+\nu _k d_k^\dagger \right) \Big |_{{\mathcal {F}}^{\le }_{N^{\lambda _0}}\cap {\mathcal {F}}^+_{N^\lambda }} \nonumber \\&\quad \le C \left( N^{2\kappa +\frac{\lambda _0}{2}-\frac{1}{2}}+N^{\frac{3\kappa }{2}+\lambda -1}\right) \left( b_k^\dagger b_k+\frac{\widetilde{{\mathcal {N}}}+1}{|k|^4}\right) \Big |_{{\mathcal {F}}^{\le }_{N^{\lambda _0}}\cap {\mathcal {F}}^+_{N^\lambda }} , \end{aligned}$$

and there exists a constant $K_0$ such that for $K\ge K_0 N^{\frac{\kappa }{2}}$ and $\delta >0$

93 $$\begin{aligned} \sum _{k}|k|^{1-\delta }b_k^\dagger b_k\Big |_{{\mathcal {F}}^{\le }_{N^{\lambda _0}}\cap {\mathcal {F}}^+_{N^\lambda }}\le C N^{-\frac{\kappa }{2}}\sum _{k\ne 0}e_k\left( \gamma _k d_k+\nu _k d_k^\dagger \right) ^\dagger \left( \gamma _k d_k+\nu _k d_k^\dagger \right) \Big |_{{\mathcal {F}}^{\le }_{N^{\lambda _0}}\cap {\mathcal {F}}^+_{N^\lambda }}{+}1. \end{aligned}$$

Regarding the error terms $F_1$ and $F_2$ introduced below Eq. (47) we have the estimates

94 $$\begin{aligned} \pm \left( F_1+F_1^\dagger \right) \bigg |_{{\mathcal {F}}^{\le }_{N^{\lambda _0}}\cap {\mathcal {F}}^+_{N^\lambda }}&\le C N^{\frac{5\kappa }{2}+\lambda -1}\left( \widetilde{{\mathcal {N}}}\Big |_{{\mathcal {F}}^{\le }_{N^{\lambda _0}}\cap {\mathcal {F}}^+_{N^\lambda }}+1\right) , \end{aligned}$$

95 $$\begin{aligned} \pm F_2 \Big |_{{\mathcal {F}}^{\le }_{N^{\lambda _0}}\cap {\mathcal {F}}^+_{N^\lambda }}&\le C \left( N^{\frac{9\kappa }{2}+\lambda _0-1}+N^{\frac{7\kappa }{2}+2\lambda -2}\right) \left( \widetilde{{\mathcal {N}}}\Big |_{{\mathcal {F}}^{\le }_{N^{\lambda _0}}\cap {\mathcal {F}}^+_{N^\lambda }}+1\right) , \end{aligned}$$

and $$\pm \left( F_1+F_1^\dagger +F_2\right) \le C N^{6\kappa -1}\! \left( \widetilde{{\mathcal {N}}}+1\right) ^3$$. Further, $${\mathcal {N}}({\mathcal {N}}+1)\Big |_{{\mathcal {F}}^+_{N^\lambda }} \le C 2N^{\frac{\kappa }{2}+\lambda } \left( \widetilde{{\mathcal {N}}}\Big |_{{\mathcal {F}}^+_{N^\lambda }}+1\right) $$.

### Proof

In order to verify the Lemma, let us first show

96 $$\begin{aligned} \left\{ (\delta _k)^\dagger \delta _k+ \delta _k (\delta _k)^\dagger \right\} \Big |_{{\mathcal {F}}^{\le }_{N^{\lambda _0}}\cap {\mathcal {F}}^+_{N^\lambda }}\lesssim |k|^{-4}N^{\frac{5\kappa }{2}}\left( N^{\kappa +\lambda _0-1}+N^{2\lambda -2}\right) \left( \widetilde{{\mathcal {N}}}\Big |_{{\mathcal {F}}^{\le }_{N^{\lambda _0}}\cap {\mathcal {F}}^+_{N^\lambda }}+1\right) . \end{aligned}$$

For this purpose, we follow the proof of Lemma 4 by writing $\delta _k=-\delta _k'+\delta _k''$ and estimating

$$\begin{aligned}&\left\{ (\delta _k'')^\dagger \delta _k''+ \delta _k'' (\delta _k'')^\dagger \right\} \Big |_{{\mathcal {F}}^+_{N^\lambda }}\lesssim N^{2\kappa -2}|k|^{-4}\left( {\mathcal {N}}+1\right) ^3\\&\quad \lesssim N^{2\kappa -2}|k|^{-4}\Big (\sum _{m,n,p\ne 0} a_m^\dagger a_n^\dagger a_p^\dagger a_p a_n a_m+1\Big ). \end{aligned}$$

By expressing $a_p$ in terms of $b_p$ and $b_{-p}^\dagger$, we furthermore obtain

97 $$\begin{aligned} \sum _{m,n,p\ne 0} a_m^\dagger a_n^\dagger a_p^\dagger a_p a_n a_m\Big |_{{\mathcal {F}}^+_{N^\lambda }} \! \! \!&\lesssim \! \sum _{m,n,p\ne 0} (\gamma _p^2 \! + \! \nu _p^2) a_m^\dagger a_n^\dagger b_p^\dagger b_p a_n a_m\Big |_{{\mathcal {F}}^+_{N^\lambda }} \! \! \nonumber \\  &\quad + \! \sum _{m,n,p\ne 0} \nu _p^2 a_m^\dagger a_n^\dagger a_n a_m\Big |_{{\mathcal {F}}^+_{N^\lambda }}\nonumber \\  &\lesssim \sum _{m,n,p\ne 0} (\gamma _p^2 \! + \! \nu _p^2) b_p^\dagger a_m^\dagger a_n^\dagger a_n a_m b_p \Big |_{{\mathcal {F}}^+_{N-1}} \nonumber \\  &\quad + \! \! \! \sum _{m,n,p\ne 0} \! \! \! (\gamma _p^2 \! + \! \nu _p^2) [a_n a_m, b_p]^\dagger [a_n a_m, b_p] \Big |_{{\mathcal {F}}^+_{N^\lambda }} \! \! \nonumber \\  &\quad +\! \! \sum _{m,n,p\ne 0} \nu _p^2 a_m^\dagger a_n^\dagger a_n a_m\Big |_{{\mathcal {F}}^+_{N^\lambda }}\nonumber \\  &\lesssim N^{\frac{\kappa }{2}}\sum _{p\ne 0} b_p^\dagger {\mathcal {N}}^2 b_p \Big |_{{\mathcal {F}}^+_{N^\lambda }}+N^{2\kappa }{\mathcal {N}}\Big |_{{\mathcal {F}}^ +_{N^\lambda }}+N^{\frac{3\kappa }{2}}{\mathcal {N}}^2\Big |_{{\mathcal {F}}^+_{N^\lambda }}\nonumber \\  &\lesssim N^{\frac{\kappa }{2}+2\lambda }\widetilde{{\mathcal {N}}}\Big |_{{\mathcal {F}}^+_{N^\lambda }} +N^{\frac{7\kappa }{2}}\left( \widetilde{{\mathcal {N}}}\Big |_{{\mathcal {F}}^+_{N^\lambda }}+1\right) \nonumber \\  &\quad +N^{3\kappa +\lambda }\left( \widetilde{{\mathcal {N}}}\Big |_{{\mathcal {F}}^+_{N^\lambda }}+1\right) . \end{aligned}$$

Since we assume $\lambda >\frac{5\kappa }{2}$, we obtain $\left\{ (\delta _k'')^\dagger \delta _k''+ \delta _k'' (\delta _k'')^\dagger \right\} \Big |_{{\mathcal {F}}^+_{N^\lambda }}\lesssim |k|^{-4} N^{\frac{5\kappa }{2}+2\lambda -2}\left( \widetilde{{\mathcal {N}}}+1\right)$.

Let us furthermore estimate $\left\{ (\delta _k')^\dagger \delta _k'+ \delta _k' (\delta _k')^\dagger \right\} \Big |_{{\mathcal {F}}^{\le }_{N^{\lambda _0}}\cap {\mathcal {F}}^+_{N^\lambda }}$ from above by

98 $$\begin{aligned}&|k|^{-4}N^{2\kappa -1}\left( \sum _{0<|\ell |,|\ell '|<K} a^\dagger _{\ell '-k}a_\ell ^\dagger a_\ell a_{\ell '-k}+\sum _{p\ne 0} a_p^\dagger a_p\right) \Big |_{{\mathcal {F}}^{\le }_{N^{\lambda _0}}\cap {\mathcal {F}}^+_{N^\lambda }}\nonumber \\&\quad \lesssim \! |k|^{-4}N^{2\kappa -1}\left( \! N^{\lambda _0}\sum _{p\ne 0}a^\dagger _{p} a_{p}\! +\! N^{\frac{3\kappa }{2}}\left( \widetilde{{\mathcal {N}}}+1\right) \! \! \! \right) \Big |_{{\mathcal {F}}^{\le }_{N^{\lambda _0}}\cap {\mathcal {F}}^+_{N^\lambda }}\nonumber \\&\quad \lesssim \! |k|^{-4}N^{\frac{7\kappa }{2}+\lambda _0-1}\! \! \left( \! \widetilde{{\mathcal {N}}}\Big |_{{\mathcal {F}}^{\le }_{N^{\lambda _0}}\cap {\mathcal {F}}^+_{N^\lambda }}\! +\! 1\! \right) \! . \end{aligned}$$

Combining the estimates on $\delta '_k$ and $\delta ''_k$ therefore yields Eq. (96). In order to verify Eq. (93) note that $b_k-\gamma _k d_k-\nu _k d_{-k}^\dagger =\gamma _k \delta _k+\nu _k \delta _{-k}^\dagger$. Using Eq. (96), as well as the observation that $|k|^{1-\delta } \lesssim N^{-\frac{\kappa }{2}}e_k$ for $K\ge K_0 N^{\frac{\kappa }{2}}$ and $K_0$ large enough, yields

$$\begin{aligned} \sum _{k}|k|^{1-\delta }b_k^\dagger b_k\Big |_{{\mathcal {F}}^{\le }_{N^{\lambda _0}}\cap {\mathcal {F}}^+_{N^\lambda }}&\lesssim N^{-\frac{\kappa }{2}}\sum _{k\ne 0}e_k\left( \gamma _k d_k+\nu _k d_k^\dagger \right) ^\dagger \left( \gamma _k d_k+\nu _k d_k^\dagger \right) \Big |_{{\mathcal {F}}^{\le }_{N^{\lambda _0}}\cap {\mathcal {F}}^+_{N^\lambda }}\\&\quad +N^{\frac{\kappa }{2}}\sum _k |k|^{1-\delta }(\delta _k^\dagger \delta _k+\delta _k \delta _k^\dagger )\Big |_{{\mathcal {F}}^{\le }_{N^{\lambda _0}}\cap {\mathcal {F}}^+_{N^\lambda }}\\&\lesssim \! N^{-\frac{\kappa }{2}}\! \sum _{k\ne 0}\! e_k \! \left( \gamma _k d_k \! + \! \nu _k d_k^\dagger \right) ^\dagger \! \left( \gamma _k d_k \! + \! \nu _k d_k^\dagger \right) \! \Big |_{{\mathcal {F}}^{\le }_{N^{\lambda _0}}\cap {\mathcal {F}}^+_{N^\lambda }} \\&\quad + \! N^{3\kappa } \! \! \left( N^{\kappa +\lambda _0-1} \! + \! N^{2\lambda -2} \right) \! \! \left( \! \widetilde{{\mathcal {N}}}\Big |_{{\mathcal {F}}^{\le }_{N^{\lambda _0}}\cap {\mathcal {F}}^+_{N^\lambda }} \! \! \! + \! 1 \! \right) \! \! . \end{aligned}$$

By our assumption $\lambda _0<1-4\kappa$ and $\lambda _0<1-\frac{3}{2}\kappa$ we have

$$\begin{aligned} \sum _{k}|k|^{1-\delta }b_k^\dagger b_k&\lesssim \left( 1-N^{3\kappa } \! \left( N^{\kappa +\lambda _0-1} \! + \! N^{2\lambda -2} \right) \right) \sum _{k}|k|^{1-\delta }b_k^\dagger b_k\\&\le \sum _{k}|k|^{1-\delta }b_k^\dagger b_k-N^{3\kappa } \! \left( N^{\kappa +\lambda _0-1} \! + \! N^{2\lambda -2} \right) \widetilde{{\mathcal {N}}}, \end{aligned}$$

which concludes the proof of Eq. (93). Note that Eq. (92) can be verified similarly. Furthermore, Eq. (95) follows immediately from Eq. (96), using the fact that $|k|^2 |w_k|\lesssim N^\kappa$. When it comes to Eq. (94), let us estimate in a similar fashion to Lemma 4

99 $$\begin{aligned} \pm \left( F_1+F_1^\dagger \right)&\lesssim N^{2\kappa -1}{\mathcal {N}}({\mathcal {N}} \! + \! 1)\nonumber \\&=N^{2\kappa -1}\sum _{p,q}a_p^\dagger a_q^\dagger a_q a_p+N^{2\kappa -1}\sum _p a_p^\dagger a_p\nonumber \\&\lesssim N^{\frac{5\kappa }{2}-1}\sum _{p,q}a_p^\dagger b_q^\dagger b_q a_p+N^{5\kappa -1}\! \left( \widetilde{{\mathcal {N}}}+1\right) \nonumber \\&\lesssim N^{\frac{5\kappa }{2}-1}\sum _{p,q}b_q^\dagger a_p^\dagger a_p b_q +N^{5\kappa -1}\! \left( \widetilde{{\mathcal {N}}}+1\right) , \end{aligned}$$

following the argument in Eq. (97). This immediately implies Eq. (94) as well as the bound $\pm \left( F_1+F_1^\dagger \right) \lesssim N^{5\kappa -1}\! \left( \widetilde{{\mathcal {N}}}+1\right) ^2$. Note that ${\mathcal {N}}^2\lesssim N^{3\kappa } \! \left( \widetilde{{\mathcal {N}}}+1\right) ^2$ by Eq. (99), and therefore we obtain by a similar argument as in Eq. (97) and Eq. (98) that

$$\begin{aligned} (\delta _k)^\dagger \delta _k+ \delta _k (\delta _k)^\dagger \lesssim |k|^{-4}N^{5\kappa -1} \! \left( \widetilde{{\mathcal {N}}}+1\right) ^3, \end{aligned}$$

and consequently $$\pm F_2\lesssim N^{6\kappa -1}\! \left( \widetilde{{\mathcal {N}}}+1\right) ^3$$. $$\square $$

## A Priori Condensation

In this subsection, we will use the strong estimates on the number of expected excited particles ${\langle {\Psi ,{\mathcal {N}}\Psi }\rangle }$ derived in [6], in order to obtain necessary ad hoc for our proof of Theorem 2. By [6, Theorem 1.2] we have the a priori estimate ${\langle {\Psi ,{\mathcal {N}}\Psi }\rangle }\le C N^{\frac{5\kappa }{2}}$ for states $\Psi$ satisfying

$$\begin{aligned} {\langle {\Psi ,H_{N,\kappa }\Psi }\rangle }-4\pi {\mathfrak {a}}N^{1+\kappa }\lesssim N^{\frac{5\kappa }{2}}. \end{aligned}$$

Making use of the fact that the Lee–Huang–Yang correction

$$\begin{aligned} \frac{1}{2} \! \sum _{k\ne 0} \! \left\{ \! \sqrt{|k|^4 \! + \! 16\pi {\mathfrak {a}} N^{\kappa }|k|^2} \! - \! |k|^2 \! - \! 8\pi {\mathfrak {a}} N^{\kappa } \! + \! \frac{(8\pi {\mathfrak {a}} N^{\kappa })^2}{2|k|^2} \! \right\} \end{aligned}$$

is of the order $N^{\frac{5\kappa }{2}}$, therefore yields

100 $$\begin{aligned} {\langle {\Psi _d,{\mathcal {N}}\Psi _d}\rangle }\le C N^{\frac{5\kappa }{2}} \end{aligned}$$

for any eigenstate $\Psi _d$ corresponding to an eigenvalue $E_{N,\kappa }^{(d)}$ with $E_{N,\kappa }^{(d)} - E_{N,\kappa }\lesssim N^{\frac{5\kappa }{2}}$. Note that the Eq. (100) only gives us an estimate on the expected number of particles. The following Lemma 18 however tells us that we can lift the control on the expected number of particles to a control on the number of particles in a spectral sense, i.e. we will argue that we can restrict our attention to states in the spectral subspace ${\mathcal {F}}^{\le }_{\lambda _0}\cap {\mathcal {F}}^{+}_\lambda$ without changing the energy significantly. Here we follow the methods presented in [7].

### Lemma 18

Let $E_{N,\kappa }^{(d)}$ satisfy $E_{N,\kappa }^{(d)}\le E_{N,\kappa }+CN^{\frac{\kappa }{2}}$ for a constant $C>0$ and $K\le N^{1+\kappa }$, and assume $\lambda _0>\frac{5\kappa }{2}$ as well as $\lambda >3\kappa$. Then there exists a *d* dimensional subspace ${\mathcal {V}}_d\subseteq {\mathcal {F}}^{\le }_{\lambda _0}\cap {\mathcal {F}}^{+}_\lambda$, such that for all elements $\Psi \in {\mathcal {V}}_d$ with $\Vert \Psi \Vert =1$

101 $$\begin{aligned} {\langle {\Psi ,H_{N,\kappa }\Psi }\rangle }\le E^{(d)}_{N,\kappa }+N^{-2\lambda _0} \! \left( N^{\frac{9\kappa }{2}}+KN^{2\kappa }\right) +N^{3\kappa -\lambda _0}+N^{-2\lambda }N^{1+\kappa }+N^{3\kappa -\lambda }. \end{aligned}$$

### Proof

Let ${\mathcal {W}}_d$ be the space spanned by the first *d* eigenfunctions of $H_{N,\kappa }$, $f,g:{\mathbb {R}}\longrightarrow [0,1]$ smooth functions satisfying $f^2+g^2=1$ and $f(x)=1$ for $x\le \frac{1}{2}$ as well as $f(x)=0$ for $x\ge 1$ and let ${\mathcal {N}}_*:=\sum _{0<|k|<K}a_k^\dagger a_k$. With this at hand we define the space $\widetilde{{\mathcal {V}}}_d:=f\!\left( \frac{{\mathcal {N}}_*}{N^{\lambda _0}}\right) \! {\mathcal {W}}_d$, which clearly satisfies $\widetilde{{\mathcal {V}}}_d\subseteq {\mathcal {F}}^{\le }_{\lambda _0}$. Note that by our assumption $\lambda _0>\frac{5\kappa }{2}$ and Eq. (100), we have

102 $$\begin{aligned} \left\| f\!\left( \frac{{\mathcal {N}}_*}{N^{\lambda _0}}\right) \Psi \right\| ^2\ge \left( 1-C N^{\frac{5\kappa }{2}-\lambda _0}\right) \Vert \Psi \Vert ^2>0 \end{aligned}$$

for any $\Psi \in {\mathcal {W}}_d\setminus \{0\}$, a suitable constant *C* and *N* large enough. Hence, it is clear that ${\widetilde{V}}_d$ is *d* dimensional again. Using the IMS identity

$$\begin{aligned} f \! \left( \frac{{\mathcal {N}}_*}{N^\lambda _0}\right) H_{N,\kappa }f \! \left( \frac{{\mathcal {N}}_*}{N^\lambda _0}\right) +g \! \left( \frac{{\mathcal {N}}_*}{N^\lambda _0}\right) H_{N,\kappa }g \! \left( \frac{{\mathcal {N}}_*}{N^\lambda _0}\right) =H_{N,\kappa }+{\mathcal {E}} \end{aligned}$$

with

$$\begin{aligned} {\mathcal {E}}:=\frac{1}{2}\left[ f \! \left( \frac{{\mathcal {N}}_*}{N^\lambda _0}\right) ,\left[ H_{N,\kappa },f \! \left( \frac{{\mathcal {N}}_*}{N^\lambda _0}\right) \right] \right] +\frac{1}{2}\left[ g \! \left( \frac{{\mathcal {N}}_*}{N^\lambda _0}\right) ,\left[ H_{N,\kappa },g \! \left( \frac{{\mathcal {N}}_*}{N^\lambda _0}\right) \right] \right] , \end{aligned}$$

see for example [7, 14], we obtain furthermore for a generic state $\Theta =\frac{f\!\left( \frac{{\mathcal {N}}_*}{N^{\lambda _0}}\right) \Psi }{\Vert f\!\left( \frac{{\mathcal {N}}_*}{N^{\lambda _0}}\right) \Psi \Vert }\in {\widetilde{V}}_d$, where $\Psi$ is a state in ${\mathcal {W}}_d$, and a suitable constant $C>0$ the estimate

$$\begin{aligned} {\langle {\Theta ,H_{N,\kappa }\Theta }\rangle }&\le \left\| f\!\left( \frac{{\mathcal {N}}_*}{N^{\lambda _0}}\right) \Psi \right\| ^{-2}\left( E_{N,\kappa }^{(d)} -{\langle {g\!\left( \frac{{\mathcal {N}}_*}{N^{\lambda _0}}\right) \Psi ,H_{N,\kappa }g\! \left( \frac{{\mathcal {N}}_*}{N^{\lambda _0}}\right) \Psi }\rangle }+{\langle {\Psi ,{\mathcal {E}}\Psi }\rangle }\right) \\&\le E_{N,\kappa }^{(d)}+C {\langle {\Psi ,{\mathcal {E}}\Psi }\rangle }+CN^{3\kappa -\lambda _0}, \end{aligned}$$

where we have used the lower bound

$$\begin{aligned} \left\langle g\!\left( \frac{{\mathcal {N}}_*}{N^{\lambda _0}}\right) \Psi ,H_{N,\kappa }g\!\left( \frac{{\mathcal {N}}_*}{N^{\lambda _0}} \right) \Psi \right\rangle&\ge \left( 1-\left\| f\!\left( \frac{{\mathcal {N}}_*}{N^{\lambda _0}}\right) \Psi \right\| ^2\right) E_{N,\kappa }\\&\ge \left( 1-\left\| f\!\left( \frac{{\mathcal {N}}_*}{N^{\lambda _0}}\right) \Psi \right\| ^2\right) E^{(d)}_{N,\kappa } -C N^{3\kappa -\lambda _0}, \end{aligned}$$

which follows from our assumptions on $E^{(d)}_{N,\kappa }$ together with Eq. (102). In order to estimate ${\langle {\Psi ,{\mathcal {E}}\Psi }\rangle }$, let us define $\pi _0$ as the projection onto the zero mode, $\pi _1$ as the projection onto the modes $\{k\in 2\pi {\mathbb {Z}}^3:0<|k|<K\}$ and $\pi _3$ as the projection onto $\{k\in 2\pi {\mathbb {Z}}^3:|k|>K\}$, and rewrite ${\mathcal {E}}$ as

$$\begin{aligned} {\mathcal {E}}=\frac{1}{4N^{2\lambda _0}}\sum _{I\in \{0,1,2\}^4} \sum _{jk,mn} (\pi _{I_1}\pi _{I_2} V_{N^{1-\kappa }} \pi _{I_3}\pi _{I_4})_{jk, mn} a^\dagger _k a_j^\dagger X_I a_m a_n, \end{aligned}$$

with

$$\begin{aligned} X_I \! \! :&= \! N^{2\lambda _0}\left[ f \! \left( \frac{{\mathcal {N}}_* \! + \! \#_{I_1,I_2}}{N^{\lambda _0}}\right) \! - \! f \! \left( \frac{{\mathcal {N}}_* \! + \! \#_{I_3,I_4}}{N^{\lambda _0}}\right) \right] ^2 \! + \! N^{2\lambda _0}\left[ g \! \left( \frac{{\mathcal {N}}_* \! + \! \#_{I_1,I_2}}{N^{\lambda _0}}\right) \!\right. \\  &\quad \left. - g \! \left( \frac{{\mathcal {N}}_* \! + \! \#_{I_3,I_4}}{N^{\lambda _0}}\right) \right] ^2 \end{aligned}$$

and $\#_{i,j}$ counts how many of the indices *i*, *j* are equal to 1. Before we start with the term-by-term analysis, let us introduce the variables ${\widetilde{c}}$ and ${\widetilde{\psi }}$ as the ones defined in Eq. (14) and Eq. (15) with the concrete choice of the cut-off parameter $0<K_0<2\pi$. Note that in this case, we obtain in analogy to Eq. (25) for a suitable $C>0$

$$\begin{aligned} H_{N,\kappa }-4\pi {\mathfrak {a}}_{N^{1-\kappa }}N^{\kappa }(N-1)\ge {\mathbb {P}}-CN^{\kappa }{\mathcal {N}}, \end{aligned}$$

where

$$\begin{aligned} {\mathbb {P}}:=\sum _k |k|^2 {\widetilde{c}}_k^\dagger {\widetilde{c}}_k+\frac{1}{2}\sum _{jk,mn\ne 0}\left( V_{N^{1-\kappa }}\right) _{jk,mn}{\widetilde{\psi }}^\dagger _{jk}{\widetilde{\psi }}_{mn}, \end{aligned}$$

which especially implies the upper bound ${\langle {\Psi ,{\mathbb {P}}\Psi }\rangle }\lesssim N^{\frac{7\kappa }{2}}$ for $\Psi \in {\mathcal {W}}_d$. Starting with the case $I_1=I_2=0$ and $I_3=I_4=1$, we identify $\sum _{|k|<K}(V_{N^{1-\kappa }})_{00,(-k)k}\left( a_0^{\dagger }\right) ^2 X_{I} a_{-k}a_k+\mathrm {H.c.}$ as

$$\begin{aligned}&\left( \sum _{|k|<K}(V_{N^{1-\kappa }})_{00,(-k)k}\left( a_0^{\dagger }\right) ^2 X_{I} {\widetilde{\psi }}_{(-k)k}-N^{-1}\sum _{|k|<K}(V_{N^{1-\kappa }})_{00,(-k)k}\left( a_0^{\dagger }\right) ^2 a_0^2 X_{I} w_k \right) \\&\quad +\mathrm {H.c.} \lesssim N^{\kappa }{\mathbb {P}}+KN^{2\kappa }, \end{aligned}$$

which gives an contribution of at most order $N^{\frac{9\kappa }{2}}+K N^{2\kappa }$ when evaluated against $\Psi$. Since we clearly only have to consider *I* for which $\#_{I_1,I_2}\ne \#_{I_3,I_4}$, the only relevant cases left are the ones where both $I_1,I_2$ and $I_3,I_4$ contain at least one non-zero index, and at least one of these index pairs contains the index 1. W.l.o.g., let us assume $I_1=1$. In this case

103 $$\begin{aligned}&\sum _{jk,mn} (\pi _{I_1}\pi _{I_2} V_{N^{1-\kappa }} \pi _{I_3}\pi _{I_4})_{jk, mn} a^\dagger _k a_j^\dagger X_I a_m a_n\nonumber \\  &\quad =\sum _{jk,mn} (\pi _{I_1}\pi _{I_2} V_{N^{1-\kappa }} \pi _{I_3}\pi _{I_4})_{jk, mn} a^\dagger _k a_j^\dagger X_I {\widetilde{\psi }}_{mn}\nonumber \\&\quad -N^{-1} a_0^2\sum _{jk,m} (\pi _{I_1}\pi _{I_2} V_{N^{1-\kappa }} \pi _{I_3}\pi _{I_4})_{jk, m(-m)} a^\dagger _k a_j^\dagger X_I w_m. \end{aligned}$$

Note that the second term on the right hand side of Eq. (103) can be treated in the same way as the case $I_1=I_2=0$ and $I_3=I_4=1$. Regarding the first term we estimate

$$\begin{aligned}&\sum _{jk,mn} \! \! (\pi _{I_1}\pi _{I_2} V_{N^{1-\kappa }} \pi _{I_3}\pi _{I_4})_{jk, mn} a^\dagger _k a_j^\dagger X_I {\widetilde{\psi }}_{mn} \! + \! \mathrm {H.c.} \! \\&\quad \lesssim \sum _{jk,mn} (\pi _{I_1}\pi _{I_2} V_{N^{1-\kappa }} \pi _{I_1}\pi _{I_2})_{jk, mn} a^\dagger _k a_j^\dagger a_m a_n+{\mathbb {P}}\\&\quad \lesssim \! {\mathbb {P}} \! + \! N^{-2} \! \left( a_0^\dagger \right) ^2 \! \! a_0^2 \! \! \! \sum _{|j|,|m|<K} \! \! \! \! \! (V_{N^{1-\kappa }} )_{j(-j), m(-m)}w_j w_m\\&\quad \lesssim {\mathbb {P}}+K^2 N^{\kappa -1}, \end{aligned}$$

which is of order $N^{\frac{7\kappa }{2}}+KN^{2\kappa }$ due to our assumption $K\le N^{1+\kappa }$. Therefore

$$\begin{aligned} {\langle {\Theta ,H_{N,\kappa }\Theta }\rangle }\le E^{(d)}_{N,\kappa }+N^{-2\lambda _0} \! \left( N^{\frac{9\kappa }{2}}+KN^{2\kappa }\right) +N^{3\kappa -\lambda _0} \end{aligned}$$

for any $\Theta \in \widetilde{{\mathcal {V}}}_d$ with $\Vert \Theta \Vert =1$. Note that states in $\Theta \in \widetilde{{\mathcal {V}}}_d$ still satisfy ${\langle {\Theta ,{\mathcal {N}}\Theta }\rangle }\lesssim N^{\frac{5\kappa }{2}}$, and let us further define ${\mathcal {V}}_d:=f\!\left( \frac{{\mathcal {N}}}{N^\lambda }\right) \! \widetilde{{\mathcal {V}}}_d$. Clearly ${\mathcal {V}}_d\subseteq {\mathcal {F}}^{\le }_{\lambda _0}\cap {\mathcal {F}}^{+}_\lambda$ and similar to before we see that ${\mathcal {V}}_d$ is indeed *d* dimensional. Making again use of the IMS identity, and performing similar estimates then yields for generic states $\Theta =\frac{f\!\left( \frac{{\mathcal {N}}}{N^\lambda }\right) \Psi }{\left\| f\!\left( \frac{{\mathcal {N}}}{N^\lambda }\right) \right\| }\in {\mathcal {V}}_d$, where $\Psi \in \widetilde{{\mathcal {V}}}_d$ with $\Vert \Psi \Vert =1$, and

$$\begin{aligned} {\langle {\Theta ,H_{N,\kappa }\Theta }\rangle }\le E^{(d)}_{N,\kappa }+N^{-2\lambda _0} \! \left( N^{\frac{9\kappa }{2}}+KN^{2\kappa }\right) +N^{3\kappa -\lambda _0}+N^{3\kappa -\lambda }+C{\langle {\Psi ,{\mathcal {E}}' \Psi }\rangle }, \end{aligned}$$

with

$$\begin{aligned} {\mathcal {E}}'&:=\frac{1}{4 N^{2\lambda }}\left( \sum _{k,\ell \ne 0}\widehat{V_{N,\kappa }}(k)a_k^\dagger a^\dagger _{\ell -k} X_0 a_\ell a_0+\mathrm {H.c.}\right) \\&\quad +\frac{1}{4N^{2\lambda }}\left( \sum _{k\ne 0}\widehat{V_{N,\kappa }}(k)a_k^\dagger a_{-k}^\dagger X_1 a_0^2+\mathrm {H.c.}\right) ,\\ X_i&:= N^{2\lambda }\left[ f \! \left( \frac{{\mathcal {N}}+2}{N^\lambda }\right) -f \! \left( \frac{{\mathcal {N}}+i}{N^{\lambda }}\right) \right] ^2+ N^{2\lambda }\left[ g \! \left( \frac{{\mathcal {N}}+2}{N^\lambda }\right) -g \! \left( \frac{{\mathcal {N}}+i}{N^\lambda }\right) \right] ^2. \end{aligned}$$

Using the fact that we have ${\mathcal {E}}'\lesssim N^{-2\lambda } \left( H_{N,\kappa }+N^{1+\kappa }\right)$ concludes the proof. $\square$

## References

[1] Boccato, C., Brennecke, C., Cenatiempo, S., Schlein, B.: Bogoliubov theory in the Gross–Pitaevskii limit. Acta Math. **222**, 219–335 (2019)

[2] Boccato, C., Seiringer, R.: The Bose gas in a box with Neumann boundary conditions. Ann. Henri Poincaré **24**, 1505–1560 (2023)

[3] Bogoliubov, N.: On the theory of superfluidity. Proc. Inst. Math. Kiev **9**, 89–103 (1947)

[4] Brennecke, C., Caporaletti, M., Schlein, B.: Excitation spectrum for Bose gases beyond the Gross–Pitaevskii regime. Rev. Math. Phys. **34**, 1–61 (2022)

[5] Dereziński, J., Napiórkowski, M.: Excitation spectrum of interacting bosons in the mean-field infinite-volume limit. Ann. Henri Poincaré **15**, 2409–2439 (2014)

[6] Fournais, S.: Length scales for BEC in the dilute Bose gas. In: Partial Differential Equations, Spectral Theory, and Mathematical Physics (2021)

[7] Fournais, S., Girardot, T., Junge, L., Morin, L., Olivieri, M.: Lower bounds on the energy of the Bose gas. Rev. Math. Phys. 36, 1–43 (2024) 10.1007/s00220-023-04907-2 PMC11231024 38983883

[8] Fournais, S., Solovej, J.: The energy of dilute Bose gases. Ann. Math. **192**, 893–976 (2020)

[9] Fournais, S., Solovej, J.: The energy of dilute Bose gases II: the general case. Invent. Math. **232**, 863–994 (2023)

[10] Haberberger, F., Hainzl, C., Nam, P., Seiringer, R., Triay, A.: The free energy of dilute Bose gases at low temperatures (2023). arXiv:2304.02405

[11] Hainzl, C.: Another proof of BEC in the GP-limit. J. Math. Phys. **62**, 1–16 (2021)

[12] Hainzl, C., Schlein, B., Triay, A.: Bogoliubov theory in the Gross–Pitaevskii limit: a simplified approach. Forum Math. Sigma **10**, 1–39 (2022)

[13] Lee, T., Huang, K., Yang, C.: Eigenvalues and eigenfunctions of a Bose system of hard spheres and its low-temperature properties. Phys. Rev. **106**, 1135–1145 (1957)

[14] Lewin, M., Nam, P., Serfaty, S., Solovej, J.: Bogoliubov spectrum of interacting Bose gases. Commun. Pure Appl. Math. **68**, 413–471 (2015)

[15] Lieb, E., Seiringer, R.: Proof of Bose–Einstein condensation for dilute trapped gases. Phys. Rev. Lett. **88**, 899–902 (2002) 10.1103/PhysRevLett.88.170409 12005741

[16] Lieb, E., Solovej, J.: Ground state energy of the one-component charged Bose gas. Commun. Math. Phys. **217**, 127–163 (2001)

[17] Lieb, E., Seiringer, R., Yngvason, J.: Bosons in a trap: a rigorous derivation of the Gross–Pitaevskii energy functional. Phys. Rev. A **61**, 685–697 (2000)

[18] Lieb, E., Yngvason, J.: Ground state energy of the low density Bose gas. Phys. Rev. Lett. **80**, 755–758 (1998)

[19] Nam, P., Napiórkowski, M., Ricaud, J., Triay, A.: Optimal rate of condensation for trapped bosons in the Gross–Pitaevskii regime. Anal. Partial Differ. Equ. **15**, 1585–1616 (2021)

[20] Seiringer, R.: The excitation spectrum for weakly interacting bosons. Commun. Math. Phys. **306**, 565–578 (2011)

[21] Wu, T.: Ground state of a Bose system of hard spheres. Phys. Rev. **115**, 1390–1404 (1959)

